# Copper-catalyzed monoselective C–H amination of ferrocenes with alkylamines

**DOI:** 10.3762/bjoc.17.165

**Published:** 2021-09-28

**Authors:** Zhen-Sheng Jia, Qiang Yue, Ya Li, Xue-Tao Xu, Kun Zhang, Bing-Feng Shi

**Affiliations:** 1 School of Biotechnology and Health Sciences, Wuyi University, Jiangmen, Guangdong, 529020, China; 2 Department of Chemistry, Zhejiang University, Hangzhou, Zhejiang, 310027, China; 3 Green Catalysis Center and College of Chemistry, Zhengzhou University, Zhengzhou, 450001, China; 4 School of Chemistry and Chemical Engineering, Henan Normal University, Xinxiang, He’nan, 453007, China

**Keywords:** amination, C–H activation, copper, ferrocene, mono-selectivity

## Abstract

A copper-catalyzed mono-selective C–H amination of ferrocenes assisted by 8-aminoquinoline is presented here. A range of amines, including bioactive molecules, were successfully installed to the *ortho*-position of ferrocene amides with high efficiency under mild conditions. A range of functionalized ferrocenes were compatible to give the aminated products in moderate to good yields. The gram-scale reaction was smoothly conducted and the directing group could be removed easily under basic conditions.

## Introduction

Ferrocene-based compounds have broad applications from asymmetric catalysis to medicinal discovery [1–8]. Therefore, the development of efficient methods to access multifunctional ferrocenes has attracted tremendous attention. Conventionally, functionalized ferrocenes were derived via electrophilic aromatic substitution mediated by strong Lewis acids or direct metalation using strong bases, such as alkyllithium reagents [3,9–12]. However, the above protocols generally proceeded under harsh conditions that led to poor functional group tolerance and generated stoichiometric amounts of waste.

**Scheme 1:**
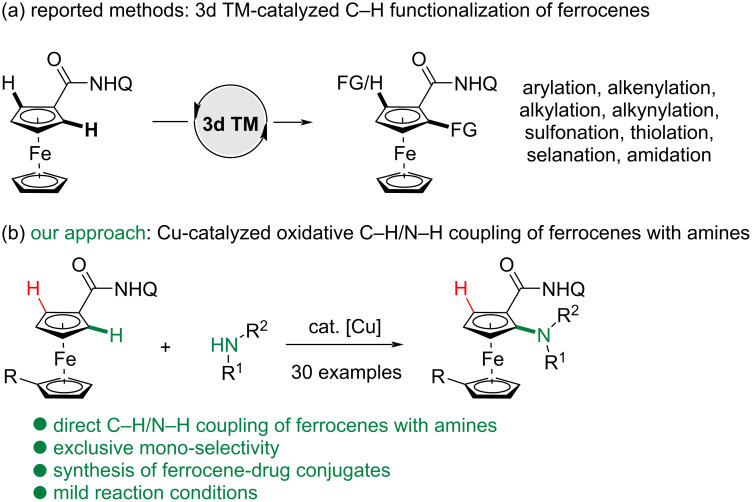
3d-Transition-metal-catalyzed C–H functionalization to access functionalized ferrocenes.

Thus far, the transition-metal-catalyzed C–H functionalization strategy has innovated the way to producing ferrocene derivatives [13–16]. Especially, the 3d transition metals, such as Cu, Co and Ni, have been exploited to convert C–H bonds to various functional groups, attributing to the cost-effective and less toxic properties, which render C–H transformations both economically desirable and environmentally benign (Scheme 1a) [17–22]. Early in 2015, the Ackermann group reported the first example of a low-valent Co-catalyzed C–H alkenylation of 2-pyridinylferrocene [23]. In 2017, the Butenschön group reported the *ortho*-C–H alkylation and arylation of ferrocene derivatives enabled by a combination of Fe or Co catalyst and *N*-containing directing groups, while an excess of Grignard reagents was used [24–25]. Thereafter, they also reported the Cp*Co-catalyzed *ortho*-C–H alkenylation of ferrocenes with alkynes [26] and the mono- and di-selectivity could be controlled by the fine-tuned directing groups. Kumar and co-workers developed a Cu-mediated C–H chalcogenation and sulfonation of ferrocenes [27–29]. The use of a bidentate 1,10-phenathroline ligand was critical to achieve mono-selectivity in the chacogenation reactions [28]. Meanwhile, Co(III)-catalyzed *ortho*-C–H amidation of ferrocene derivatives were also developed by the groups of You [30], Ackermann [31] and Shi [32–33] with 1,4,2-dioxazol-5-ones as versatile amidating reagents. In 2019, the alkynylated ferrocenes were isolated in the formation of alkyne-Cu(I) π-complexes by the Tan group via Cu-mediated C–H alkynylations [34]. Later in 2020, an enantioselective C–H annulation of ferrocenylformamides with alkynes was achieved by the Ye group enabled by Ni-Al bimetallic catalysis and a chiral secondary phosphine oxide (SPO) ligand [35]. Hou et al. also reported the asymmetric C−H alkenylation of quinoline- and pyridine-substituted ferrocenes with alkynes by using an unprecedented half-sandwich Sc catalyst [36]. Very recently, Shi and Zhang demonstrated a Cp*Co-catalyzed *ortho*-C–H allylation of ferrocenes assisted by thioamide using allyl carbonates and vinylcyclopropanes as allylating partners [37]. Meanwhile, Zhang and co-authors also reported the Co-catalyzed C–H alkoxylation of ferrocenes under nearly room temperature [38].

In comparison, despite the direct C–H amination of arenes with alkylamines has emerged as an efficient strategy to prepare substituted anilines [39–49], the application of this environmentally benign, oxidative coupling strategy to the synthesis of valuable *ortho-*amino ferrocene derivatives hasn’t been achieved [50], probably ascribing to several challenges. First, unprotected amines are sensitive and unendurable to several oxidants in the presence of transition metals. Second, both amines and the resulting aminated products could coordinate with metal catalysts and cause the deactivation of catalysts. Besides, high reaction temperature could lead to a mixture of byproducts or the decomposition of the ferrocene products. Herein, we described a Cu-catalyzed oxidative C–H/N–H coupling of ferrocenes with free amines to provide mono-aminated ferrocenes exclusively under mild conditions (Scheme 1b). During the preparation of the manuscript of this article, a nice report on Cu-catalyzed C–H amination of ferrocenes directed by 8-aminoquinoline was reported by Fukuzawa and Kanemoto [50]. Notably, our method proceeded under silver-free conditions at relatively lower temperature and in shorter time, providing a complementary alternative to the work of Fukuzawa and Kanemoto.

## Results and Discussion

We initiated our study by investigating the C–H amination of ferrocene carboxylic amide **1a** with morpholine (**2a**) using 8-amonoquinoline as directing group [51–56]. The *ortho*-aminated ferrocenylamide **3a** was isolated in 11% yield in the presence of CuI, *N*-methylmorpholine *N*-oxide (NMO) and K_2_CO_3_ in DMF (Table 1, entry 1). When the reaction was conducted in MeCN, the yield could be improved to 32% (Table 1, entry 4). Further screening of other oxidants revealed that NMO was the optimal (Table 1, entries 9–11). When the reaction was conducted in neat in the presence of 2-pyridone, **3a** was obtained in 36% yield (Table 1, entry 12). However, significant decomposition of the aminated product **3a** was observed. Consequently, we exclusively evaluated the reaction temperature and time (Table 1, entries 13–16). To our delight, **3a** could be obtained in 80% yield under relatively lower temperature (80 °C) and shorter time (4 hours). To note, this reaction showed excellent mono-selectivity and no diaminated ferrocenylamide was detected. The exclusive monoselectivity is most likely originated from the strong coordination of the amino group, which could form a tridentate copper complex and prevent the second C–H amination [34,50].

**Table 1:** Optimization of reaction conditions.^a^

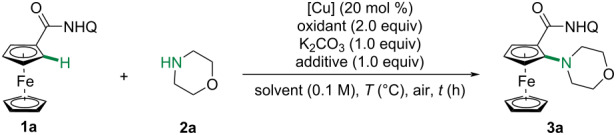							

Entry	[Cu]	Solvent	Oxidant	Additive	*T* (°C)	*t* (h)	Yield^b^

1	CuI	DMF	NMO	–	120	12	11%
2	CuI	NMP	NMO	–	120	12	15%
3	CuI	DMSO	NMO	–	120	12	trace
4	CuI	MeCN	NMO	–	120	12	32%
5	Cu(OAc)_2_	MeCN	NMO	–	120	12	10%
6	CuCN	MeCN	NMO	–	120	12	12%
7	CuCl	MeCN	NMO	–	120	12	18%
8	CuTc	MeCN	NMO	–	120	12	trace
9	CuI	MeCN	TEMPO	–	120	12	23%
10	CuI	MeCN	MnO_2_	–	120	12	trace
11^c^	CuI	MeCN	O_2_	–	120	12	8%
12^d^	CuI	neat	NMO	2-pyridone	120	12	36%
13^d^	CuI	neat	NMO	2-pyridone	100	12	46%
14^d^	CuI	neat	NMO	2-pyridone	80	12	56%
15^d^	CuI	neat	NMO	2-pyridone	80	6	68%
16^d^	CuI	neat	NMO	2-pyridone	80	4	80%

^a^Reactions conditions: **1a** (0.1 mmol), **2a** (0.3 mmol), [Cu] (20 mol %), oxidant (2.0 equiv), K_2_CO_3_ (1.0 equiv) and additive (1.0 equiv) in a sealed tube. ^b^Isolated yield. ^c^Oxygen balloon. ^d^5.0 equiv of morpholine.

**Scheme 2:**
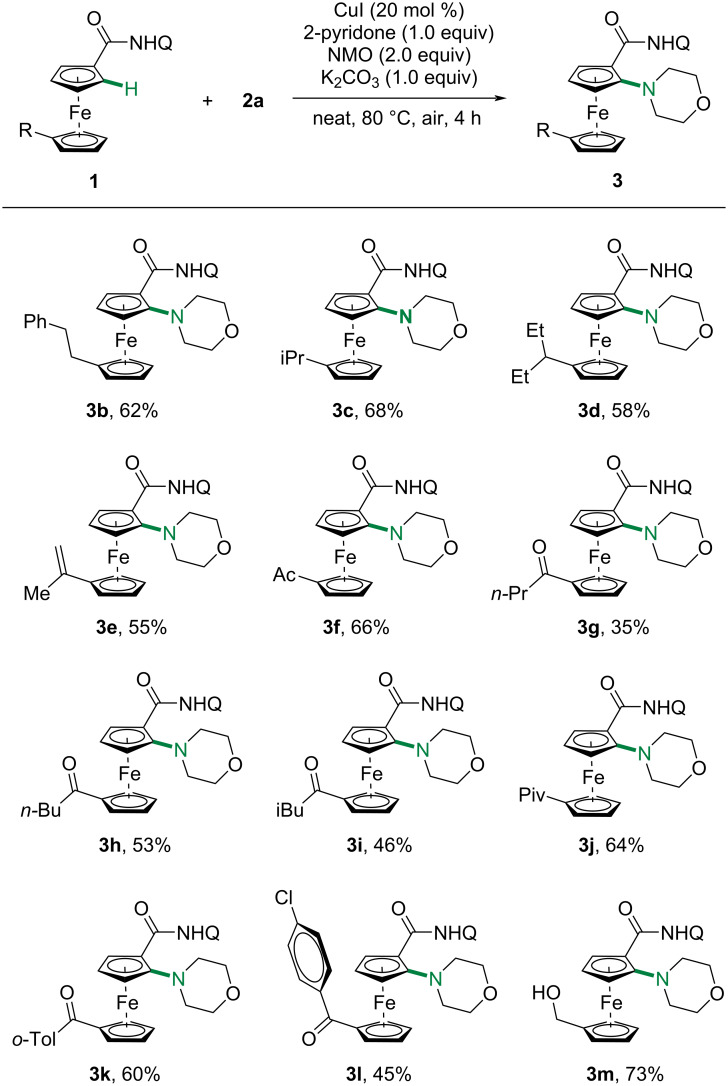
Scope of ferrocenes with morpholine.

Having obtained the optimized reaction conditions, we started to investigate the generality of this C–H amination protocol with regard to modified ferrocenes (Scheme 2). Delightfully, a variety of functional groups tailored to the other cyclopentadienyl (Cp) moieties were well tolerated, furnishing the desired *ortho*-mono-aminating ferrocenes in moderate to good yields. Alkyl substituents on the other Cp ring of ferrocenylamides only showed slightly effects (**3b**–**d**). Notably, the terminal alkenyl group of **3e** was well tolerated during the amination process. Weakly coordinating carbonyl groups were also tolerated and the desired C–H amination occurred selectively at the *ortho* position to the *N*-quinolinyl amides with acceptable yields (**3f**–**l**). Notably, free alcohol was also compatible with this protocol, exclusively giving the mono-aminated product in 73% yield (**3m**) without the observation of any competitive alkoxylation product [38,57–58].

**Scheme 3:**
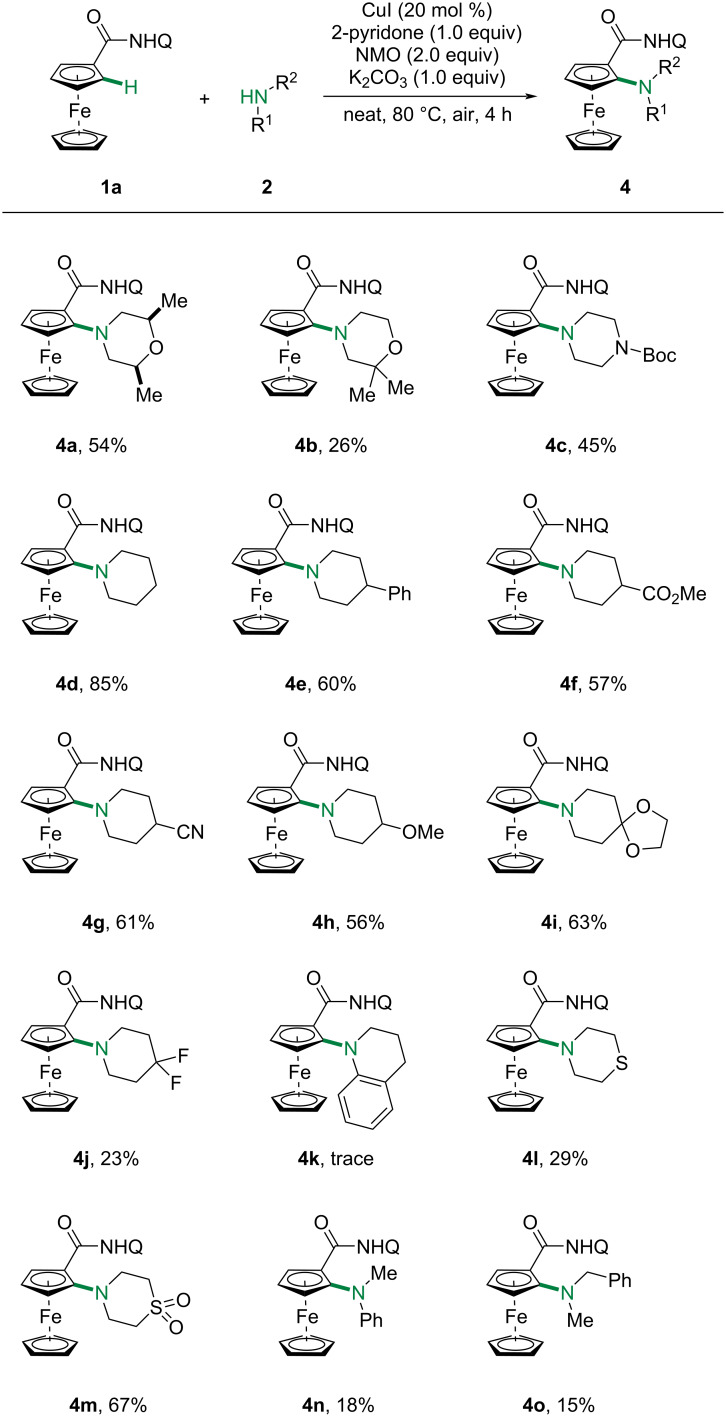
Scope of various amines with **1a**.

We then explored the scope of multifarious amines. As displayed in Scheme 3, a range of cyclic amines, such as morpholine **4a**,**b**, piperazine **4c**, piperidine **4d**–**j** and thiomorpholine **4l**,**m**, reacted smoothly to give the amination products in 23% to 85% yields. A variety of synthetically useful functionalities, such as ester **4f**, cyano **4g** and ketal **4i**, were well tolerated. Unfortunately, 1,2,3,4-tetrahydroquinoline (**2k**) was proved unreactive under our conditions, probably due to the steric hindrance. Thiomorpholine (**2l**) was compatible with this reaction, albeit with significantly dropped yield (29%), largely due to the poison of copper catalyst by thioether. Acyclic amines were also tested and the amination products were obtained in low yields (**4n**, 18%; **4o**, 15%). Unfortunately, primary amines and anilines were completely inert.

**Scheme 4:**
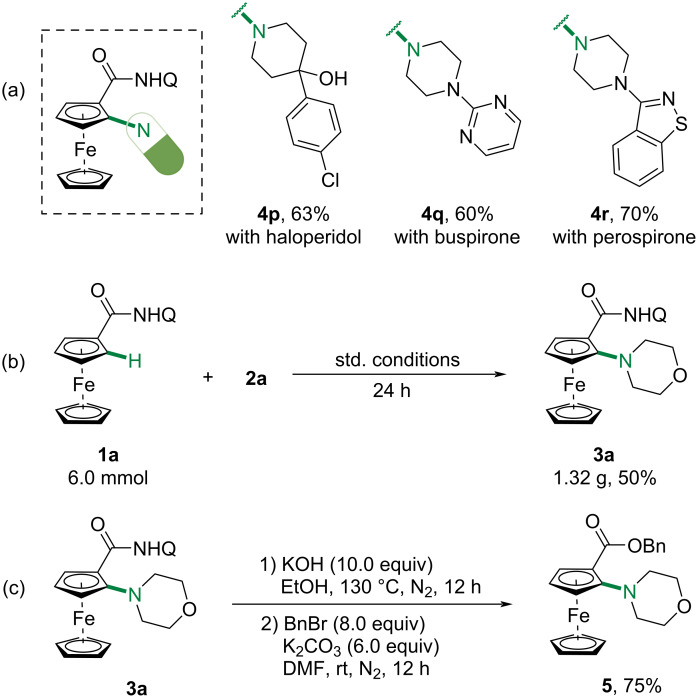
Synthetic applications.

Encouraged by the above results, we further tried to synthesize ferrocene–drug conjugates (Scheme 4a). Three amines used to treat psychosis were subjected to couple with **1a** and the desired conjugates were obtained in good yields (with haloperidol, **4p**, 63%; with buspirone, **4q**, 60%; with perospirone, **4r**, 70%). This protocol was also amendable to gram-scale synthesis, giving **3a** in 50% yield (Scheme 4b, 1.32 g). For synthetic utility, the directing group was conveniently removed by refluxing with KOH in EtOH and the benzyl-protected ester **5** was obtained in 75% yield.

**Scheme 5:**
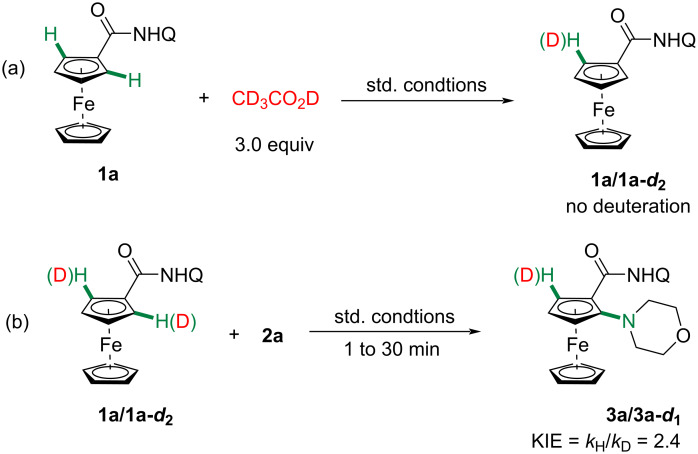
Mechanistic experiments.

We also conducted several deuteration experiments to shed a preliminary insight into the mechanism. No H/D exchange was observed at the *ortho*-position of **1a** with 3.0 equivalents of CD_3_CO_2_D under standard conditions (Scheme 5a). Furthermore, a larger value of kinetic isotope effect (KIE = 2.4) was detected (Scheme 5b). These results indicated that the cleavage of C–H bond was most likely involved in the rate-determining step.

## Conclusion

To summarize, we have reported a copper-catalyzed direct *ortho*-C–H/N–H coupling reaction of ferrocenes with alkyl amines directed by 8-aminoquinoline. Fruitful mono-aminated ferrocenes were obtained in moderate to good yields and the mild conditions offered the possibility to the preparation of ferrocene–drug conjugates effectively. Mechanistic studies indicated that the C–H activation step was the rate-determining step.

## Supporting Information

File 1 Full experimental details, compound characterization, and copies of NMR spectra.

## References

[1] Hayashi T, Togni A (1995). Ferrocenes.

[2] Štěpnička P (2008). Ferrocenes.

[3] Dai L-X, Hou X-L (2010). Chiral Ferrocenes in Asymmetric Catalysis.

[4] van Staveren D R, Metzler-Nolte N (2004). Chem Rev.

[5] Gómez Arrayás R, Adrio J, Carretero J C (2006). Angew Chem, Int Ed.

[6] Hartinger C G, Dyson P J (2009). Chem Soc Rev.

[7] Gasser G, Ott I, Metzler-Nolte N (2011). J Med Chem.

[8] Toma Š, Csizmadiová J, Mečiarová M, Šebesta R (2014). Dalton Trans.

[9] Tsukazaki M, Tinkl M, Roglans A, Chapell B J, Taylor N J, Snieckus V (1996). J Am Chem Soc.

[10] Metallinos C, Szillat H, Taylor N J, Snieckus V (2003). Adv Synth Catal.

[11] Butt N A, Liu D, Zhang W (2014). Synlett.

[12] Schaarschmidt D, Lang H (2013). Organometallics.

[13] López L A, López E (2015). Dalton Trans.

[14] Zhu D-Y, Chen P, Xia J-B (2016). ChemCatChem.

[15] Gao D-W, Gu Q, Zheng C, You S-L (2017). Acc Chem Res.

[16] Huang J, Gu Q, You S (2018). Chin J Org Chem.

[17] Gandeepan P, Müller T, Zell D, Cera G, Warratz S, Ackermann L (2019). Chem Rev.

[18] Loup J, Dhawa U, Pesciaioli F, Wencel‐Delord J, Ackermann L (2019). Angew Chem, Int Ed.

[19] Woźniak Ł, Cramer N (2019). Trends Chem.

[20] Rao W-H, Shi B-F (2016). Org Chem Front.

[21] Liu J, Chen G, Tan Z (2016). Adv Synth Catal.

[22] Liu Y-H, Xia Y-N, Shi B-F (2020). Chin J Chem.

[23] Moselage M, Sauermann N, Richter S C, Ackermann L (2015). Angew Chem, Int Ed.

[24] Schmiel D, Butenschön H (2017). Eur J Org Chem.

[25] Schmiel D, Butenschön H (2017). Organometallics.

[26] Schmiel D, Gathy R, Butenschön H (2018). Organometallics.

[27] Sattar M, Shareef M, Patidar K, Kumar S (2018). J Org Chem.

[28] Sattar M, Patidar K, Thorat R A, Kumar S (2019). J Org Chem.

[29] Sattar M, Kumar N, Yadav P, Mandhar Y, Kumar S (2019). Chem – Asian J.

[30] Wang S-B, Gu Q, You S-L (2018). J Catal.

[31] Yetra S R, Shen Z, Wang H, Ackermann L (2018). Beilstein J Org Chem.

[32] Huang D-Y, Yao Q-J, Zhang S, Xu X-T, Zhang K, Shi B-F (2019). Org Lett.

[33] Liu Y-H, Li P-X, Yao Q-J, Zhang Z-Z, Huang D-Y, Le M D, Song H, Liu L, Shi B-F (2019). Org Lett.

[34] Song Z, Yu Y, Yu L, Liu D, Wu Q, Xia Z, Xiao Y, Tan Z (2019). Organometallics.

[35] Chen H, Wang Y-X, Luan Y-X, Ye M (2020). Angew Chem, Int Ed.

[36] Lou S-J, Zhuo Q, Nishiura M, Luo G, Hou Z (2021). J Am Chem Soc.

[37] Zhang Z-Z, Liao G, Chen H-M, Shi B-F (2021). Org Lett.

[38] Zhang Z-Z, Cheng J, Yao Q-J, Yue Q, Zhou G (2021). Adv Synth Catal.

[39] Kim H, Chang S (2016). ACS Catal.

[40] Tran L D, Roane J, Daugulis O (2013). Angew Chem, Int Ed.

[41] Matsubara T, Asako S, Ilies L, Nakamura E (2014). J Am Chem Soc.

[42] Yan Q, Chen Z, Yu W, Yin H, Liu Z, Zhang Y (2015). Org Lett.

[43] Roane J, Daugulis O (2016). J Am Chem Soc.

[44] Yan Q, Xiao T, Liu Z, Zhang Y (2016). Adv Synth Catal.

[45] Gao X, Wang P, Zeng L, Tang S, Lei A (2018). J Am Chem Soc.

[46] Zhang S-K, Samanta R C, Sauermann N, Ackermann L (2018). Chem – Eur J.

[47] Kathiravan S, Suriyanarayanan S, Nicholls I A (2019). Org Lett.

[48] Shang M, Shao Q, Sun S-Z, Chen Y-Q, Xu H, Dai H-X, Yu J-Q (2017). Chem Sci.

[49] Wu P, Huang W, Cheng T-J, Lin H-X, Xu H, Dai H-X (2020). Org Lett.

[50] Kanemoto K, Horikawa N, Hoshino S, Tokoro Y, Fukuzawa S-i (2021). Org Lett.

[51] Zaitsev V G, Shabashov D, Daugulis O (2005). J Am Chem Soc.

[52] Daugulis O, Roane J, Tran L D (2015). Acc Chem Res.

[53] Sambiagio C, Schönbauer D, Blieck R, Dao-Huy T, Pototschnig G, Schaaf P, Wiesinger T, Zia M F, Wencel-Delord J, Besset T (2018). Chem Soc Rev.

[54] Zhang Q, Shi B-F (2019). Chin J Chem.

[55] Rej S, Ano Y, Chatani N (2020). Chem Rev.

[56] Zhang Q, Shi B-F (2021). Acc Chem Res.

[57] Roane J, Daugulis O (2013). Org Lett.

[58] Hu Y, Wang M, Li P, Li H, Wang L (2019). Asian J Org Chem.

