# Supplementary material for: Copper-catalyzed monoselective C–H amination of ferrocenes with alkylamines
Source: Beilstein J Org Chem. 2021 Sep 28;17:2488–95. doi: 10.3762/bjoc.17.165 (PMC8491713; doi:10.3762/bjoc.17.165)

BEILSTEIN JOURNAL OF ORGANIC CHEMISTRY

# Supporting Information

for

# Copper-catalyzed monoselective C–H amination of ferrocenes with alkylamines

Zhen-Sheng Jia, Qiang Yue, Ya Li, Xue-Tao Xu, Kun Zhang and Bing-Feng Shi

*Beilstein J. Org. Chem.* **2021,** *17,* 2488–2495. doi:10.3762/bjoc.17.165

# Full experimental details, compound characterization, and copies of NMR spectra

License and Terms: This is a supporting information file under the terms of the Creative Commons Attribution License (https://creativecommons.org/licenses/by/4.0). Please note that the reuse, redistribution and reproduction in particular requires that the author(s) and source are credited and that individual graphics may be subject to special legal provisions.
The license is subject to the *Beilstein Journal of Organic Chemistry* terms and conditions: (https://www.beilstein-journals.org/bjoc/terms)

## Table of contents


1. General information ........................................ S2
2. Structure of substrates ........................................ S3
3. Experimental section ........................................ S4
4. Screening of reaction conditions ........................................ S17
5. General procedures for Cu-catalyzed monoselective C–H amination of ferrocenes with alkylamines ........................................ S21
6. Gram-scale synthesis ........................................ S38
7. Removal of directing group. ........................................ S39
8. Mechanistic experiments. ........................................ S40
9. References. ........................................ S43
10. NMR spectra. ........................................ S44

# 1. General information

All the materials and solvents were purchased from commercial suppliers and used without additional purification. CuI was purchased from Strem. NMR spectra were recorded on a Bruke Avance operating for $^1H$ NMR at 400 MHz, $^{13}C$ NMR at 100 MHz using TMS as internal standard. The peaks were internally referenced to $CDCl_3$ (7.26 ppm) or residual undeuterated solvent signal of $CDCl_3$ (77.16 ppm for $^{13}C$ NMR). The following abbreviations (or combinations thereof) were used to explain multiplicities: s = singlet, d = doublet, t = triplet, m = multiplet, brs = broad singlet. Mass spectroscopy data of the products were collected on an HRMS-TOF instrument using ESI ionization.

## 2. Structure of substrates

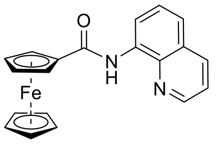

**1a**

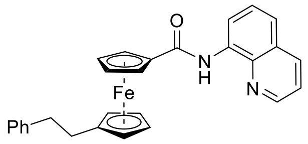

**1b**

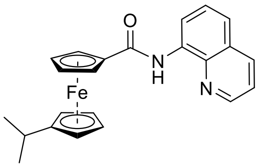

**1c**

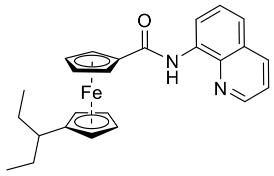

**1d**

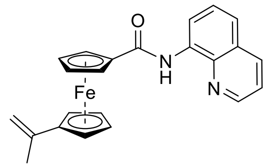

**1e**

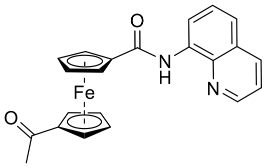

**1f**

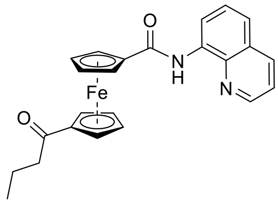

**1g**

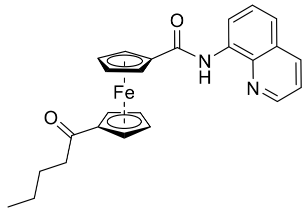

**1h**

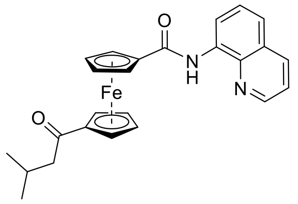

**1i**

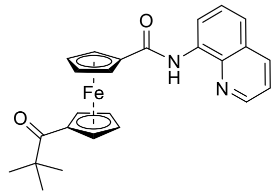

**1j**

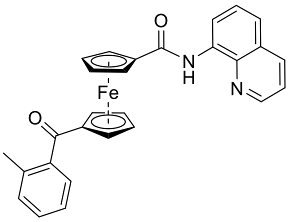

**1k**

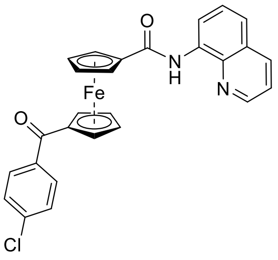

**1l**

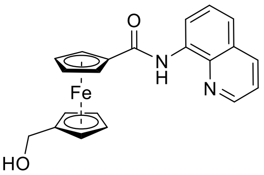

**1m**

**Figure S1:** Ferrocene substrates

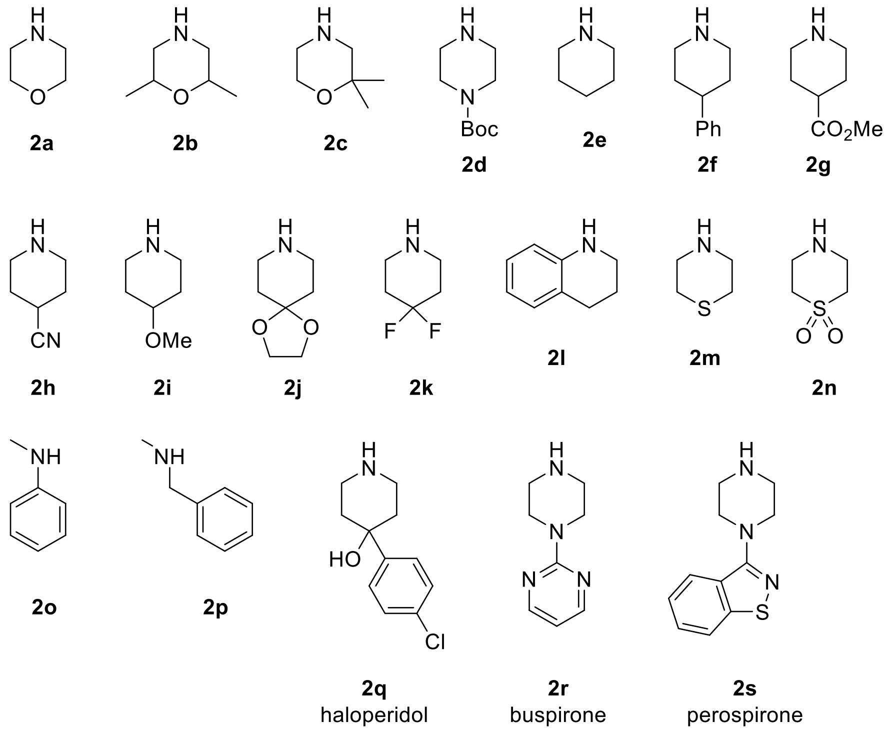


**Figure S2:** Secondary amines

# 3. Experimental section

## 3-1. Preparation of substrates

Substrate **1a** is a known compound and was prepared according to the literature.[1] Substrates **1b–m** were prepared following the literature procedure. Amines **2a–s** are commercially available. All other starting materials, solvents and reagents were purchased and used as received.

**General procedure 1 (for the preparation of 1f–l):**

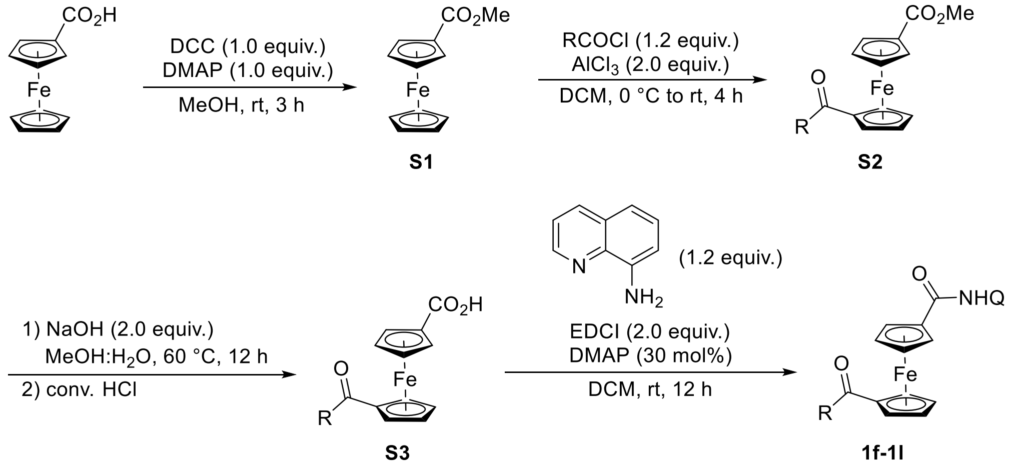

*N*,*N'*-Dicyclohexylcarbodiimide (DCC, 5.0 mmol, 0.91 g) and 4-dimethylaminopyridine (DMAP, 5.0 mmol, 0.54 g) were added to the solution of ferrocenecarboxylic acid (5.0 mmol, 1.01 g) in dry MeOH (20 mL). The reaction mixture was flushed with argon and stirred at room temperature for 3 h. The reaction mixture was concentrated and the residue was purified by flash chromatography on silica gel (ethyl acetate/hexanes = 1/9) to give ferrocenecarboxylic acid methyl ester **S1**[2] as orange crystals.

Acetyl chloride (0.43 mL, 6.0 mmol) was added to a suspension of anhydrous aluminum chloride (1.33 g, 10 mmol) in $CH_2Cl_2$ (12 mL) at 0 °C and stirred for 15 min. The mixture was then added to a solution of methyl ferrocene-1-carboxylate (1.0 g, 5.0 mmol) in $CH_2Cl_2$ (23 mL) at 0 °C over a period of 15 min upon stirring under nitrogen atmosphere. To prevent light-induced degradation, the flask was covered with aluminum foil during the reaction. After 4 h, the reaction mixture was poured into ice water and the resulting precipitate was solubilized by addition of concentrated HCl. The organic layer was separated, and the aqueous layer was extracted with $CH_2Cl_2$. The combined organic layers were dried ($MgSO_4$) and evaporated to give **S2**[3] as orange solid.

A solution of the ester **S2** (3.0 mmol) in 15 mL of methanol and 15 mL of 20% NaOH (aq.) was heated under reflux for 12 h. The reaction mixture was cooled and acidified with 5 mL of 20% HCl in an ice bath and then stirred vigorously for a few minutes to make the free acid from the sodium salt. The product was collected by suction filtration, washed with 10 mL of water, and dried. Crude ferrocene carboxylic acid **S3** was obtained and used directly for the next step.

Following a reported procedure:[4] In a 50 mL Round-bottomed flask, to a solution of **S3** (3.0 mmol), 8-aminoquinoline (3.6 mmol), and DMAP (111 mg, 0.9 mmol) in anhydrous $CH_2Cl_2$ (15 mL) was added a solution of *N*-ethyl-*N'*-(3-dimethylaminopropyl)carbodiimide hydrochloride (EDCI, 1.15 g, 6 mmol) in $CH_2Cl_2$ (15 mL) through a dropping funnel at 0 °C under $N_2$ atmosphere. The reaction mixture was stirred at rt overnight. After completion, the mixture was quenched with $NaHCO_3$ (10 mL) and diluted with $CH_2Cl_2$ (50 mL). The organic layer was washed with aq. 1 N HCl (50 mL), followed by aq. $NaHCO_3$ (50 mL), aq. NaCl (50 mL), and dried with anhydrous $Na_2SO_4$. The solvent was concentrated and the resulting residue was purified by column chromatography using EtOAc/hexane as eluent to afford the desired product **1f–l** as an orange solid.

**General procedure 2 (for the preparation of 1c and 1d):**

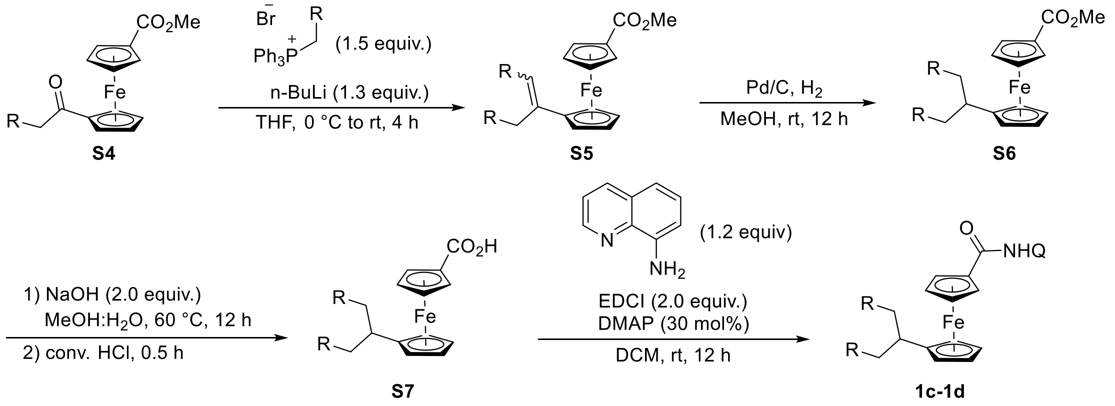


To an oven dried round bottom flask equipped with a stir bar under a $N_2$(g) atmosphere was added $PPh_3MeBr$ (1.50 equiv). The flask was evacuated and back filled with $N_2$(g) and dry THF (0.1 M) was added. The resultant mixture was cooled to −78 °C, to which a solution of n-BuLi in hexanes (1.6 M, 1.3 equiv) was added. The solution was allowed to warm to rt and stirred for 30 mins before ketone substrate **S4** was added (1.0 equiv). The reaction was allowed to stir at rt for 4 h before being diluted with hexane and quenched with $H_2O$. The aqueous layer was separated and washed with hexane (3 × 30 mL). The organic layers were combined, dried ($Na_2SO_4$), filtered and concentrated in vacuo. The resultant crude alkyferrocenes **S5** were purified via column chromatography

To a solution of **S5** (4 mmol, 1 equiv) in MeOH (20 mL) was added Pd/C (10 wt %) and the suspension was equipped with $H_2$ (1 atm) several times. After stirring at rt. for 12 h, the reaction mixture was filtered and the filtrate was concentrated to afford the title compound **S6** as orange oil and used directly in the next step.

A solution of the ester **S6** (3.0 mmol) in 15 mL of methanol and 15 mL of 20% NaOH (aq.) was heated under reflux for 12 h. The reaction mixture was cooled and acidified with 5 mL of 20% HCl in an ice bath and then stirred vigorously for a few minutes to make free acid from the sodium salt. The product was collected by suction filtration at room temperature, washed with 10 mL of water, and dried. Pure 8-ferrocenyl-8-oxooctanoic acid **S7** as red solid.

Following a reported procedure:[4] In a 50 mL Round-bottomed flask, to a solution of **S7** (3.0 mmol), 8-aminoquinoline (3.6 mmol), and DMAP (111 mg, 0.9 mmol) in anhydrous $CH_2Cl_2$ (15 mL) was added a solution of *N*-ethyl-*N'*-(3-dimethylaminopropyl)carbodiimide hydrochloride (EDCI, 1.15 g, 6 mmol) in $CH_2Cl_2$ (15 mL) through a dropping funnel at 0 °C under $N_2$ atmosphere. The reaction mixture was stirred at rt overnight. After completion, the mixture was quenched with

$NaHCO_3$ (10 mL) and diluted with $CH_2Cl_2$ (50 mL). The organic layer was washed with aq. 1 N HCl (50 mL), followed by aq. $NaHCO_3$ (50 mL), aq. NaCl (50 mL), and dried with anhydrous $Na_2SO_4$. The solvent was concentrated and the resulting residue was purified by column chromatography using EtOAc/hexane as eluent to afford the desired product **1c/1d** as an orange solid.

**General procedure 3:**

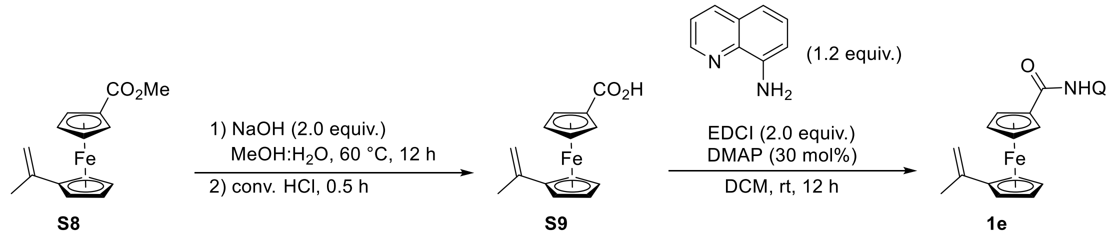


A solution of the ester **S8** (0.85 g, 3.0 mmol) in 15 mL of methanol and 15 mL of 20% NaOH (aq.) was heated under reflux for 12 h. The reaction mixture was coo led and acidified with 5 mL of 20% HCl in an ice bath and then stirred vigorously for a few minutes to make free acid from the sodium salt. The product **S9** was collected by suction filtration at room temperature, washed with 10 mL of water, and dried.

Following a reported procedure:[4] In a 50 mL Round-bottomed flask, to a solution of **S9** (3.0 mmol), 8-aminoquinoline (3.6 mmol), and DMAP (111 mg, 0.9 mmol) in anhydrous $CH_2Cl_2$ (15 mL) was added a solution of *N*-ethyl-*N*'-(3-dimethylaminopropyl)carbodiimide hydrochloride (EDCI, 1.15 g, 6 mmol) in $CH_2Cl_2$ (15 mL) through a dropping funnel at 0 °C under $N_2$ atmosphere. The reaction mixture was stirred at rt overnight. After completion, the mixture was quenched with $NaHCO_3$ (10 mL) and diluted with $CH_2Cl_2$ (50 mL). The organic layer was washed with aq. 1 N HCl (50 mL), followed by aq. $NaHCO_3$ (50 mL), aq. NaCl (50 mL), and dried with anhydrous $Na_2SO_4$. The solvent was concentrated and the resulting residue was purified by column chromatography using EtOAc/hexane as eluent to afford the desired product **1e** as a yellow solid.

**General Procedure 4:**

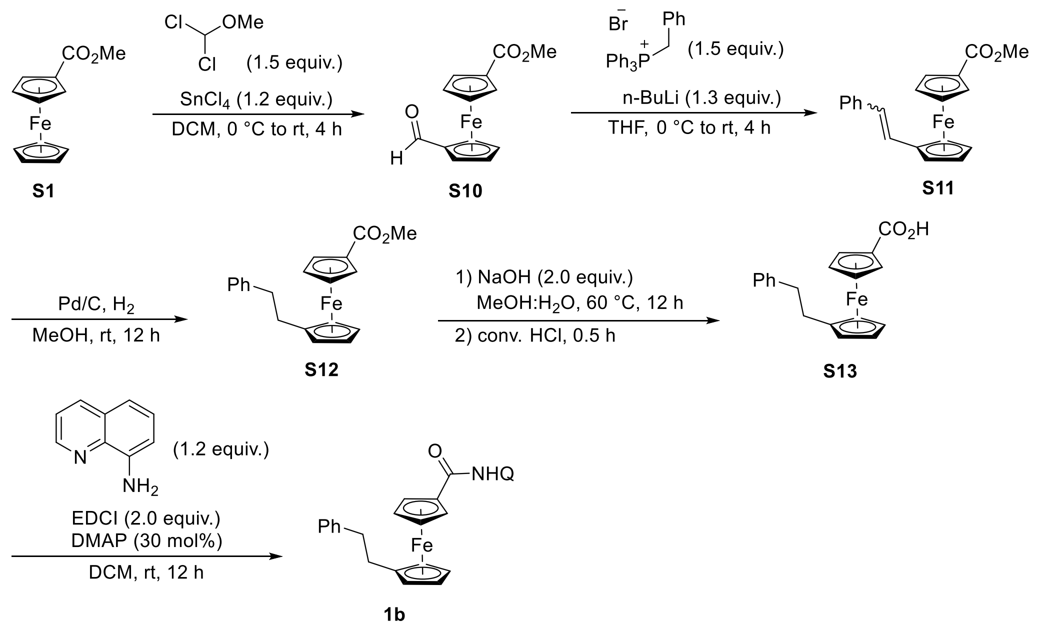


1,1-Dichlorodimethyl ether (15 mmol) was added at 0 °C to a suspension of anhydrous tin tetrachloride (12 mmol) in $CH_2Cl_2$ (12 mL) and stirred for 15 min. The mixture was then added at 0 °C to a solution of **S1** (2.4 g, 10 mmol) in $CH_2Cl_2$ (23 mL) over a period of 15 min upon stirring under nitrogen atmosphere. After 4 h, the reaction mixture was poured into ice water and the resulting precipitate was solubilized by addition of concentrated HCl. The organic layer was separated, and the aqueous layer was extracted with $CH_2Cl_2$. The combined organic layers were dried ($MgSO_4$) and evaporated under reduced pressure to give **S10** as an orange solid.

To an oven dried round bottom flask equipped with a stir bar under a $N_2$(g) atmosphere was added $PPh_3BnBr$ (1.50 equiv). The flask was evacuated and back filled with $N_2$(g) and dry THF (0.1 M) was added. The resultant mixture was cooled to −78 °C to which a solution of n-BuLi in hexanes (1.6M, 1.3 equiv) was added. The solution was allowed to warm to rt and stirred for 30 mins before the crude **S10** was added (1.0 equiv). The reaction was allowed to stir at rt for 4 h before diluted with hexane and quenched with $H_2O$. The aqueous layer was separated and washed with hexane (3 × 30 mL). The organic layers were combined, dried ($Na_2SO_4$), filtered and concentrated in vacuo. The resultant crude alkenes were purified *via* column chromatography, afford the desired **S11** as an orange solid.

To a solution of **S11** (4 mmol, 1 equiv) in MeOH (20 mL) was added Pd/C (10 wt %) and the

suspension was equipped with $H_2$ (1 atm) several times. After stirring at rt. for 12 h, the reaction mixture was filtered and the filtrate was concentrated to afford the title compound **S12** as an orange oil and used directly in the next step.

A solution of the ester **S12** (1.04 g, 3.0 mmol) in 15 mL of methanol and 15 mL of 20% NaOH (aq.) was heated under reflux for 12 h. The reaction mixture was cooled and acidified with 5 mL of 20% HCl in an ice bath and then stirred vigorously for a few minutes to make free acid from the sodium salt. The crude product **S13** was collected by suction filtration at room temperature, washed with 10 mL of water, and dried.

Following a reported procedure:[4] In a 50 mL Round-bottomed flask, to a solution of **S13** (3.0 mmol), 8-aminoquinoline (3.6 mmol), and DMAP (111 mg, 0.9 mmol) in anhydrous $CH_2Cl_2$ (15 mL) was added a solution of *N*-ethyl-*N'*-(3-dimethylaminopropyl)carbodiimide hydrochloride (EDCI, 1.15 g, 6 mmol) in $CH_2Cl_2$ (15 mL) through a dropping funnel at 0 °C under $N_2$ atmosphere. The reaction mixture was stirred at rt overnight. After completion, the mixture was quenched with $NaHCO_3$ (10 mL) and diluted with $CH_2Cl_2$ (50 mL). The organic layer was washed with aq. 1 N HCl (50 mL), followed by aq. $NaHCO_3$ (50 mL), aq. NaCl (50 mL), and dried with anhydrous $Na_2SO_4$. The solvent was concentrated and the resulting residue was purified by column chromatography using EtOAc/hexane as eluent to afford the desired product **1b** as an orange solid.

## General Procedure 5:

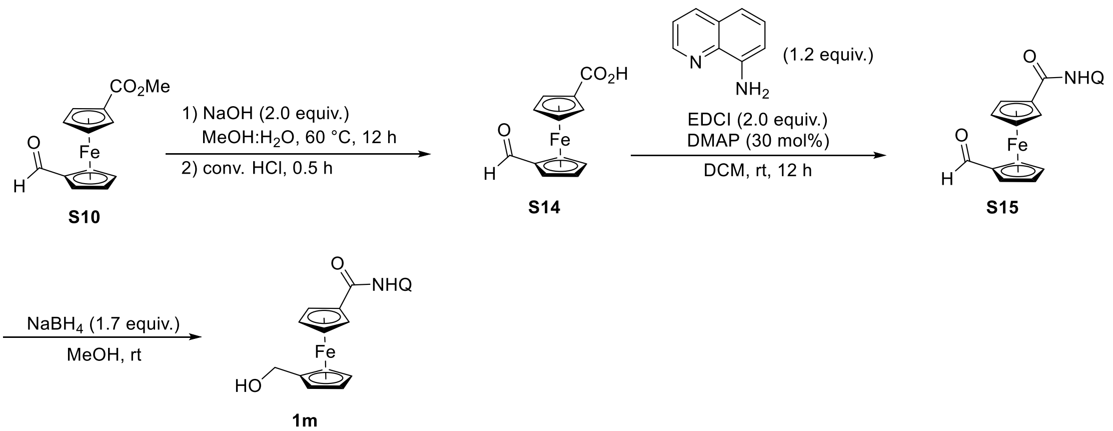


A solution of the ester **S10** (0.82 g, 3.0 mmol) in 15 mL of methanol and 15 mL of 20% NaOH (aq.) was heated under reflux for 12 h. The reaction mixture was cooled and acidified with 5 mL of 20% HCl in an ice bath and then stirred vigorously for a few minutes to make free acid from the sodium salt. The crude product S**14** was collected by suction filtration at room temperature, washed

with 10 mL of water, and dried.

Following a reported procedure:[4] In a 50 mL Round-bottomed flask, to a solution of **S14** (3.0 mmol), 8-aminoquinoline (3.6 mmol), and DMAP (111 mg, 0.9 mmol) in anhydrous $CH_2Cl_2$ (15 mL) was added a solution of *N*-ethyl-*N'*-(3-dimethylaminopropyl)carbodiimide hydrochloride (EDCI, 1.15 g, 6 mmol) in $CH_2Cl_2$ (15 mL) through a dropping funnel at 0 °C under $N_2$ atmosphere. The reaction mixture was stirred at rt overnight. After completion, the mixture was quenched with $NaHCO_3$ (10 mL) and diluted with $CH_2Cl_2$ (50 mL). The organic layer was washed with aq. 1 N HCl (50 mL), followed by aq. $NaHCO_3$ (50 mL), aq. NaCl (50 mL), and dried with anhydrous $Na_2SO_4$. The solvent was concentrated and the resulting residue was purified by column chromatography using EtOAc/hexane as eluent to afford the desired product **S15** as a yellow solid.

To a solution of compound **S15** (3 mmol) in methanol (10 mL) was added $NaBH_4$ (0.2 g, 5 mmol). After stirring for 1 h while cooled with an ice-water bath, methanol was evaporated and the residue was dissolved in EtOAc (50 mL). The organic layer was washed with water (3×30 mL) and brine (3×30 mL), and dried over $MgSO_4$. The volatiles were removed to give pure **1m** as a yellow solid.

**<u>1-((8-Quinolinylamino)carbonyl)-1'-phenethyl-ferrocene (2b)</u>**

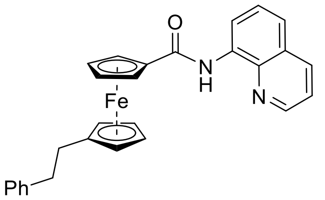


Substrate **2b** was synthesized according to **General Procedure 4**; orange solid.

**$^1$H NMR (400 MHz, Chloroform-*d*)** δ 10.26 (s, 1H), 8.85 – 8.79 (m, 1H), 8.77 (dd, *J* = 4.2, 1.6 Hz, 1H), 8.18 (dd, *J* = 8.2, 1.6 Hz, 1H), 7.57 (t, *J* = 7.8 Hz, 1H), 7.51 (d, *J* = 8.1 Hz, 1H), 7.46 (dd, *J* = 8.3, 4.2 Hz, 1H), 7.17 (m, *J* = 7.8, 6.9, 3.7 Hz, 3H), 7.06 – 7.00 (m, 2H), 4.91 (s, 2H), 4.43 (s, 2H), 4.19 (d, *J* = 1.9 Hz, 2H), 4.14 (d, *J* = 2.0 Hz, 2H), 2.74 (m, *J* = 9.3, 5.5 Hz, 2H), 2.65 (m, *J* = 10.2, 6.2 Hz, 2H).

**$^{13}$C NMR (100 MHz, $CDCl_3$)** δ 169.1, 148.4, 141.9, 138.6, 136.5, 134.9, 128.4, 128.3, 128.2, 127.7, 125.9, 121.7, 121.1, 116.3, 90.4, 71.6, 70.1, 69.3, 69.2, 37.6, 30.7.

**HRMS (ESI)** m/z: $[M + Na]^+$ Calcd for $C_{28}H_{24}FeN_2ONa$ 483.1130; found:483.1133.

**1-((8-Quinolinylamino)carbonyl)-1'-isopropyl-ferrocene (2c)**

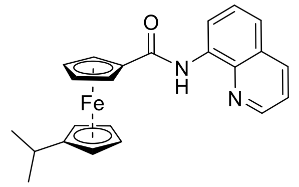

Substrate **2c** was synthesized according to **General Procedure 2**; orange solid.

**$^{1}$H NMR (400 MHz, Chloroform-*d*)** δ 10.28 (d, *J* = 4.7 Hz, 1H), 8.95 – 8.84 (m, 1H), 8.82 (dd, *J* = 7.8, 3.4 Hz, 1H), 8.19 (d, *J* = 8.0, 2.4, 1.9 Hz, 1H), 7.64 – 7.53 (m, 1H), 7.49 (m, *J* = 12.4, 3.8 Hz, 2H), 4.92 (p, *J* = 2.4, 1.9 Hz, 2H), 4.44 (p, *J* = 2.2 Hz, 2H), 4.21 (p, *J* = 2.1, 1.7 Hz, 2H), 4.15 (p, *J* = 2.2 Hz, 2H), 2.67 (m, *J* = 10.1, 8.9, 7.1, 4.9 Hz, 1H), 1.22 – 1.13 (m, 6H).

**$^{13}$C NMR (100 MHz, $CDCl_3$)** δ 169.3, 148.3, 138.6, 136.5, 134.9, 128.2, 127.7, 121.7, 121.0, 116.2, 98.8, 71.5, 69.3, 69.0, 68.1, 27.3, 23.7.

**HRMS (ESI)** m/z: $[M + H]^+$ Calcd for $C_{23}H_{23}FeN_2O$ 399.1082; found: 399.1153.

**1-((8-Quinolinylamino)carbonyl)-1'-pentan-3-ferrocene (2d)**

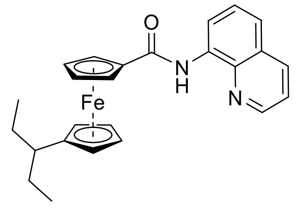

Substrate **2d** was synthesized according to **General Procedure 2**; orange solid.

**$^{1}$H NMR (400 MHz, Chloroform-*d*)** δ 10.27 (s, 1H), 8.87 (dt, *J* = 4.2, 1.4 Hz, 1H), 8.82 (dd, *J* = 7.6, 1.3 Hz, 1H), 8.18 (d, *J* = 8.3, 1.3 Hz, 1H), 7.57 (t, *J* = 7.9 Hz, 1H), 7.53 – 7.49 (m, 1H), 7.49 – 7.43 (m, 1H), 4.89 (q, *J* = 1.6 Hz, 2H), 4.41 (q, *J* = 1.5 Hz, 2H), 4.20 (q, *J* = 1.5 Hz, 2H), 4.11 (q, *J* = 1.5 Hz, 2H), 2.27 (q, *J* = 7.2, 6.3 Hz, 1H), 1.65 – 1.57 (m, 2H), 1.49 (m, *J* = 14.0, 7.2 Hz, 2H), 0.79 (t, *J* = 7.4 Hz, 6H).

**$^{13}$C NMR (100 MHz, $CDCl_3$)** δ 169.3, 148.3, 138.7, 136.5, 135.0, 128.2, 127.7, 121.7, 121.0, 116.2, 97.1, 71.6, 69.1, 69.1, 69.0, 40.3, 26.7, 11.3.

**HRMS (ESI)** m/z: $[M + Na]^+$ Calcd for $C_{25}H_{26}FeN_2ONa$ 449.1287; found: 449.1288.

**1-((8-Quinolinylamino)carbonyl)-1’-2-methyl-1-ene-ferrocene (2e)**

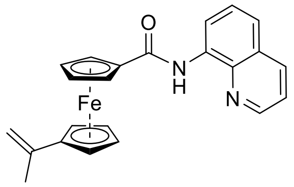


Substrate **2e** was synthesized according to **General Procedure 3**; yellow solid.

**$^1$H NMR (400 MHz, Chloroform-*d*)** δ 10.22 (s, 1H), 8.88 (dd, *J* = 4.2, 1.7 Hz, 1H), 8.83 (dd, *J* = 7.6, 1.4 Hz, 1H), 8.19 (dd, *J* = 8.2, 1.6 Hz, 1H), 7.58 (t, *J* = 7.9 Hz, 1H), 7.53 – 7.46 (m, 2H), 5.10 (s, 1H), 4.88 (t, *J* = 1.9 Hz, 2H), 4.76 (m, *J* = 1.6 Hz, 1H), 4.49 (t, *J* = 1.9 Hz, 2H), 4.40 (t, *J* = 1.8 Hz, 2H), 4.34 (t, *J* = 1.9 Hz, 2H), 2.05 – 1.98 (m, 3H).

**$^{13}$C NMR (100 MHz, $CDCl_3$)** δ 168.9, 148.2, 139.9, 138.6, 136.4, 134.8, 128.1, 127.6, 121.7, 121.0, 116.2, 110.1, 88.2, 72.3, 70.7, 69.7, 67.5, 21.5.

**HRMS (ESI)** m/z: $[M + Na]^+$ Calcd for $C_{23}H_{20}FeN_2ONa$ 419.0817; found:419.0817.

**1-((8-Quinolinylamino)carbonyl)-1’-acetyl-ferrocene (2f)**

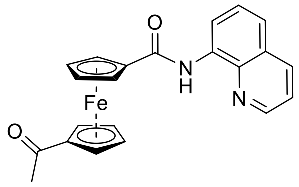


Substrate **2f** was synthesized according to **General Procedure 1**; orange solid.

**$^1$H NMR (400 MHz, Chloroform-*d*)** δ 10.26 (s, 1H), 8.89 (dd, *J* = 4.2, 1.6 Hz, 1H), 8.81 (dd, *J* = 7.4, 1.5 Hz, 1H), 8.20 (dd, *J* = 8.2, 1.6 Hz, 1H), 7.63 – 7.53 (m, 2H), 7.50 (dd, *J* = 8.3, 4.2 Hz, 1H), 4.99 (t, *J* = 1.9 Hz, 2H), 4.86 (t, *J* = 1.9 Hz, 2H), 4.60 (t, *J* = 1.9 Hz, 2H), 4.49 (t, *J* = 1.9 Hz, 2H), 2.39 (s, 3H).

**$^{13}$C NMR (100 MHz, $CDCl_3$)** δ 201.8, 167.6, 148.5, 138.6, 136.6, 134.5, 128.2, 127.7, 121.9, 121.6, 116.5, 80.7, 78.6, 74.2, 72.8, 71.3, 70.1, 27.8.

**HRMS (ESI)** m/z: $[M + H]^+$ Calcd for $C_{22}H_{19}FeN_2O_2$ 399.0718; found: 399.0708.

**1-((8-Quinolinylamino)carbonyl)-1'-butyryl-ferrocene (2g)**

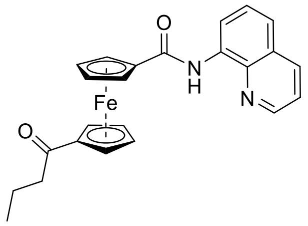

Substrate **2g** was synthesized according to **General Procedure 1**; orange solid.

**$^{1}$H NMR (400 MHz, Chloroform-*d*)** δ 10.26 (s, 1H), 8.89 (dd, *J* = 4.2, 1.7 Hz, 1H), 8.82 (dd, *J* = 7.4, 1.6 Hz, 1H), 8.20 (dd, *J* = 8.3, 1.7 Hz, 1H), 7.62 – 7.53 (m, 2H), 7.50 (dd, *J* = 8.3, 4.2 Hz, 1H), 4.98 (t, *J* = 1.9 Hz, 2H), 4.87 (t, *J* = 1.9 Hz, 2H), 4.58 (t, *J* = 2.0 Hz, 2H), 4.48 (t, *J* = 1.9 Hz, 2H), 2.67 (t, *J* = 7.4 Hz, 2H), 1.64 (d, *J* = 7.3 Hz, 2H), 0.86 (t, *J* = 7.4 Hz, 3H).

**$^{13}$C NMR (100 MHz, CDCl$_3$)** δ 204.1, 167.6, 148.5, 138.6, 136.5, 134.5, 128.2, 127.6, 121.9, 121.5, 116.4, 80.6, 78.5, 74.0, 72.7, 71.0, 70.0, 41.9, 17.8, 14.0.

**HRMS (ESI)** m/z: [M + H]$^+$ Calcd for $C_{24}H_{23}FeN_2O_2$ 427.1031; found:427.1102.

**1-((8-Quinolinylamino)carbonyl)-1'-valeryl-ferrocene (2h)**

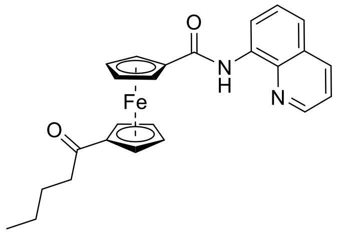

Substrate **2h** was synthesized according to **General Procedure 1**; orange solid.

**$^{1}$H NMR (400 MHz, Chloroform-*d*)** δ 10.25 (s, 1H), 8.88 (dd, *J* = 4.2, 1.7 Hz, 1H), 8.82 (dd, *J* = 7.4, 1.6 Hz, 1H), 8.19 (dd, *J* = 8.3, 1.7 Hz, 1H), 7.61 – 7.52 (m, 2H), 7.49 (dd, *J* = 8.3, 4.2 Hz, 1H), 4.97 (t, *J* = 1.9 Hz, 2H), 4.87 (t, *J* = 2.0 Hz, 2H), 4.58 (t, *J* = 2.0 Hz, 2H), 4.47 (t, *J* = 1.9 Hz, 2H), 2.76 – 2.59 (m, 2H), 1.61 – 1.53 (m, 2H), 1.27 – 1.19 (m, 2H), 0.83 (t, *J* = 7.3 Hz, 3H).

**$^{13}$C NMR (100 MHz, CDCl$_3$)** δ 204.2, 167.6, 148.5, 138.6, 136.5, 134.5, 128.2, 127.6, 121.8, 121.5, 116.4, 80.6, 78.5, 74.0, 72.6, 71.0, 70.0, 39.8, 26.5, 22.6, 14.0.

**HRMS (ESI)** m/z: [M + H]$^+$ Calcd for $C_{25}H_{24}FeN_2O_2$ 441.1187; found: 441.1261.

**1-((8-Quinolinylamino)carbonyl)-1'-2-methyl-1-butyryl-ferrocene (2i)**

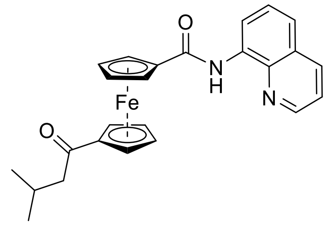

Substrate **2i** was synthesized according to **General Procedure 1**; orange solid.

**$^1$H NMR (400 MHz, Chloroform-*d*)** δ 10.26 (s, 1H), 8.89 (dd, *J* = 4.2, 1.6 Hz, 1H), 8.82 (dd, *J* = 7.4, 1.5 Hz, 1H), 8.20 (dd, *J* = 8.3, 1.6 Hz, 1H), 7.61 – 7.52 (m, 2H), 7.50 (dd, *J* = 8.3, 4.2 Hz, 1H), 4.98 (t, *J* = 1.9 Hz, 2H), 4.86 (t, *J* = 1.9 Hz, 2H), 4.58 (t, *J* = 1.9 Hz, 2H), 4.48 (t, *J* = 1.9 Hz, 2H), 2.56 (d, *J* = 6.9 Hz, 2H), 2.18 (hept, *J* = 6.7 Hz, 1H), 0.89 (d, *J* = 6.6 Hz, 6H).

**$^{13}$C NMR (100 MHz, $CDCl_3$)** δ 203.8, 167.7, 148.5, 138.6, 136.6, 134.6, 128.2, 127.6, 121.9, 121.5, 116.4, 81.0, 78.5, 74.2, 72.7, 71.0, 70.0, 49.0, 25.1, 22.8.

**HRMS (ESI)** m/z: $[M + H]^+$ Calcd for $C_{25}H_{25}FeN_2O_2$ 441.1187; found:441.1259.

**1-((8-Quinolinylamino)carbonyl)-1'-2,2-dimethyl-1-propionyl-ferrocene (2j)**

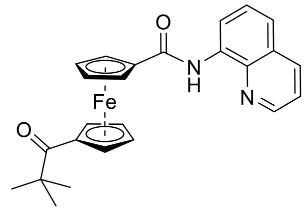

Substrate **2j**was synthesized according to **General Procedure 1**; orange solid.

**$^1$H NMR (400 MHz, Chloroform-*d*)** δ 10.27 (s, 1H), 8.89 (dd, *J* = 4.2, 1.7 Hz, 1H), 8.79 (dd, *J* = 7.5, 1.6 Hz, 1H), 8.20 (dd, *J* = 8.2, 1.7 Hz, 1H), 7.61 – 7.52 (m, 2H), 7.50 (dd, *J* = 8.3, 4.2 Hz, 1H), 4.97 (dt, *J* = 3.9, 1.9 Hz, 4H), 4.55 (t, *J* = 2.0 Hz, 2H), 4.46 (t, *J* = 2.0 Hz, 2H), 1.31 (s, 9H).

**$^{13}$C NMR (100 MHz, $CDCl_3$)** δ 209.8, 167.9, 148.5, 138.6, 136.5, 134.7, 128.2, 127.6, 121.8, 121.4, 116.4, 78.3, 78.3, 73.7, 73.3, 72.5, 69.9, 44.6, 28.1.

**HRMS (ESI)** m/z: $[M + H]^+$ Calcd for $C_{25}H_{24}FeN_2O_2$ 441.1187; found: 441.1261.

## 1-((8-Quinolinylamino)carbonyl)-1'-(*o*-tolyl)acetyl-ferrocene (2k)

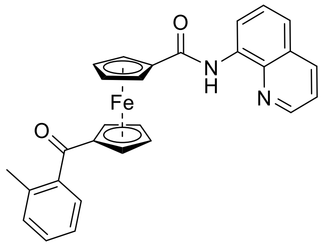


Substrate **2k** was synthesized according to **General Procedure 1**; orange solid.

**$^1$H NMR (400 MHz, Chloroform-*d*)** δ 10.24 (s, 1H), 8.85 (dd, *J* = 4.2, 1.7 Hz, 1H), 8.74 (dd, *J* = 7.1, 1.9 Hz, 1H), 8.19 (dd, *J* = 8.3, 1.7 Hz, 1H), 7.60 – 7.51 (m, 3H), 7.49 (dd, *J* = 8.3, 4.2 Hz, 1H), 7.33 – 7.27 (m, 1H), 7.21 – 7.11 (m, 2H), 5.02 (t, *J* = 1.9 Hz, 2H), 4.89 (t, *J* = 2.0 Hz, 2H), 4.65 (t, *J* = 1.9 Hz, 2H), 4.55 (t, *J* = 1.9 Hz, 2H), 2.40 (s, 3H).

**$^{13}$C NMR (100 MHz, $CDCl_3$)** δ 201.6, 167.6, 148.5, 139.6, 138.6, 136.5, 136.1, 134.6, 131.2, 130.1, 128.1, 127.8, 127.6, 125.2, 121.8, 121.5, 116.5, 80.7, 78.6, 74.9, 73.1, 72.8, 70.1, 20.1.

**HRMS (ESI)** m/z: $[M + H]^+$ Calcd for $C_{28}H_{23}FeN_2O_2$ 475.1031; found: 475.1104.

## 1-((8-Quinolinylamino)carbonyl)-1'-*p*-chlorophenylacetyl -ferrocene (2l)

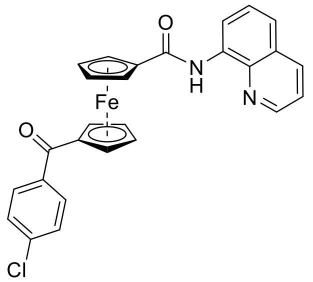


Substrate **2l** was synthesized according to **General Procedure 1**; orange solid.

**$^1$H NMR (400 MHz, Chloroform-*d*)** δ 10.16 (s, 1H), 8.85 (dd, *J* = 4.2, 1.7 Hz, 1H), 8.70 (dd, *J* = 6.7, 2.2 Hz, 1H), 8.20 (dd, *J* = 8.3, 1.7 Hz, 1H), 7.73 (d, *J* = 8.5 Hz, 2H), 7.59 – 7.52 (m, 2H), 7.50 (dd, *J* = 8.3, 4.2 Hz, 1H), 7.17 (d, *J* = 8.5 Hz, 2H), 5.02 (t, *J* = 2.0 Hz, 2H), 4.99 – 4.94 (m, 2H), 4.73 – 4.63 (m, 2H), 4.53 – 4.43 (m, 2H).

**$^{13}$C NMR (100 MHz, $CDCl_3$)** δ 196.9, 167.3, 148.4, 138.5, 138.1, 137.4, 136.6, 134.4, 129.7, 128.5, 128.1, 127.6, 121.8, 121.6, 116.4, 79.4, 78.9, 74.6, 73.1, 70.6.

**HRMS (ESI)** m/z: $[M + H]^+$ Calcd for $C_{27}H_{20}ClFeN_2O_2$ 495.0484; found:495.0560.

## 1-((8-Quinolinylamino)carbonyl)- 1’-methanol-ferrocene (2m)

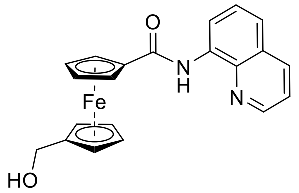


Substrate **2m** was synthesized according to **General Procedure 5**; yellow solid.

**$^{1}$H NMR (400 MHz, Chloroform-*d*)** δ 10.28 (s, 1H), 8.89 – 8.84 (m, 2H), 8.20 (dd, *J* = 8.2, 1.7 Hz, 1H), 7.61 – 7.52 (m, 2H), 7.49 (dd, *J* = 8.2, 4.2 Hz, 1H), 4.97 (t, *J* = 1.9 Hz, 2H), 4.50 (t, *J* = 1.9 Hz, 2H), 4.38 (s, 2H), 4.32 (t, *J* = 1.9 Hz, 2H), 4.23 (t, *J* = 1.9 Hz, 2H).

**$^{13}$C NMR (100 MHz, $CDCl_3$)** δ 170.3, 148.5, 138.6, 136.6, 134.4, 128.2, 127.7, 121.8, 121.7, 116.9, 91.9, 71.4, 69.2, 69.2, 68.7, 60.1.

**HRMS (ESI)** m/z: $[M + Na]^+$ Calcd for $C_{21}H_{18}FeN_2O_2Na$ 409.0615; found:409.0613.

# 4. Screening of reaction conditions

**Table S1.** Screening of solvents [a]

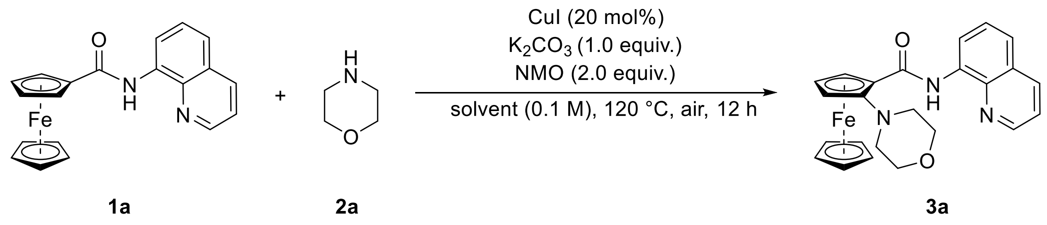


| entry | solvents (0.1 M) | yield (%)[b] |
|---|---|---|
| 1 | DMF | 11 |
| 2 | DMSO | trace |
| 3 | NMP | 15 |
| 4 | MeCN | 32 |
| 5 | iPrOH | trace |
| 6 | HFIP | n.r |

[a] Reactions conditions: **1a** (0.1 mmol), **2a** (0.3 mmo), CuI (20 mol%), $K_2CO_3$ (1.0 equiv.), NMO (2.0 equiv.), solvent (0.1 M) under air at 120 °C for 12 h. [b] Isolated yield.

**Table S2.** Screening of catalysts [a]

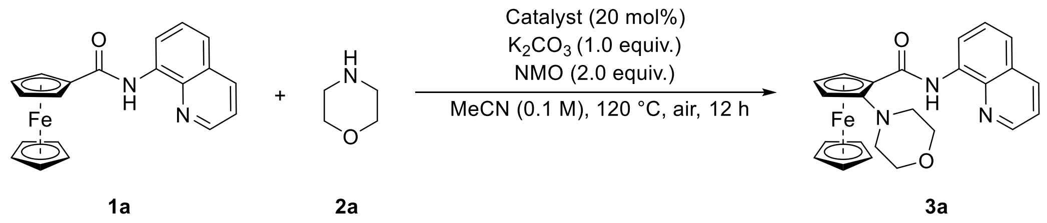


| entry | catalyst (20 mol%) | yield (%)[b] |
|---|---|---|
| 1 | $Cu(OAc)_2$ | 10 |
| 2 | CuCN | 12 |
| 3 | $Cu_2O$ | n.r |
| 4 | CuCl | 18 |
| 5 | CuI | 32 |
| 6 | TcCu | trace |

[a] Reactions conditions: **1a** (0.1 mmol), **2a** (0.3 mmo), Catalyst (20 mol%), $K_2CO_3$ (1.0 equiv.), NMO (2.0 equiv.), MeCN (0.1 M) under air at 120 °C for 12 h. [b] Isolated yield.

**Table S3.** Screening of several bases [a]

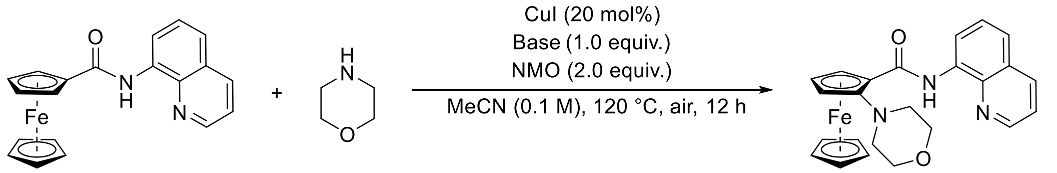


| 1a | 2a | 3a |
|---|---|---|

| entry | base (1.0 equiv.) | yield (%)[b] |
|---|---|---|
| 1 | $K_3PO_4$ | 28 |
| 2 | $Cs_2CO_3$ | trace |
| 3 | $K_2CO_3$ | 32 |
| 4 | DMAP | 29 |

[a] Reactions conditions: **1a** (0.1 mmol), **2a** (0.3 mmo), CuI (20 mol%), Base (1.0 equiv.), NMO (2.0 equiv.), MeCN (0.1 M) under air at 120 °C for 12 h. [b] Isolated yield.

**Table S4.** Screening of several oxidants [a]

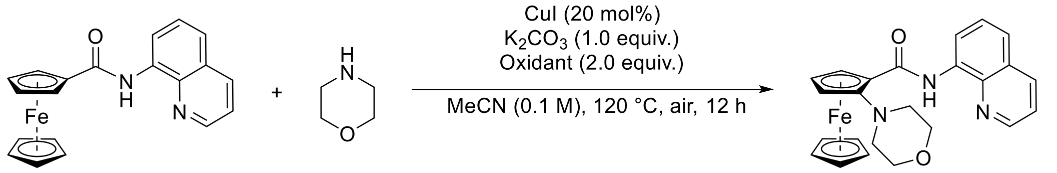


| 1a | 2a | 3a |
|---|---|---|

| entry | oxidant (2.0 equiv.) | yield (%)[b] |
|---|---|---|
| 1 | NMO | 32 |
| 2 | $MnO_2$ | trace |
| 3 | TEMPO | 23 |
| 4 | $O_2$ | 8 |

[a] Reactions conditions: **1a** (0.1 mmol), **2a** (0.3 mmo), CuI (20 mol%), $K_2CO_3$ (1.0 equiv.), Oxidant (2.0 equiv.), MeCN (0.1 M) under air at 120 °C for 12 h. [b] Isolated yield.

**Table S5.** Screening of several additives [a]

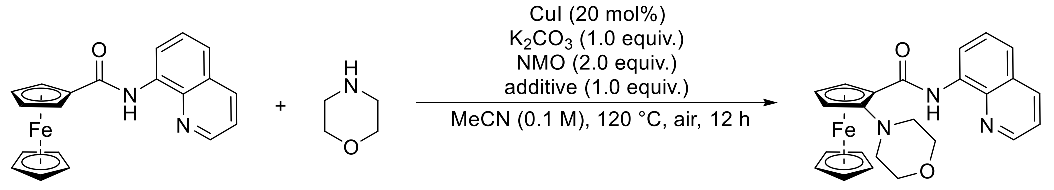


**1a** **2a** **3a**

| entry | additive (1.0 equiv.) | yield (%)[b] |
|---|---|---|
| 1 | 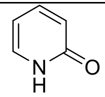 | 34 |
| 2 | 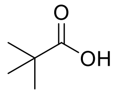 | n.r |
| 3 | 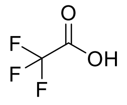 | 33 |
| 4 | 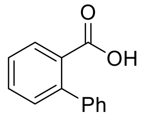 | trace |
| 5 | 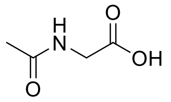 | n.r |
| 6 | 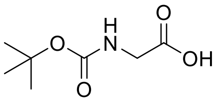 | 23 |

[a] Reactions conditions: **1a** (0.1 mmol), **2a** (0.3 mmo), CuI (20 mol%), $K_2CO_3$ (1.0 equiv.), NMO (2.0 equiv.), additive (1.0 equiv.), MeCN (0.1 M) under air at 120 °C for 12 h. [b] Isolated yield.

**Table S6.** Screening of several solvent volumes [a]

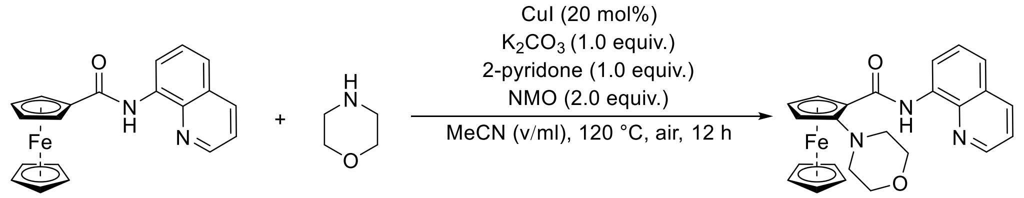


**1a** **2a** **3a**

| entry | MeCN (v/ml) | yield (%)[b] |
|---|---|---|
| 1 | 1.5 | 21 |
| 2 | 1.0 | 34 |
| 3 | 0.8 | 33 |
| 4 | 0.4 | 33 |
| 5 | 0.1 | 34 |
| 6 | neat | 36[c] |

[a] Reactions conditions: **1a** (0.1 mmol), **2a** (0.3 mmo), CuI (20 mol%), $K_2CO_3$ (1.0 equiv.), NMO (2.0 equiv.), 2-pyridone (1.0 equiv.) MeCN (v/ml), under air at 120 °C for 12 h. [b] Isolated yield, [c] Morphine (0.5 mmol).

**Table S7.** Screening of several temperatures [a]

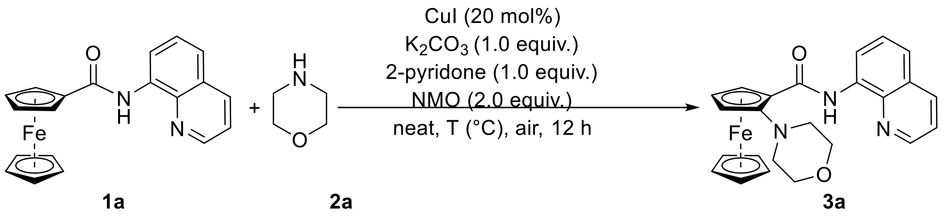


| entry | T (°C) | yield (%)[b] |
|---|---|---|
| 1 | 140 | 10 |
| 2 | 120 | 34 |
| 3 | 100 | 46 |
| 4 | 80 | 56 |
| 5 | 60 | 43 |

[a] Reactions conditions: **1a** (0.1 mmol), **2a** (0.5 mmo), CuI (20 mol%), $K_2CO_3$ (1.0 equiv.), NMO (2.0 equiv.), 2-pyridone (1.0 equiv) under air for 12 h. [b] Isolated yield.

**Table S8.** Screening of several times [a]

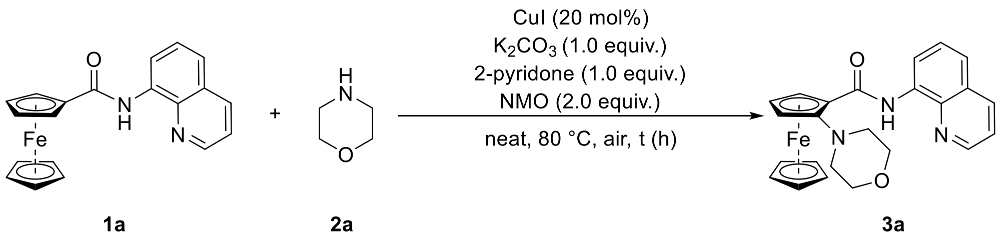


| entry | t (h) | yield (%)[b] |
|---|---|---|
| 1 | 12 | 56 |
| 2 | 10 | 57 |
| 3 | 8 | 59 |
| 4 | 6 | 68 |
| 5 | 4 | 80 |
| 6 | 2 | 52 |

[a] Reactions conditions: **1a** (0.1 mmol), **2a** (0.5 mmo), CuI (20 mol%), $K_2CO_3$ (1.0 equiv.), NMO (2.0 equiv.), 2-pyridone (1.0 equiv.) under air at 80 °C. [b] Isolated yield.

## 5. General procedures for Cu-catalyzed monoselective C–H amination of ferrocenes with alkylamines

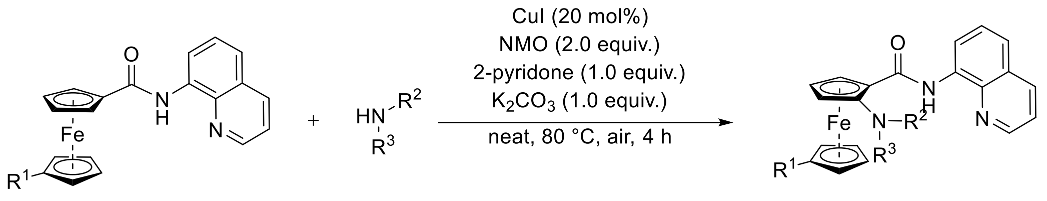


To a 10 mL Schlenk tube was added **1** (0.1 mmol), **2** (0.5 mmol), $K_2CO_3$ (14.0 mg, 1.0 equiv), CuI (3.8 mg, 20 mol %), NMO (23.4 mg, 2.0 equiv) and 2-pyridone (9.5 mg, 1.0 equiv), stirred at 80 °C (aluminum heat transfer block) for 4 h. After cooling to room temperature, the mixture was diluted with DCM, the resulting residue was purified by preparative TLC using Hexane/EtOAc as the eluent to afford the desired product.

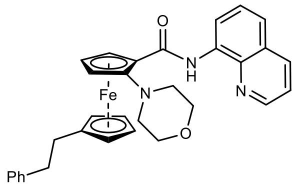

**1-Morpholino-2-((8-quinolinylamino)carbonyl)-1'-phenethyl-ferrocene (3b)**

A purification by flash chromatography in petroleum ether : ethyl acetate= 4 : 1 to give **3b** as yellow foam (33.8 mg, 62%).

**$^1$H NMR (400 MHz, Chloroform-*d*)** δ 12.27 (s, 1H), 9.02 (dd, *J* = 7.5, 1.6 Hz, 1H), 8.87 (dd, *J* = 4.2, 1.7 Hz, 1H), 8.19 (dd, *J* = 8.2, 1.7 Hz, 1H), 7.59 – 7.52 (m, 2H), 7.52 – 7.45 (m, 1H), 7.11 (m *J* = 4.6, 1.6 Hz, 3H), 6.88 (dd, *J* = 7.2, 2.4 Hz, 2H), 4.88 (dd, *J* = 2.8, 1.5 Hz, 1H), 4.30 (dd, *J* = 2.7, 1.6 Hz, 1H), 4.26 (t, *J* = 2.8 Hz, 1H), 4.14 – 3.93 (m, 8H), 3.08 (s, 2H), 2.94 (m, *J* = 11.6, 5.9, 3.4 Hz, 2H), 2.65 – 2.44 (m, 4H).

**$^{13}$C NMR (100 MHz, $CDCl_3$)** δ 169.3, 148.1, 141.7, 139.0, 136.3, 136.3, 128.4, 128.3, 128.2, 127.7, 125.8, 121.6, 121.2, 117.6, 112.0, 90.5, 72.7, 71.4, 70.2, 70.1, 69.6, 67.8, 67.2, 66.6, 60.0, 55.5, 37.6, 29.9.

**HRMS (ESI)** m/z: $[M + Na]^+$ Calcd for $C_{32}H_{31}FeN_3O_2Na$ 568.1658; found: 568.1661.

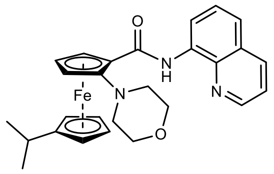

**1-Morpholino-2-((8-quinolinylamino)carbonyl)-1'-isopropyl-ferrocene (3c)**

A purification by flash chromatography in petroleum ether : ethyl acetate= 4 : 1 to give **3c** as yellow foam (32.8 mg, 68%).

**$^{1}$H NMR (400 MHz, Chloroform-*d*)** δ 12.27 (s, 1H), 9.01 (dd, *J* = 7.6, 1.4 Hz, 1H), 8.95 (dd, *J* = 4.2, 1.7 Hz, 1H), 8.19 (dd, *J* = 8.3, 1.7 Hz, 1H), 7.58 (t, *J* = 7.9 Hz, 1H), 7.55 – 7.46 (m, 2H), 4.87 (dd, *J* = 2.8, 1.5 Hz, 1H), 4.33 (s, 1H), 4.27 (t, *J* = 2.8 Hz, 1H), 4.16 – 4.07 (m, 4H), 4.07 – 3.99 (m, 4H), 3.03 (d, *J* = 50.7 Hz, 4H), 2.60 (m, *J* = 6.8 Hz, 1H), 1.13 (d, *J* = 6.8 Hz, 3H), 1.08 (d, *J* = 6.9 Hz, 3H).

**$^{13}$C NMR (100 MHz, CDCl$_3$)** δ 169.6, 148.2, 139.1, 136.4, 136.3, 128.4, 127.7, 121.6, 121.1, 117.5, 111.8, 98.7, 72.2, 71.0, 70.0, 70.0, 68.1, 67.5, 67.4, 66.6, 60.0, 55.5, 27.2, 23.9, 23.5.

**HRMS (ESI)** m/z: [M + Na]$^+$ Calcd for $C_{27}H_{29}FeN_3O_2Na$ 506.1501; found: 506.1497.

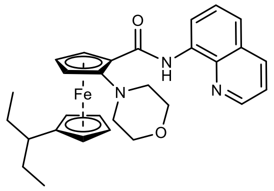

**1-Morpholino-2-((8-quinolinylamino)carbonyl)-1'-pentan-3-ferrocene (3d)**

A purification by flash chromatography in petroleum ether : ethyl acetate= 4 : 1 to give **3d** as yellow foam (29.6mg, 58%).

**$^{1}$H NMR (400 MHz, Chloroform-*d*)** δ 12.28 (s, 1H), 9.02 (dd, *J* = 7.7, 1.5 Hz, 1H), 8.95 (dd, *J* = 4.2, 1.7 Hz, 1H), 8.19 (dd, *J* = 8.2, 1.7 Hz, 1H), 7.58 (t, *J* = 7.9 Hz, 1H), 7.55 – 7.46 (m, 2H), 4.85 (dd, *J* = 2.8, 1.5 Hz, 1H), 4.32 (dd, *J* = 2.7, 1.6 Hz, 1H), 4.23 (t, *J* = 2.7 Hz, 1H), 4.15 – 4.09 (m, 2H), 4.09 (dt, *J* = 3.8, 1.3 Hz, 3H), 4.03 (dd, *J* = 6.0, 3.4 Hz, 5H), 3.09 (s, 2H), 3.00 – 2.89 (m, 2H), 2.18 (tt, *J* = 7.4, 5.2 Hz, 1H), 1.70 – 1.56 (m, 1H), 1.56 – 1.33 (m, 3H), 0.79 (t, *J* = 7.4 Hz, 3H), 0.68 (t, *J* = 7.4 Hz, 3H).

**$^{13}$C NMR (100 MHz, CDCl$_3$)** δ 169.6, 148.2, 139.1, 136.4, 128.4, 127.7, 121.6, 121.1, 117.5, 111.8, 97.0, 72.3, 70.8, 70.7, 69.8, 68.3, 68.0, 67.6, 66.7, 60.1, 39.9, 26.5, 26.3, 11.3, 11.0.

**HRMS (ESI)** m/z: $[M + Na]^+$ Calcd for $C_{29}H_{33}FeN_3O_2Na$ 534.1814; found: 534.1815.

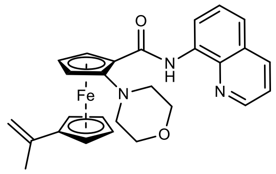

**1-Morpholino-2-((8-quinolinylamino)carbonyl)-1'-2-methyl-1-ene-ferrocene (3e)**

A purification by flash chromatography in petroleum ether : ethyl acetate= 4 : 1 to give **3e** as yellow foam (26.5 mg, 55%).

$^1$H NMR (400 MHz, Chloroform-*d*) δ 12.21 (s, 1H), 9.02 (dd, *J* = 7.6, 1.5 Hz, 1H), 8.96 (dd, *J* = 4.2, 1.7 Hz, 1H), 8.19 (dd, *J* = 8.3, 1.7 Hz, 1H), 7.58 (t, *J* = 7.9 Hz, 1H), 7.55 – 7.47 (m, 2H), 5.04 (s, 1H), 4.86 (dd, *J* = 2.8, 1.5 Hz, 1H), 4.76 – 4.71 (m, 1H), 4.45 (q, *J* = 1.8 Hz, 1H), 4.41 (q, *J* = 1.7 Hz, 1H), 4.29 (dd, *J* = 2.7, 1.6 Hz, 1H), 4.25 (t, *J* = 1.9 Hz, 2H), 4.21 (t, *J* = 2.7 Hz, 1H), 4.05 (m, *J* = 9.8, 6.1, 3.1 Hz, 4H), 3.09 (s, 2H), 2.93 (m, *J* = 11.7, 5.9, 3.4 Hz, 2H), 1.95 (s, 3H).

**$^{13}$C NMR (100 MHz, $CDCl_3$)** δ 168.9, 148.1, 139.7, 139.1, 136.4, 128.4, 127.7, 121.6, 121.1, 117.5, 112.3, 110.4, 88.6, 73.0, 72.0, 71.5, 68.6, 68.5, 68.2, 67.4, 66.6, 60.5, 55.4, 52.8, 21.6.

**HRMS (ESI)** m/z: $[M]^+$ Calcd for $C_{27}H_{27}FeN_3O_2$ 481.1453; found: 481.1449.

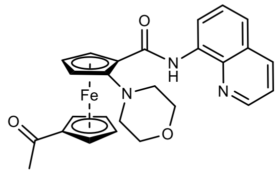

**1-Morpholino-2-((8-quinolinylamino)carbonyl)-1'-acetyl-ferrocene (3f)**

A purification by flash chromatography in petroleum ether : ethyl acetate= 4 : 1 to give **3f** as yellow foam (31.9mg, 66%).

**$^1$H NMR (400 MHz, Chloroform-*d*)** δ 12.18 (s, 1H), 9.00 – 8.94 (m, 2H), 8.20 (dd, *J* = 8.3, 1.7 Hz, 1H), 7.61 – 7.55 (m, 2H), 7.54 – 7.49 (m, 1H), 4.97 (dd, *J* = 2.8, 1.6 Hz, 1H), 4.86 (dt, *J* = 2.7, 1.3 Hz, 1H), 4.75 (dt, *J* = 2.6, 1.3 Hz, 1H), 4.50 (m, *J* = 4.5, 2.6, 1.3 Hz, 2H), 4.35 (dd, *J* = 2.8, 1.6 Hz, 1H), 4.32 (t, *J* = 2.8 Hz, 1H), 4.02 (m, *J* = 14.3, 6.4, 3.1 Hz, 4H), 3.04 (s, 2H), 2.90 (m, *J* = 11.6, 6.1, 3.1 Hz, 2H), 2.31 (s, 3H).

**$^{13}$C NMR (100 MHz, CDCl$_3$)** δ 201.8, 168.1, 148.4, 139.0, 136.5, 135.8, 128.4, 127.7, 121.8, 121.6, 117.6, 113.5, 80.8, 75.5, 75.1, 73.0, 72.2, 71.4, 68.9, 67.9, 66.5, 61.0, 55.1, 27.9.

**HRMS (ESI)** m/z: [M + Na]$^+$ Calcd for $C_{26}H_{25}FeN_3O_3Na$ 506.1137; found: 506.1142.

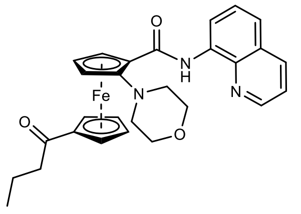


**1-Morpholino-2-((8-quinolinylamino)carbonyl)-1'-butyryl-ferrocene (3g)**

A purification by flash chromatography in petroleum ether : ethyl acetate= 4 : 1 to give **3g** as yellow foam (17.9 mg, 35%).

**$^1$H NMR (400 MHz, Chloroform-*d*)** δ 12.19 (s, 1H), 9.02 – 8.95 (m, 2H), 8.21 (dd, *J* = 8.2, 1.7 Hz, 1H), 7.62 – 7.55 (m, 2H), 7.52 (dd, *J* = 8.2, 4.1 Hz, 1H), 4.98 (dd, *J* = 2.8, 1.5 Hz, 1H), 4.89 (dt, *J* = 2.7, 1.3 Hz, 1H), 4.78 (dt, *J* = 2.6, 1.3 Hz, 1H), 4.48 (m, *J* = 2.6, 1.2 Hz, 2H), 4.35 (dd, *J* = 2.8, 1.6 Hz, 1H), 4.31 (t, *J* = 2.8 Hz, 1H), 4.10 – 3.92 (m, 4H), 3.06 (s, 2H), 2.90 (m, *J* = 11.6, 6.2, 3.0 Hz, 2H), 2.70 – 2.50 (m, 2H), 1.64 – 1.49 (m, 2H), 0.84 (t, *J* = 7.4 Hz, 3H).

**$^{13}$C NMR (100 MHz, CDCl$_3$)** δ 204.1, 168.2, 148.4, 139.0, 136.5, 135.9, 128.4, 127.7, 121.8, 121.6, 117.7, 113.4, 80.7, 75.5, 74.9, 73.0, 71.5, 71.3, 68.7, 67.9, 66.6, 61.0, 55.2, 42.0, 17.8, 13.9.

**HRMS (ESI)** m/z: [M + Na]$^+$ Calcd for $C_{28}H_{29}FeN_3O_3Na$ 534.1450; found: 534.1455.

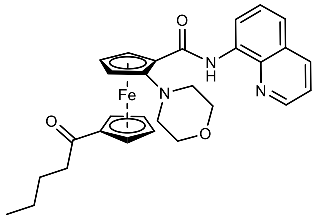


**1-Morpholino-2-((8-quinolinylamino)carbonyl)-1'-valeryl-ferrocene (3h)**

A purification by flash chromatography in petroleum ether : ethyl acetate= 4 : 1 to give **3h** as yellow foam (27.8mg, 53%).

**$^1$H NMR (400 MHz, Chloroform-*d*)** δ 12.18 (s, 1H), 9.01 – 8.95 (m, 2H), 8.21 (dd, *J* = 8.3, 1.7 Hz, 1H), 7.62 – 7.55 (m, 2H), 7.52 (dd, *J* = 8.2, 4.1 Hz, 1H), 4.98 (dd, *J* = 2.8, 1.6 Hz, 1H), 4.88 (dt, *J* = 2.7, 1.4 Hz, 1H), 4.78 (dt, *J* = 2.6, 1.3 Hz, 1H), 4.48 (pd, *J* = 2.5, 1.3 Hz, 2H), 4.34 (dd, *J* = 2.8,

1.6 Hz, 1H), 4.30 (t, $J$ = 2.8 Hz, 1H), 4.03 (m, $J$ = 16.8, 8.1, 5.0 Hz, 4H), 3.06 (s, 2H), 2.90 (m, $J$ = 11.5, 6.2, 3.0 Hz, 2H), 2.70 – 2.52 (m, 2H), 1.62 – 1.42 (m, 2H), 1.28 – 1.13 (m, 2H), 0.84 (t, $J$ = 7.3 Hz, 3H).

**$^{13}$C NMR (100 MHz, $CDCl_3$)** δ 204.3, 168.2, 148.4, 139.0, 136.5, 135.9, 128.4, 127.7, 121.8, 121.6, 117.7, 113.4, 80.7, 75.5, 74.9, 73.0, 71.6, 71.3, 68.7, 67.8, 66.6, 61.0, 55.2, 39.9, 26.6, 22.5, 14.0.

**HRMS (ESI)** m/z: $[M + Na]^+$ Calcd for $C_{29}H_{31}FeN_3O_3Na$ 548.1607; found: 548.1608.

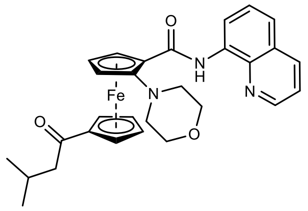


**1-Morpholino-2-((8-quinolinylamino)carbonyl)-1'-2-methyl-1-butyryl-ferrocene (3i)**

A purification by flash chromatography in petroleum ether : ethyl acetate= 4 : 1 to give **3i** as yellow foam (24.2 mg, 46%).

**$^1$H NMR (400 MHz, Chloroform-*d*)** δ 12.19 (s, 1H), 9.03 – 8.91 (m, 2H), 8.21 (dd, $J$ = 8.3, 1.7 Hz, 1H), 7.62 – 7.55 (m, 2H), 7.52 (dd, $J$ = 8.3, 4.2 Hz, 1H), 4.98 (dd, $J$ = 2.8, 1.6 Hz, 1H), 4.89 (dt, $J$ = 2.6, 1.4 Hz, 1H), 4.76 (dt, $J$ = 2.7, 1.4 Hz, 1H), 4.47 (dt, $J$ = 2.6, 1.3 Hz, 2H), 4.34 (dd, $J$ = 2.8, 1.6 Hz, 1H), 4.31 (t, $J$ = 2.8 Hz, 1H), 4.10 – 3.92 (m, 4H), 3.06 (s, 2H), 2.90 (m $J$ = 11.6, 6.2, 3.0 Hz, 2H), 2.58 – 2.41 (m, 2H), 2.13 (dt, $J$ = 13.5, 6.7 Hz, 1H), 0.90 (d, $J$ = 6.6 Hz, 3H), 0.84 (d, $J$ = 6.7 Hz, 3H).

**$^{13}$C NMR (100 MHz, $CDCl_3$)** δ 203.9, 168.2, 148.4, 139.0, 136.5, 135.9, 128.4, 127.7, 121.8, 121.6, 117.6, 113.4, 80.9, 75.8, 75.0, 73.0, 71.5, 71.4, 68.6, 67.9, 66.6, 61.0, 55.1, 49.0, 25.2, 22.8.

**HRMS (ESI)** m/z: $[M + Na]^+$ Calcd for $C_{29}H_{31}FeN_3O_3Na$ 548.1607; found: 548.1609.

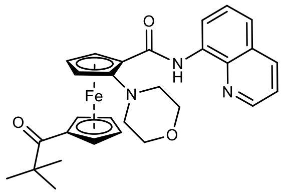


**1-Morpholino-2-((8-quinolinylamino)carbonyl)-1'-2,2-dimethyl-1-propionyl-ferrocene (3j)**

A purification by flash chromatography in petroleum ether : ethyl acetate= 4 : 1 to give **3j** as yellow

foam (33.6 mg, 64%).

**$^{1}$H NMR (400 MHz, Chloroform-*d*)** δ 12.20 (s, 1H), 8.99 – 8.91 (m, 2H), 8.20 (dd, *J* = 8.2, 1.7 Hz, 1H), 7.61 – 7.54 (m, 2H), 7.52 (dd, *J* = 8.3, 4.2 Hz, 1H), 5.02 (dt, *J* = 2.7, 1.4 Hz, 1H), 4.99 (dd, *J* = 2.9, 1.5 Hz, 1H), 4.84 (dt, *J* = 2.6, 1.3 Hz, 1H), 4.42 (m, *J* = 3.8, 2.6, 1.4 Hz, 2H), 4.32 (dd, *J* = 2.7, 1.6 Hz, 1H), 4.27 (t, *J* = 2.8 Hz, 1H), 4.07 – 3.93 (m, 4H), 3.11 (s, 2H), 2.91 (m, *J* = 11.6, 6.0, 3.2 Hz, 2H), 1.29 (s, 9H).

**$^{13}$C NMR (100 MHz, $CDCl_3$)** δ 209.8, 168.4, 148.4, 139.0, 136.5, 135.9, 128.4, 127.7, 121.8, 121.5, 117.6, 113.4, 77.7, 75.3, 75.3, 73.9, 72.6, 71.9, 68.8, 68.5, 66.6, 61.4, 55.1, 44.5, 28.2.

**HRMS (ESI)** m/z: $[M + Na]^+$ Calcd for $C_{29}H_{31}FeN_3O_3Na$ 548.1607; found: 548.1606.

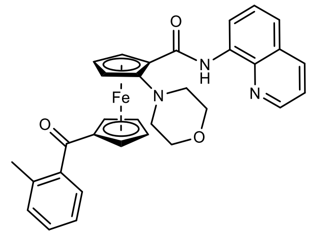


**1-Morpholino-2-((8-quinolinylamino)carbonyl)-1'-(*o*-tolyl)acetyl-ferrocene (3k)**

A purification by flash chromatography in petroleum ether : ethyl acetate= 4 : 1 to give **3k** as yellow foam (33.5 mg, 60%).

**$^{1}$H NMR (400 MHz, Chloroform-*d*)** δ 12.18 (s, 1H), 8.95 (dd, *J* = 4.2, 1.7 Hz, 1H), 8.93 (dd, *J* = 7.2, 1.9 Hz, 1H), 8.21 (dd, *J* = 8.3, 1.7 Hz, 1H), 7.61 – 7.55 (m, 2H), 7.54 – 7.48 (m, 2H), 7.36 (td, *J* = 7.5, 1.5 Hz, 1H), 7.27 (s, 1H), 7.25 – 7.20 (m, 1H), 5.00 (dq, *J* = 2.5, 1.4 Hz, 2H), 4.73 (dt, *J* = 2.6, 1.3 Hz, 1H), 4.54 (td, *J* = 2.6, 1.3 Hz, 1H), 4.47 (td, *J* = 2.5, 1.2 Hz, 1H), 4.43 – 4.38 (m, 2H), 3.97 (dt, *J* = 6.2, 2.9 Hz, 4H), 3.06 (d, *J* = 8.0 Hz, 2H), 2.89 (dt, *J* = 11.6, 4.5 Hz, 2H), 2.40 (s, 3H).

**$^{13}$C NMR (100 MHz, $CDCl_3$)** δ 201.4, 168.1, 148.4, 139.5, 139.0, 136.5, 136.2, 135.8, 131.3, 130.3, 128.4, 127.8, 127.7, 125.2, 121.8, 121.6, 117.7, 113.7, 80.3, 77.5, 75.8, 73.3, 73.0, 72.8, 68.7, 68.4, 66.5, 61.4, 55.1, 20.1.

**HRMS (ESI)** m/z: $[M + Na]^+$ Calcd for $C_{32}H_{29}FeN_3O_3Na$ 582.1450; found: 582.1452.

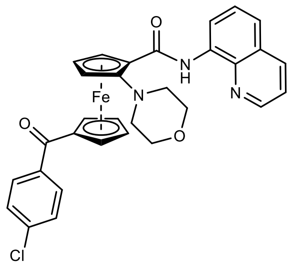

**1-Morpholino-2-((8-quinolinylamino)carbonyl)-1'-*p*-chlorophenylacetyl-ferrocene (3l)**

A purification by flash chromatography in petroleum ether : ethyl acetate= 4 : 1 to give **3l** as yellow foam (26.1 mg, 45%)

**$^1$H NMR (400 MHz, Chloroform-*d*)** δ 12.11 (s, 1H), 8.93 (dd, *J* = 4.2, 1.7 Hz, 1H), 8.89 (dd, *J* = 7.0, 2.1 Hz, 1H), 8.21 (dd, *J* = 8.2, 1.7 Hz, 1H), 7.73 (d, *J* = 8.5 Hz, 2H), 7.62 – 7.55 (m, 2H), 7.52 (dd, *J* = 8.2, 4.2 Hz, 1H), 7.28 (d, *J* = 8.5 Hz, 2H), 5.08 (dt, *J* = 2.5, 1.3 Hz, 1H), 4.99 (dd, *J* = 2.7, 1.7 Hz, 1H), 4.89 (dt, *J* = 2.7, 1.3 Hz, 1H), 4.59 (td, *J* = 2.6, 1.3 Hz, 1H), 4.56 (td, *J* = 2.6, 1.2 Hz, 1H), 4.31 (p, *J* = 2.7 Hz, 2H), 4.03 – 3.90 (m, 4H), 3.04 (s, 2H), 2.88 (ddd, *J* = 11.5, 5.9, 3.4 Hz, 2H).

**$^{13}$C NMR (100 MHz, $CDCl_3$)** δ 196.8, 167.8, 148.3, 139.0, 138.2, 137.5, 136.5, 135.8, 129.8, 128.6, 128.4, 127.7, 121.8, 121.7, 117.7, 113.8, 79.3, 76.3, 75.7, 73.5, 73.4, 73.2, 69.0, 68.7, 66.5, 61.6, 55.0.

**HRMS (ESI)** m/z: $[M + Na]^+$ Calcd for $C_{31}H_{26}ClFeN_3O_3Na$ 602.0904; found: 602.0908.

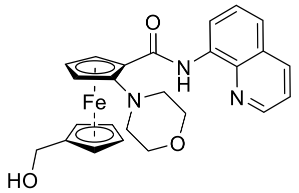

**1-Morpholino-2-((8-quinolinylamino)carbonyl)-1'-methanol-ferrocene (3m)**

A purification by flash chromatography in petroleum ether : ethyl acetate= 4 : 1 to give **3m** as yellow foam (34.4 mg, 73%)

**$^1$H NMR (400 MHz, Chloroform-*d*)** δ 12.28 (s, 1H), 9.02 (d, *J* = 7.3 Hz, 1H), 8.95 (d, *J* = 4.2 Hz, 1H), 8.21 (d, *J* = 8.2 Hz, 1H), 7.62 – 7.48 (m, 3H), 4.99 (d, *J* = 2.6 Hz, 1H), 4.39 (d, *J* = 13.4 Hz, 3H), 4.30 – 4.24 (m, 2H), 4.21 (d, *J* = 5.6 Hz, 2H), 4.17 (d, *J* = 3.2 Hz, 1H), 4.14 – 3.96 (m, 5H), 3.11 (t, *J* = 8.8 Hz, 2H), 3.01 – 2.92 (m, 2H).

**$^{13}$C NMR (101 MHz, CDCl$_3$)** δ 170.7, 148.3, 139.1, 136.6, 135.8, 128.5, 127.8, 121.8, 118.2, 112.0, 92.1, 71.4, 70.2, 70.0, 69.2, 68.3, 67.0, 66.6, 61.2, 59.6, 55.7, 14.3.

**HRMS (ESI)** m/z: [M + Na]$^+$ Calcd for $C_{25}H_{25}FeN_3O_3Na$ 494.1143; found: 494.1136.

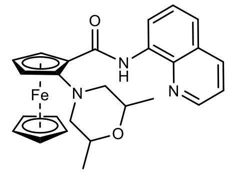


**1-(2,6-Dimethylmorpholino)-2-((8-quinolinylamino)carbonyl)ferrocene (4a)**

A purification by flash chromatography in petroleum ether : ethyl acetate= 4 : 1 to give **4a** as yellow foam (25.3mg, 54%).

**$^1$H NMR (400 MHz, Chloroform-*d*)** δ 12.30 (s, 1H), 9.00 (dd, $J$ = 7.7, 1.5 Hz, 1H), 8.89 (dd, $J$ = 4.2, 1.7 Hz, 1H), 8.19 (dd, $J$ = 8.3, 1.8 Hz, 1H), 7.59 (t, $J$ = 7.9 Hz, 1H), 7.56 – 7.46 (m, 2H), 4.94 (t, $J$ = 2.2 Hz, 1H), 4.46 (m, $J$ = 12.7, 6.4, 2.2 Hz, 1H), 4.31 (d, $J$ = 2.8 Hz, 2H), 4.21 (s, 5H), 4.03 (m, $J$ = 12.5, 6.1, 3.1 Hz, 1H), 3.59 (dt, $J$ = 11.0, 1.9 Hz, 1H), 2.89 (dt, $J$ = 11.5, 2.0 Hz, 1H), 2.59 – 2.49 (m, 1H), 2.19 – 2.09 (m, 1H), 1.41 (d, $J$ = 6.3 Hz, 3H), 0.99 (d, $J$ = 6.3 Hz, 3H).

**$^{13}$C NMR (100 MHz, CDCl$_3$)** δ 169.6, 148.2, 139.2, 136.4, 136.3, 128.4, 127.7, 121.6, 121.2, 117.6, 111.9, 72.1, 71.2, 70.8, 67.1, 66.6, 63.7, 59.8, 58.4, 19.6, 19.0.

**HRMS (ESI)** m/z: [M]$^+$ Calcd for $C_{26}H_{27}FeN_3O_2$ 469.1453; found: 469.1448.

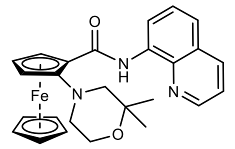


**1-(2,2-Dimethylmorpholino)-2-((8-quinolinylamino)carbonyl)ferrocene (4b)**

A purification by flash chromatography in petroleum ether : ethyl acetate= 4 : 1 to give **4b** as yellow foam (12.2 mg, 26%).

**$^1$H NMR (400 MHz, Chloroform-*d*)** δ 11.59 (s, 1H), 8.94 – 8.88 (m, 2H), 8.20 (dd, $J$ = 8.3, 1.7 Hz, 1H), 7.62 – 7.52 (m, 2H), 7.48 (dd, $J$ = 8.3, 4.2 Hz, 1H), 4.90 (dd, $J$ = 2.8, 1.6 Hz, 1H), 4.32 – 4.27 (m, 2H), 4.23 (s, 5H), 4.09 (m, $J$ = 11.1, 7.5, 3.0 Hz, 1H), 3.95 – 3.79 (m, 1H), 3.03 – 2.80 (m, 3H), 2.64 (d, $J$ = 11.2 Hz, 1H), 1.60 (s, 3H), 1.26 (s, 3H).

**$^{13}$C NMR (100 MHz, CDCl$_3$)** δ 169.6, 148.1, 139.3, 136.5, 135.6, 128.4, 127.6, 121.6, 121.5, 118.3, 112.6, 72.2, 71.5, 70.8, 67.3, 66.3, 63.9, 61.6, 59.3, 56.0, 26.2, 24.8.

**HRMS (ESI)** m/z: $[M]^+$ Calcd for $C_{26}H_{27}FeN_3O_2$ 469.1453; found: 469.1450.

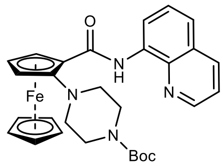


**1-(4-*tert*-Butoxycarbonyl piperazino)-2-((8-quinolinylamino)carbonyl)ferrocene (4c)**

A purification by flash chromatography in petroleum ether : ethyl acetate= 4 : 1 to give **4c** as yellow foam(24.3mg, 45%).

**$^{1}$H NMR (400 MHz, Chloroform-*d*)** δ 12.31 (s, 1H), 9.00 (dd, $J$ = 7.6, 1.4 Hz, 1H), 8.86 (dd, $J$ = 4.2, 1.6 Hz, 1H), 8.19 (dd, $J$ = 8.3, 1.7 Hz, 1H), 7.58 (t, $J$ = 7.9 Hz, 1H), 7.55 – 7.45 (m, 2H), 4.94 (dd, $J$ = 2.6, 1.6 Hz, 1H), 4.31 (d, $J$ = 2.4 Hz, 2H), 4.22 (s, 5H), 3.75 (s, 4H), 2.88 (dt, $J$ = 10.8, 5.0 Hz, 4H), 1.47 (s, 9H).

**$^{13}$C NMR (100 MHz, CDCl$_3$)** δ 169.6, 155.0, 148.1, 139.1, 136.5, 136.3, 128.5, 127.8, 121.7, 121.2, 117.5, 112.1, 80.0, 72.2, 70.9, 67.0, 66.6, 59.9, 54.8, 28.6.

**HRMS (ESI)** m/z: $[M + Na]^+$ Calcd for $C_{29}H_{32}FeN_4O_3Na$ 563.1716; found: 563.1719.

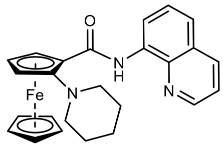


**1-Piperidino-2-((8-quinolinylamino)carbonyl)ferrocene (4d)**

A purification by flash chromatography in petroleum ether : tetrahydrofuran = 12 : 1 to give **4d** as yellow foam (37.3 mg, 85%).

**$^{1}$H NMR (400 MHz, Chloroform-*d*)** δ 12.44 (s, 1H), 9.00 (d, $J$ = 7.6 Hz, 1H), 8.91 (d, $J$ = 3.2 Hz, 1H), 8.18 (d, $J$ = 8.2 Hz, 1H), 7.58 (t, $J$ = 7.9 Hz, 1H), 7.54 – 7.45 (m, 2H), 4.89 (s, 1H), 4.33 – 4.25 (m, 2H), 4.20 (s, 5H), 3.06 (s, 2H), 2.85 (m, $J$ = 11.2, 5.4 Hz, 2H), 1.87 (t, $J$ = 5.9 Hz, 4H), 1.58 (s, 2H).

**$^{13}$C NMR (101 MHz, CDCl$_3$)** δ 170.0, 147.9, 139.4, 136.8, 136.2, 128.4, 127.7, 121.6, 121.0, 117.5, 113.9, 72.3, 70.8, 66.6, 66.2, 59.3, 56.6, 25.8, 24.6.

**HRMS (ESI)** m/z: [M + H]$^+$ Calcd for $C_{25}H_{26}FeN_3O$ 440.1347; found: 440.1422.

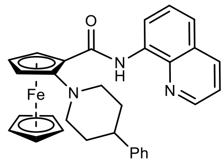


**1-(4-Phenylpiperidino)-2-((8-quinolinylamino)carbonyl)ferrocene (4e)**

A purification by flash chromatography in petroleum ether : ethyl acetate= 4 : 1 to give **4e** as yellow foam(30.9mg, 60%).

**$^1$H NMR (400 MHz, Chloroform-*d*)** δ 12.43 (s, 1H), 8.99 (dd, *J* = 7.6, 1.4 Hz, 1H), 8.90 (dd, *J* = 4.2, 1.7 Hz, 1H), 8.21 (dd, *J* = 8.3, 1.7 Hz, 1H), 7.60 (t, *J* = 7.9 Hz, 1H), 7.53 (dd, *J* = 8.2, 1.4 Hz, 1H), 7.47 (dd, *J* = 8.2, 4.2 Hz, 1H), 7.28 (s, 2H), 7.24 (s, 1H), 7.20 (d, *J* = 7.2 Hz, 1H), 7.12 (d, *J* = 6.9 Hz, 2H), 4.91 (dd, *J* = 2.8, 1.6 Hz, 1H), 4.35 (dd, *J* = 2.7, 1.6 Hz, 1H), 4.30 (t, *J* = 2.7 Hz, 1H), 4.23 (s, 5H), 3.94 (d, *J* = 11.4 Hz, 1H), 3.20 (d, *J* = 11.8 Hz, 1H), 2.88 (t, *J* = 11.4 Hz, 1H), 2.70 – 2.59 (m, 1H), 2.52 (m, *J* = 11.8, 7.6, 3.1 Hz, 2H), 2.14 (d, *J* = 12.3 Hz, 1H), 2.07 – 1.94 (m, 1H), 1.65 (d, *J* = 12.7 Hz, 1H).

**$^{13}$C NMR (100 MHz, $CDCl_3$)** δ 169.9, 148.4, 146.3, 139.4, 136.4, 128.5, 128.5, 127.7, 127.0, 126.3, 121.4, 121.1, 117.6, 113.3, 72.4, 70.9, 66.6, 66.2, 59.2, 59.1, 53.3, 42.6, 33.6, 32.7.

**HRMS (ESI)** m/z: [M]$^+$ Calcd for $C_{31}H_{29}FeN_3O$ 515.1660; found: 515.1656.

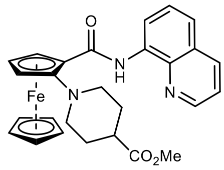


**1-(4-Methoxycarbonylpiperidino)-2-((8-quinolinylamino)carbonyl)ferrocene (4f)**

A purification by flash chromatography in petroleum ether : ethyl acetate= 4 : 1 to give **4f** as yellow foam (28.3 mg, 57%).

**$^1$H NMR (400 MHz, Chloroform-*d*)** δ 12.32 (s, 1H), 9.03 (dd, *J* = 7.7, 1.5 Hz, 1H), 9.00 (dd, *J* = 4.3, 1.7 Hz, 1H), 8.17 (dd, *J* = 8.2, 1.8 Hz, 1H), 7.57 (t, *J* = 7.9 Hz, 1H), 7.53 – 7.47 (m, 2H), 4.92 (dd, *J* = 2.6, 1.7 Hz, 1H), 4.31 – 4.27 (m, 2H), 4.20 (s, 5H), 3.76 (d, *J* = 11.3 Hz, 1H), 3.69 (s, 3H), 3.11 (dt, *J* = 11.3, 3.7 Hz, 1H), 2.74 (td, *J* = 11.2, 2.6 Hz, 1H), 2.57 – 2.39 (m, 3H), 2.18 (m, *J* = 15.2, 7.3, 4.0 Hz, 2H), 1.81 (d, *J* = 12.9 Hz, 1H).

**$^{13}$C NMR (100 MHz, CDCl$_3$)** δ 175.8, 169.8, 148.5, 139.2, 136.5, 136.2, 128.4, 127.6, 121.6, 121.1, 117.4, 112.8, 72.2, 70.9, 66.9, 66.4, 59.5, 57.4, 52.7, 51.8, 41.3, 28.4, 28.0.

**HRMS (ESI)** m/z: [M]$^+$ Calcd for $C_{27}H_{27}FeN_3O_3$ 497.1402; found: 497.1398.

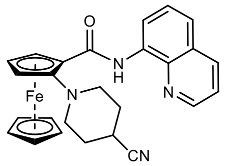


**1-(4-Cyanopiperidin-1-yl)-2-((8-quinolinylamino)carbonyl)ferrocene (4g)**

A purification by flash chromatography in petroleum ether : ethyl acetate= 4 : 1 to give **4g** as yellow foam (28.3mg, 61%).

**$^1$H NMR (400 MHz, Chloroform-*d*)** δ 12.18 (s, 1H), 9.04 (dd, *J* = 7.6, 1.5 Hz, 1H), 8.94 (dd, *J* = 4.3, 1.7 Hz, 1H), 8.21 (dd, *J* = 8.2, 1.7 Hz, 1H), 7.59 (t, *J* = 7.9 Hz, 1H), 7.57 – 7.48 (m, 2H), 4.94 (dd, *J* = 2.7, 1.6 Hz, 1H), 4.31 (p, *J* = 2.7 Hz, 2H), 4.21 (s, 5H), 3.49 (s, 1H), 3.16 – 3.06 (m, 1H), 3.00 – 2.67 (m, 3H), 2.34 (qt, *J* = 8.7, 3.6 Hz, 3H), 2.16 (s, 1H).

**$^{13}$C NMR (100 MHz, CDCl$_3$)** δ 169.6, 148.0, 139.0, 136.6, 136.3, 128.5, 127.8, 122.0, 121.8, 121.3, 117.6, 112.1, 72.2, 70.9, 67.1, 66.6, 59.8, 28.8, 28.6, 26.4.

**HRMS (ESI)** m/z: [M]$^+$ Calcd for $C_{26}H_{24}FeN_4O$ 464.1300; found: 464.1292.

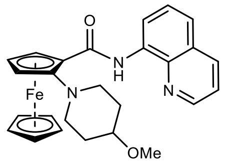


**1-(4-Methoxypiperidino)-2-((8-quinolinylamino)carbonyl)ferrocene (4h)**

A purification by flash chromatography in petroleum ether : ethyl acetate= 4 : 1 to give **4h** as yellow foam (26.3 mg, 56%).

**$^1$H NMR (400 MHz, Chloroform-*d*)** δ 12.42 (s, 1H), 9.03 (dd, *J* = 7.6, 1.4 Hz, 1H), 8.94 (dd, *J* = 4.2, 1.7 Hz, 1H), 8.17 (dd, *J* = 8.3, 1.7 Hz, 1H), 7.57 (t, *J* = 7.9 Hz, 1H), 7.54 – 7.44 (m, 2H), 4.92 (dd, *J* = 2.8, 1.6 Hz, 1H), 4.31 (dd, *J* = 2.7, 1.5 Hz, 1H), 4.28 (t, *J* = 2.7 Hz, 1H), 4.20 (s, 5H), 3.37 (s, 5H), 3.09 (dt, *J* = 10.1, 4.3 Hz, 1H), 2.76 (m, *J* = 11.7, 8.8, 3.4 Hz, 2H), 2.21 – 2.00 (m, 4H).

**$^{13}$C NMR (100 MHz, CDCl$_3$)** δ 169.9, 148.1, 139.2, 136.5, 136.2, 128.4, 127.6, 121.7, 121.1, 117.5, 112.8, 72.2, 70.8, 66.7, 66.4, 59.6, 55.8, 52.3, 30.7, 30.6.

**HRMS (ESI)** m/z: $[M]^+$ Calcd for $C_{26}H_{27}FeN_3O_2$ 469.1453; found: 469.1449.

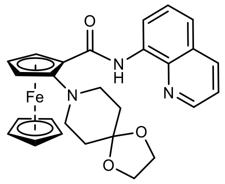

**1-(1,4-Dioxa-8-azaspiro[4.5]decane)-2-((8-quinolinylamino)carbonyl)ferrocene (4i)**

A purification by flash chromatography in petroleum ether : ethyl acetate= 4 : 1 to give **4i** as yellow foam (31.3 mg, 63%).

**$^1$H NMR (400 MHz, Chloroform-*d*)** δ 12.45 (s, 1H), 9.06 (dd, *J* = 7.7, 1.5 Hz, 1H), 8.98 (dd, *J* = 4.2, 1.7 Hz, 1H), 8.17 (dd, *J* = 8.3, 1.7 Hz, 1H), 7.57 (t, *J* = 7.9 Hz, 1H), 7.53 – 7.47 (m, 2H), 4.93 (dd, *J* = 2.9, 1.5 Hz, 1H), 4.34 (t, *J* = 2.1 Hz, 1H), 4.29 (t, *J* = 2.7 Hz, 1H), 4.20 (s, 5H), 4.00 (s, 4H), 3.21 (s, 2H), 3.02 (dt, *J* = 11.3, 5.5 Hz, 2H), 2.23 – 1.98 (m, 4H).

**$^{13}$C NMR (100 MHz, $CDCl_3$)** δ 169.9, 148.1, 139.2, 136.6, 136.3, 128.4, 127.7, 121.8, 121.1, 117.4, 112.4, 107.5, 72.1, 70.9, 66.8, 66.5, 64.5, 60.0, 53.7, 34.9.

**HRMS (ESI)** m/z: $[M]^+$ Calcd for $C_{27}H_{27}FeN_3O_3$ 497.1402; found: 497.1394.

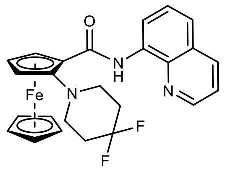

**1-(4,4-Difluoropiperidino)-2-((8-quinolinylamino)carbonyl)ferrocene (4j)**

A purification by flash chromatography in petroleum ether : ethyl acetate= 4 : 1 to give **4j** as yellow foam (10.9 mg, 23%).

**$^1$H NMR (400 MHz, Chloroform-*d*)** δ 12.27 (s, 1H), 9.08 (dd, *J* = 7.7, 1.5 Hz, 1H), 8.87 (dd, *J* = 4.3, 1.7 Hz, 1H), 8.20 (dd, *J* = 8.2, 1.7 Hz, 1H), 7.59 (t, *J* = 7.9 Hz, 1H), 7.56 – 7.47 (m, 2H), 4.96 (t, *J* = 2.2 Hz, 1H), 4.33 (d, *J* = 2.2 Hz, 2H), 4.21 (s, 5H), 3.26 (s, 2H), 3.04 (m, *J* = 11.7, 7.0, 4.4 Hz, 2H), 2.58 – 2.24 (m, 4H).

**$^{13}$C NMR (100 MHz, $CDCl_3$)** δ 169.6, 147.8, 139.0, 136.6, 136.3, 128.5, 127.8, 121.8, 121.3, 117.6, 111.4, 72.2, 70.9, 67.1, 66.7, 60.1, 34.4, 34.2, 33.9.

**HRMS (ESI)** m/z: $[M + Na]^+$ Calcd for $C_{25}H_{23}F_2FeN_3ONa$ 498.1051; found: 498.1054.

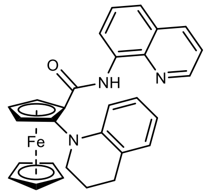


**1-(1,2,3,4-Tetrahydroquinolino)-2-((8-quinolinylamino)carbonyl)ferrocene (4k)**

A purification by flash chromatography in petroleum ether : tetrahydrofuran = 12 : 1 to give **4k** as yellow foam (2.4 mg, trace).

**$^1$H NMR (400 MHz, Chloroform-*d*)** δ 11.35 (s, 1H), 8.84 (dd, *J* = 7.7, 1.4 Hz, 1H), 8.68 (dd, *J* = 4.2, 1.7 Hz, 1H), 8.09 (dd, *J* = 8.3, 1.7 Hz, 1H), 7.50 (t, *J* = 7.9 Hz, 1H), 7.43 (dd, *J* = 8.3, 1.4 Hz, 1H), 7.39 (dd, *J* = 8.3, 4.2 Hz, 1H), 6.98 – 6.91 (m, 1H), 6.79 (t, *J* = 7.8 Hz, 1H), 6.58 (t, *J* = 7.3 Hz, 1H), 6.36 (d, *J* = 8.3 Hz, 1H), 5.11 (dd, *J* = 2.9, 1.6 Hz, 1H), 4.53 – 4.44 (m, 2H), 4.34 (s, 5H), 4.31 (dd, *J* = 2.7, 1.6 Hz, 1H), 3.76 (td, *J* = 10.6, 3.1 Hz, 1H), 2.98 (m, *J* = 11.4, 5.6 Hz, 2H), 2.85 (m, *J* = 11.0, 10.0, 5.3 Hz, 1H), 2.21 (d, *J* = 5.4 Hz, 1H).

**$^{13}$C NMR (101 MHz, CDCl$_3$)** δ 169.1, 147.7, 139.3, 138.5, 136.2, 135.0, 131.9, 128.6, 128.2, 127.6, 126.7, 124.6, 121.5, 121.4, 121.2, 119.1, 117.3, 115.0, 74.8, 71.2, 67.6, 67.0, 65.2, 53.6, 27.8, 22.8.

**HRMS (ESI)** m/z: [M + H]$^+$ Calcd for $C_{29}H_{26}FeN_3O$ 488.1347; found: 488.1415.

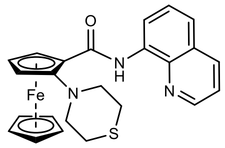


**1-Thiomorpholino-2-((8-quinolinylamino)carbonyl)ferrocene (4l)**

A purification by flash chromatography in petroleum ether : ethyl acetate= 4 : 1 to give **4l** as yellow foam (13.3mg, 29%).

**$^1$H NMR (400 MHz, Chloroform-*d*)** δ 12.29 (s, 1H), 9.10 – 8.99 (m, 2H), 8.21 (dd, *J* = 8.2, 1.7 Hz, 1H), 7.59 (t, *J* = 7.9 Hz, 1H), 7.55 – 7.49 (m, 2H), 4.94 (dd, *J* = 2.8, 1.6 Hz, 1H), 4.32 (t, *J* = 2.8 Hz, 1H), 4.29 (dd, *J* = 2.8, 1.6 Hz, 1H), 4.20 (s, 5H), 3.46 (s, 2H), 3.12 (m, *J* = 21.0, 10.0 Hz, 6H).

**$^{13}$C NMR (100 MHz, CDCl$_3$)** δ 169.6, 148.1, 139.0, 136.5, 136.4, 128.5, 127.8, 121.8, 121.2, 117.5, 113.6, 72.1, 70.9, 66.9, 66.7, 60.3, 57.5, 27.6.

**HRMS (ESI)** m/z: [M + Na]$^+$ Calcd for $C_{24}H_{23}FeN_3OSNa$ 480.0803; found: 480.0804.

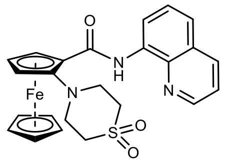

**1-(4,4-Dioxidothiomorpholino )-2-((8-quinolinylamino)carbonyl)ferrocene (4m)**

A purification by flash chromatography in petroleum ether : ethyl acetate= 4 : 1 to give **4m** as yellow foam (32.8 mg, 67%).

**$^{1}$H NMR (400 MHz, Chloroform-*d*)** δ 12.02 (s, 1H), 9.12 (dd, *J* = 7.5, 1.6 Hz, 1H), 9.06 (dd, *J* = 4.3, 1.7 Hz, 1H), 8.24 (dd, *J* = 8.3, 1.7 Hz, 1H), 7.61 (t, *J* = 7.8 Hz, 2H), 7.59 – 7.51 (m, 2H), 5.02 (dd, *J* = 2.9, 1.5 Hz, 1H), 4.39 (t, *J* = 2.8 Hz, 1H), 4.35 (dd, *J* = 2.7, 1.5 Hz, 1H), 4.23 (s, 5H), 3.73 (s, 4H), 3.46 (d, *J* = 9.4 Hz, 4H).

**$^{13}$C NMR (100 MHz, CDCl$_3$)** δ 169.0, 148.4, 138.8, 137.1, 136.0, 128.6, 127.9, 122.1, 121.7, 117.8, 110.8, 72.0, 71.1, 67.6, 67.2, 61.0, 53.8, 51.9.

**HRMS (ESI)** m/z: [M + Na]$^{+}$ Calcd for $C_{24}H_{23}FeN_3O_3SNa$ 512.0702; found: 512.0700.

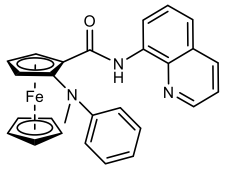

**1-（*N*-Methylphenyl)-2-((8-quinolinylamino)carbonyl)ferrocene (4n)**

A purification by flash chromatography in petroleum ether : tetrahydrofuran= 12 : 1 to give **4n** as yellow foam (8.3 mg, 18%).

**$^{1}$H NMR (400 MHz, Chloroform-*d*)** δ 11.69 (s, 1H), 8.82 – 8.76 (m, 2H), 8.10 (dd, *J* = 8.2, 1.7 Hz, 1H), 7.49 (t, *J* = 7.9 Hz, 1H), 7.45 – 7.39 (m, 2H), 7.06 (dd, *J* = 8.8, 7.2 Hz, 2H), 6.78 (d, *J* = 7.8 Hz, 2H), 6.69 (t, *J* = 7.3 Hz, 1H), 5.09 (dd, *J* = 2.9, 1.5 Hz, 1H), 4.51 (t, *J* = 2.8 Hz, 1H), 4.35 (s, 6H), 3.88 (s, 3H).

**$^{13}$C NMR (100 MHz, CDCl$_3$)** δ 168.6, 150.9, 148.1, 139.1, 136.1, 135.7, 128.6, 128.1, 127.6, 121.5, 120.9, 119.5, 116.6, 116.3, 107.3, 74.1, 71.2, 67.3, 67.1, 65.4, 44.1.

**HRMS (ESI)** m/z: [M + Na]$^{+}$ Calcd for $C_{27}H_{23}FeN_3ONa$ 484.1083; found: 484.1086.

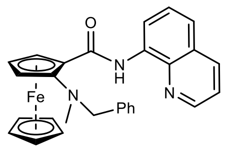

**1-(*N*-Benzylmethyl)-2-((8-quinolinylamino)carbonyl)ferrocene (4o)**

A purification by flash chromatography in petroleum ether : tetrahydrofuran = 12 : 1 to give **4o** as yellow foam (7.1 mg, 15%).

**$^{1}$H NMR (400 MHz, Chloroform-*d*)** δ 12.27 (s, 1H), 8.93 (d, *J* = 7.6 Hz, 1H), 8.59 (dd, *J* = 4.2, 1.7 Hz, 1H), 8.16 (dd, *J* = 8.2, 1.7 Hz, 1H), 7.58 (t, *J* = 7.9 Hz, 1H), 7.52 (d, *J* = 8.2 Hz, 1H), 7.45 – 7.38 (m, 3H), 7.26 – 7.19 (m, 3H), 4.95 (s, 1H), 4.49 (d, *J* = 13.1 Hz, 1H), 4.32 (s, 1H), 4.26 (s, 6H), 3.85 (d, *J* = 13.1 Hz, 1H), 2.71 (s, 3H).

**$^{13}$C NMR (100 MHz, $CDCl_3$)** δ 169.7, 148.9, 148.3, 139.5, 137.2, 136.1, 129.9, 128.3, 128.2, 127.6, 127.4, 121.4, 121.2, 117.7, 113.3, 72.5, 70.9, 66.9, 66.3, 62.9, 60.5, 43.9.

**HRMS (ESI)** m/z: $[M]^+$ Calcd for $C_{28}H_{25}FeN_3O$ 475.1347; found: 475.1340.

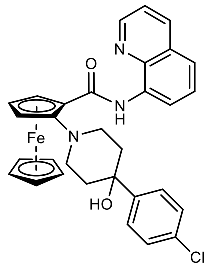

**1-(4-(4-Chlorophenyl)-4-hydroxypiperidin-1-yl)-2-((8-quinolinylamino)carbonyl)ferrocene (4p)**

A purification by flash chromatography in petroleum ether : ethyl acetate= 4 : 1 to give **4p** as yellow foam (35.6 mg, 63%).

**$^{1}$H NMR (400 MHz, Chloroform-*d*)** δ 12.41 (s, 1H), 8.96 (d, *J* = 7.5 Hz, 1H), 8.71 (d, *J* = 2.6 Hz, 1H), 8.21 (d, *J* = 8.3 Hz, 1H), 7.59 (t, *J* = 7.9 Hz, 1H), 7.53 (d, *J* = 8.2 Hz, 1H), 7.43 (dd, *J* = 8.2, 4.2 Hz, 1H), 7.28 (d, *J* = 8.7 Hz, 3H), 7.23 (d, *J* = 8.6 Hz, 3H), 4.89 (s, 1H), 4.38 (s, 1H), 4.30 (t, *J* = 2.7 Hz, 1H), 4.21 (s, 5H), 3.68 (d, *J* = 10.7 Hz, 1H), 3.31 – 3.20 (t, 1H), 2.99 – 2.86 (m, 2H), 2.71 (m, *J* = 12.7, 4.4 Hz, 1H), 2.41 – 2.29 (m, 1H), 2.13 – 2.02 (m, 1H), 1.88 (s, 1H), 1.60 (d, *J* = 13.5 Hz, 1H).

**$^{13}$C NMR (100 MHz, $CDCl_3$)** δ 170.0, 148.2, 147.0, 139.4, 136.6, 136.3, 133.0, 128.6, 128.4, 127.8,

126.4, 121.3, 121.2, 117.8, 112.9, 72.2, 71.0, 70.9, 66.7, 66.4, 59.6, 53.9, 48.6, 38.7, 37.8.

**HRMS (ESI)** m/z: $[M]^+$ Calcd for $C_{31}H_{28}ClFeN_3O_2$ 565.1219; found: 565.1215.

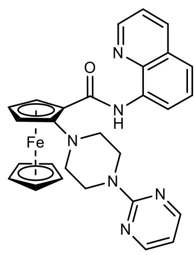

**1-(4-(Pyrimidin-2-yl)piperazin-1-yl)-2-((8-quinolinylamino)carbonyl)ferrocene (4q)**

A purification by flash chromatography in petroleum ether : ethyl acetate= 4 : 1 to give **4q** as yellow foam (31.1 mg, 60%).

**$^1$H NMR (400 MHz, Chloroform-*d*)** δ 12.43 (s, 1H), 9.02 (d, *J* = 7.7 Hz, 1H), 8.82 (d, *J* = 2.5 Hz, 1H), 8.32 (d, *J* = 4.7 Hz, 2H), 8.16 (d, *J* = 6.6 Hz, 1H), 7.59 (t, *J* = 7.9 Hz, 1H), 7.51 (d, *J* = 6.8 Hz, 1H), 7.42 (dd, *J* = 8.3, 4.2 Hz, 1H), 6.50 (t, *J* = 4.8 Hz, 1H), 4.95 (s, 1H), 4.31 (s, 2H), 4.24 (s, 5H), 4.19 m, *J* = 6.3, 3.3 Hz, 4H), 3.17 (m, *J* = 10.7, 5.0 Hz, 2H), 2.99 (m, *J* = 10.8, 4.9 Hz, 2H).

**$^{13}$C NMR (100 MHz, $CDCl_3$)** δ 169.7, 161.8, 157.9, 148.1, 139.2, 136.4, 128.5, 127.8, 121.7, 121.2, 117.6, 112.2, 110.0, 100.9, 72.3, 70.9, 67.1, 66.6, 59.9, 55.0, 43.6.

**HRMS (ESI)** m/z: $[M + Na]^+$ Calcd for $C_{28}H_{26}FeN_6ONa$ 541.1410; found: 541.1412.

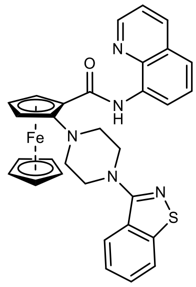

**1-(4-(Benzo[*d*]isothiazol-3-yl)piperazin-1-yl)-2-((8-quinolinylamino)carbonyl)ferrocene (4r)**

A purification by flash chromatography in petroleum ether : ethyl acetate= 4 : 1 to give **4r** as yellow foam (40.1 mg,70%,).

**$^1$H NMR (400 MHz, Chloroform-*d*)** δ 12.40 (s, 1H), 9.02 (d, *J* = 6.3 Hz, 1H), 8.96 (d, *J* = 2.6 Hz, 1H), 8.15 (d, *J* = 8.2 Hz, 1H), 7.92 (d, *J* = 8.2 Hz, 1H), 7.83 (d, *J* = 8.1 Hz, 1H), 7.57 (t, *J* = 7.9 Hz,

1H), 7.54 – 7.41 (m, 3H), 7.35 (t, $J$ = 7.5 Hz, 1H), 4.98 (s, 1H), 4.41 (s, 1H), 4.35 (t, $J$ = 2.8 Hz, 1H), 4.25 (s, 5H), 3.93 (t, $J$ = 5.0 Hz, 4H), 3.41 – 3.32 (m, 2H), 3.24 – 3.14 (m, 2H).

**$^{13}$C NMR (100 MHz, $CDCl_3$)** δ 169.7, 164.2, 152.9, 148.6, 139.1, 136.4, 136.3, 127.7, 127.7, 124.1, 124.0, 121.8, 121.2, 120.7, 117.5, 112.1, 72.3, 70.9, 67.1, 66.6, 60.0, 55.0, 49.9.

**HRMS (ESI)** m/z: $[M + Na]^+$ Calcd for $C_{31}H_{27}FeN_5OSNa$ 596.1178; found: 596.1181.

# 6. Gram-scale synthesis

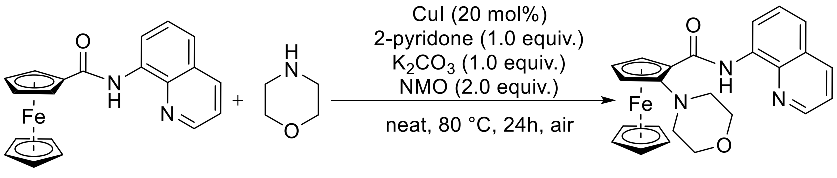


**1a** (6.0 mmol, 2.65 g) **2a** 30.0 mmol **3a** (1.32 g, 50%)

**Procedure for 6 mmol scale reaction of 1a:**

To a 50 mL Schlenk tube was added **1a** (6 mmol), **2a** (30.0 mmol), $K_2CO_3$ (840.0 mg, 1.0 equiv), CuI (228 mg, 20 mol %), NMO (1.4 g, 2.0 equiv) and 2-pyridone (570 mg, 1.0 equiv). The reaction mixture was stirred at 80 °C (aluminum heat transfer block) for 24 h. After cooling to room temperature, the mixture was diluted with DCM, The crude mixture was purified by flash column chromatography on silica gel (hexanes/ethyl acetate = 10:1 to 4:1) affording the desired product **3a** as yellow solid, 1.32 g, 50% yield.

## 7. Removal of directing group.

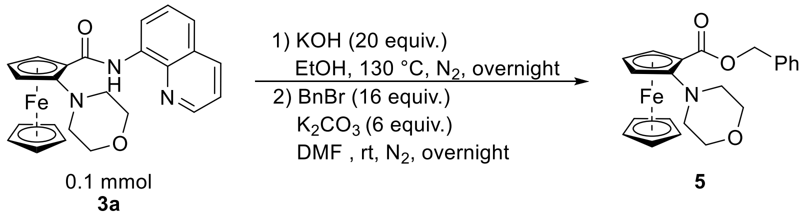


The product **3a** (44.1 mg, 0.1 mmol, 1.0 equiv.) was dissolved in MeOH (1 mL) and KOH (1.12g, 20 mmol, 20.0 equiv.) was added. The reaction mixture was refluxed with stirring for 12 h (The reaction was detected by TLC). The reaction mixture was cooled to room temperature and extracted with ethyl acetate (30 mL) and water (2 × 30 mL). The combined aqueous phase was acidified with 2 M HCl to pH = 2 and extracted with ether (3 × 30 mL). The combined ether phase was washed with brine (45 mL) and dried with $Na_2SO_4$ and was concentrated under reduced pressure to give the crude product that used directly in the next step.

$K_2CO_3$(0.6 mmol), DMF (2.0 ml) and (bromomethyl)benzene (1.6 mmol) were charged in reaction vessel equipped with magnetic stirring bar under nitrogen atmosphere. The mixture was stirred at rt for 12 h. Ethyl acetate (10 mL) and 10% aqueous $Na_2S_2O_3$ (20 mL) were added to the mixture and the organic phase extracted by three potions of EtOAc. Combined organic layer was dried over $MgSO_4$ and the solvent evaporated in vacuo. Further purification was carried out by silica gel column chromatography using EtOAc/hexane to afford the desired **5** (30.4 mg, 75% yield)

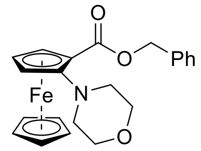

H

**Benzyl 2-morpholinoferrocenezoate (5).**

**$^1$H NMR (400 MHz, Chloroform-*d*)** δ 7.45 (d, $J$ = 6.7 Hz, 2H), 7.40 (t, $J$ = 7.2 Hz, 2H), 7.34 (t, $J$ = 7.1 Hz, 1H), 5.32 (d, $J$ = 12.4 Hz, 1H), 5.20 (d, $J$ = 12.4 Hz, 1H), 4.73 (dd, $J$ = 2.8, 1.6 Hz, 1H), 4.25 (dd, $J$ = 2.8, 1.7 Hz, 1H), 4.18 (s, 6H), 3.79 (t, $J$ = 4.7 Hz, 4H), 3.08 (m, $J$ = 10.1, 4.9 Hz, 2H), 2.91 – 2.81 (m, 2H).

**$^{13}$C NMR (100 MHz, $CDCl_3$)** δ 171.4, 136.7, 128.7, 128.3, 128.3, 114.5, 69.7, 68.9, 67.1, 65.9, 65.8, 62.2, 61.7, 53.7.

**HRMS (ESI)** m/z: $[M + H]^+$ Calcd for $C_{22}H_{24}FeNO_3$ 406.1107; found: 406.1098.

# 8. Mechanistic Experiments.

## 8.1 Synthesis of deuterated substrate.

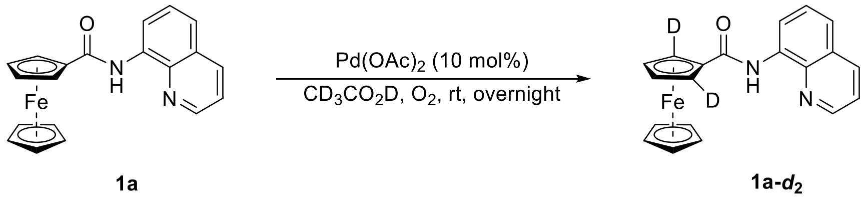


**1a** (1.0 mmol), $CD_3CO_2D$ (10 ml) and $Pd(OAc)_2$ (10 mol %) were charged in reaction vessel equipped with magnetic stirring bar under $O_2$ atmosphere. The mixture was at rt. for 12 h. Ethyl acetate (10 mL) and 10% aqueous $NaHCO_3$ (10 mL) were added to the mixture and the organic phase extracted by three potions of EtOAc. Combined organic layer was dried over magnesium sulfate and the solvent evaporated in vacuo. Further purification was carried out by silica gel column chromatography using EtOAc/hexane to afford the desired **2a** (27.3 mg, 81% yield).

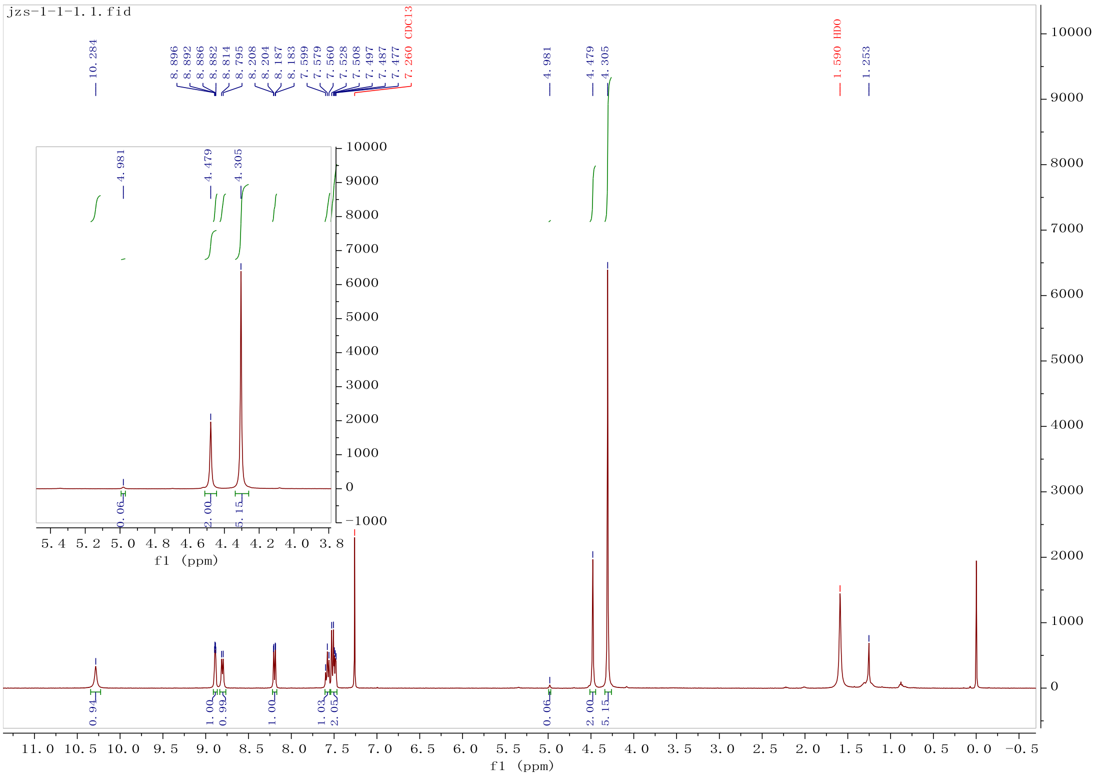

## 8.2 H/D exchange experiment

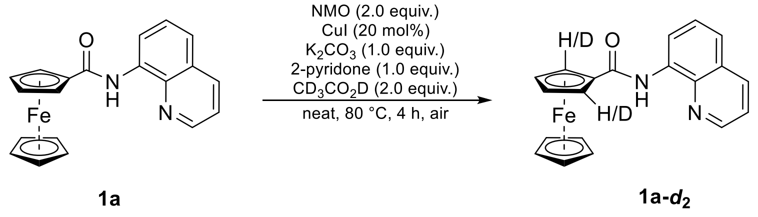


To a 10 mL Schlenk tube was added **1a** (0.1 mmol), $CD_3CO_2D$ (0.2 mmol), $K_2CO_3$ (14.0 mg, 1.0 equiv), CuI (3.8 mg, 20 mol %), NMO (23.4 mg, 2.0 equiv) and 2-pyridone (9.5 mg, 1.0 equiv), stirred at 80 °C (aluminum heat transfer block) for 4 h. After cooling to room temperature, the mixture was diluted with DCM, the resulting residue was purified by preparative TLC using Hexane/EtOAc as the eluent to afford the desired product. No H/D exchange was observed at the *ortho*-position of **1a.**

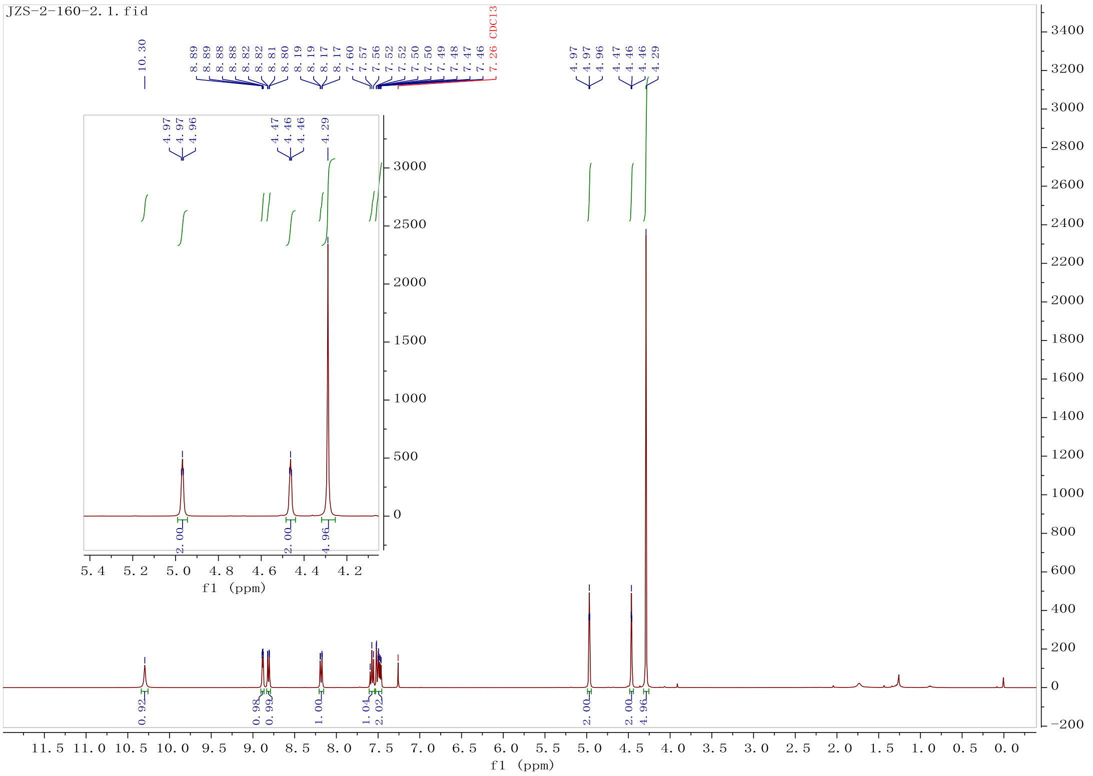

## 8.3 KIE experiment

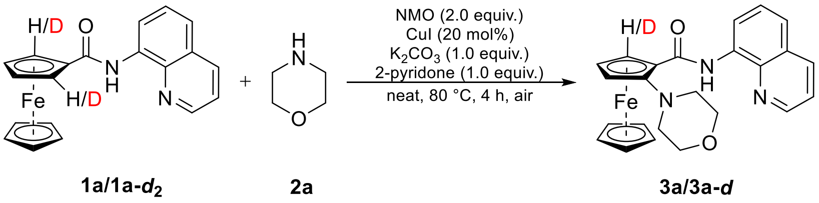


To a 50 mL Schlenk tube was added **1a** (0.1 mmol), or **1a-*d*₂** (0.1 mmol), **2a** (0.5 mmol), $K_2CO_3$ (14.0 mg, 1.0 equiv), CuI (3.8 mg, 20 mol %), NMO (23.4 mg, 2.0 equiv) and 2-pyridone (9.5 mg, 1.0 equiv), the tube was sealed up a cap and evacuated then refilled with air and kept stirring at 80 °C (aluminum heat transfer block). Then immediately quenched with DCM. The corresponding yield of each product was determined by $^1$H NMR. A whole set of experiments was performed two times and their average values were used for the KIE calculation. KIE = $k_H/k_D$ = 2.4.

| **Time (min)** | **1** | **5** | **10** | **15** | **25** | **35** |
|---|---|---|---|---|---|---|
| **3a** | 0 | 6 | 13 | 25 | 40 | --- |
| **3a-*d*** | 0 | 2 | --- | 7 | 15 | 25 |

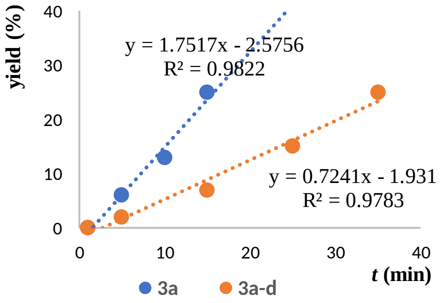

## 9. References.


1) Sattar, M.; Praveen, C.; Prasad, D.; Verma, A.; Kumar, S.; Kumar, S. *Adv. Synth. Catal*. **2016**, *358*, 240.

2) Ivan, S.; Jakub, V.; Dusan, B.; Andrej, K. *Tetrahedron*. **2015** *71* 8876-8884.

3) Daniela, H.; Harald, H.; and Peter, G. *J. Med. Chem*. **2009**, *52*, 6860–6870.

4) Zubeda, B.; Bhavania, D.; Sridharb, B.; Basireddy, V.; Subba, R. *Synthesis*. **2018**, *50*, 4089–4096.

5) Siu, Juno C.; Parry, Joseph B.; and Lin, S. *J. Am. Chem. Soc*. **2019**, *141*, 2825−2831.

# 10. NMR Spectra.

## 2b-1H NMR

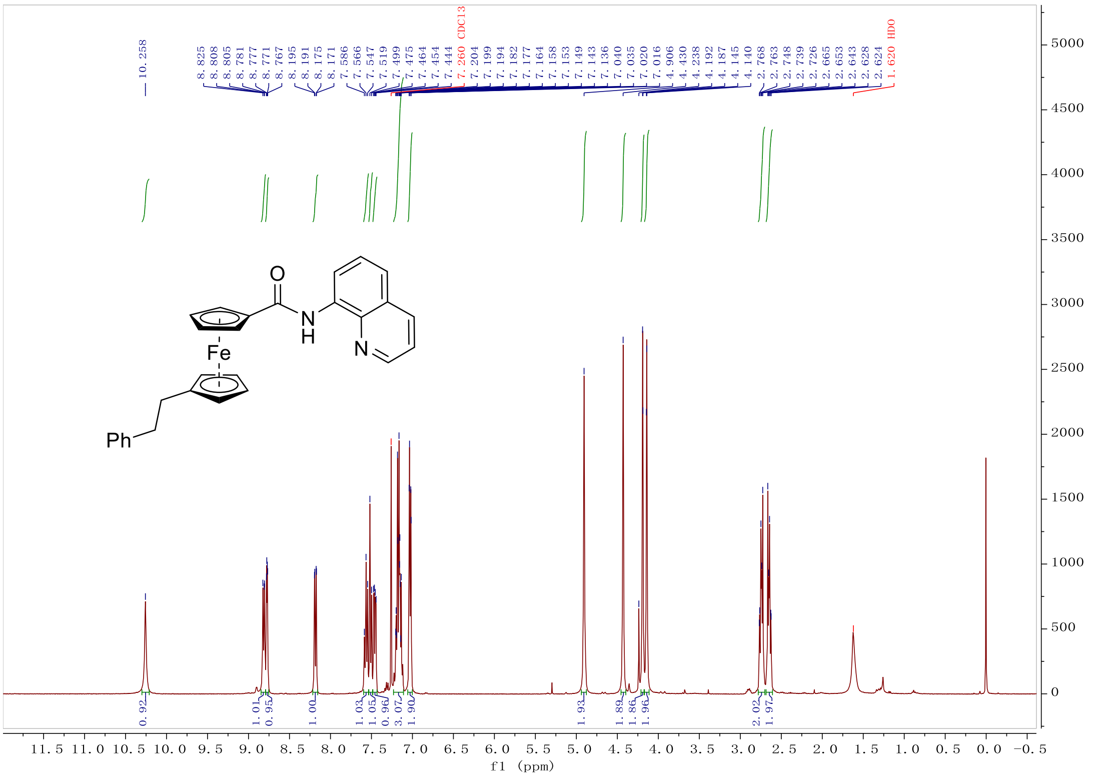

## 2b-13C NMR

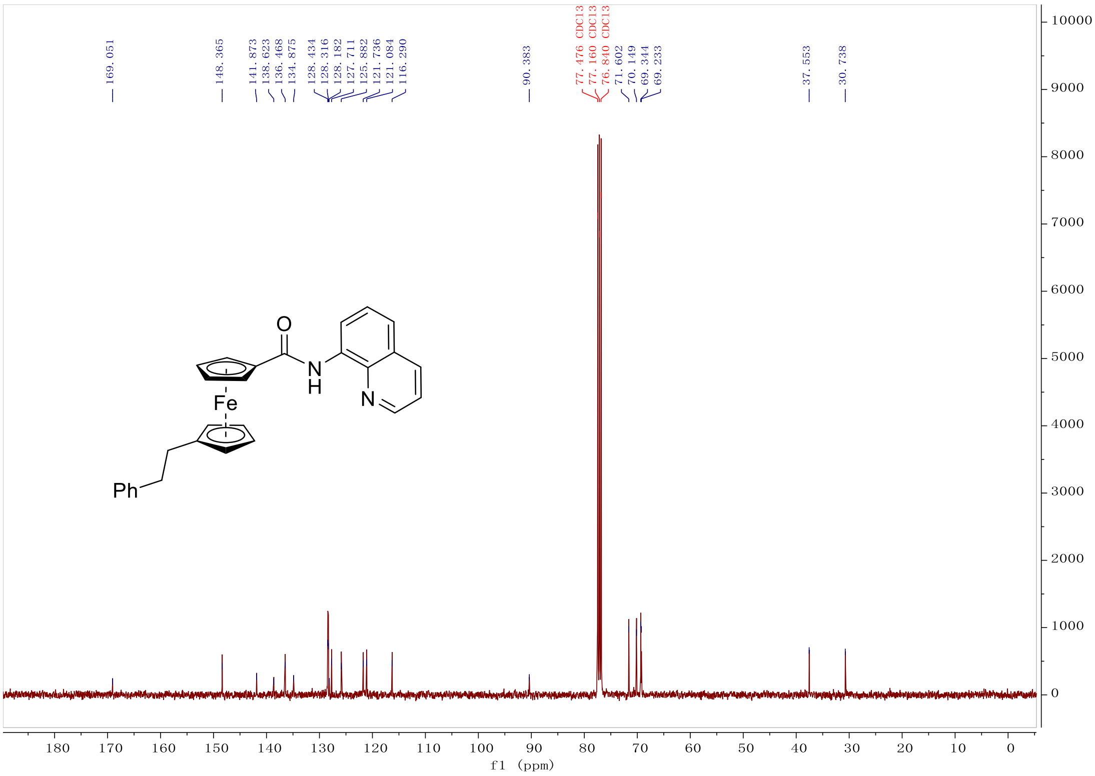

## 2c-$^{1}$H NMR

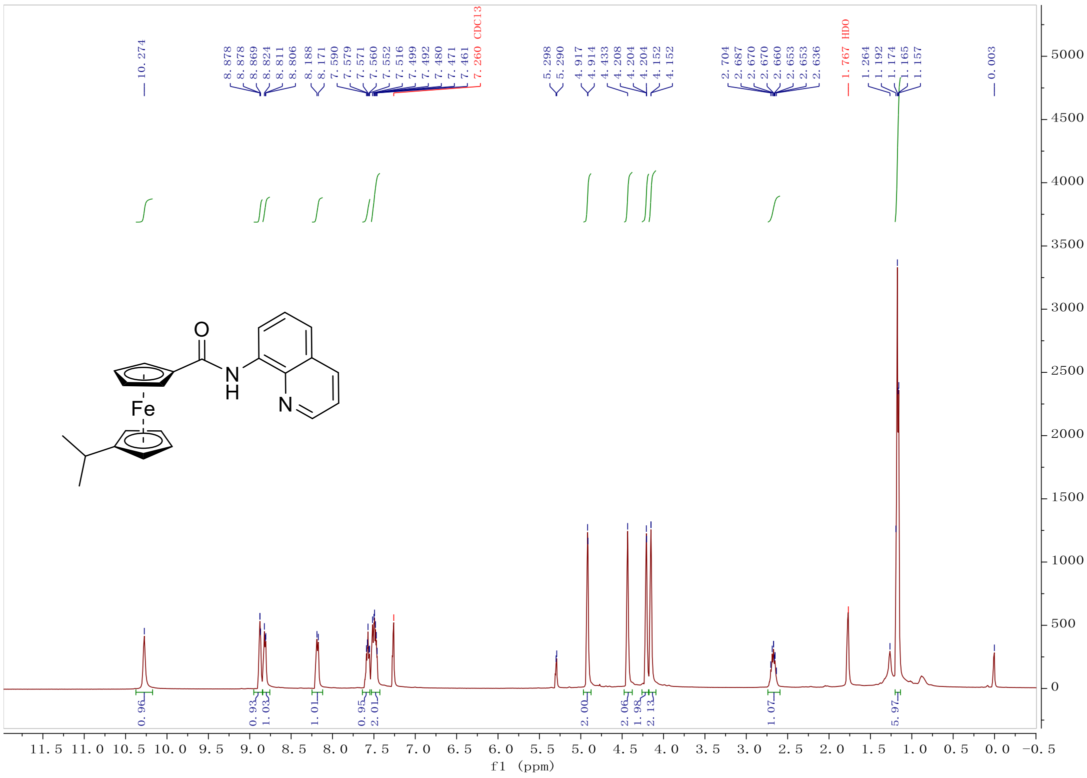

## 2c-$^{13}$C NMR

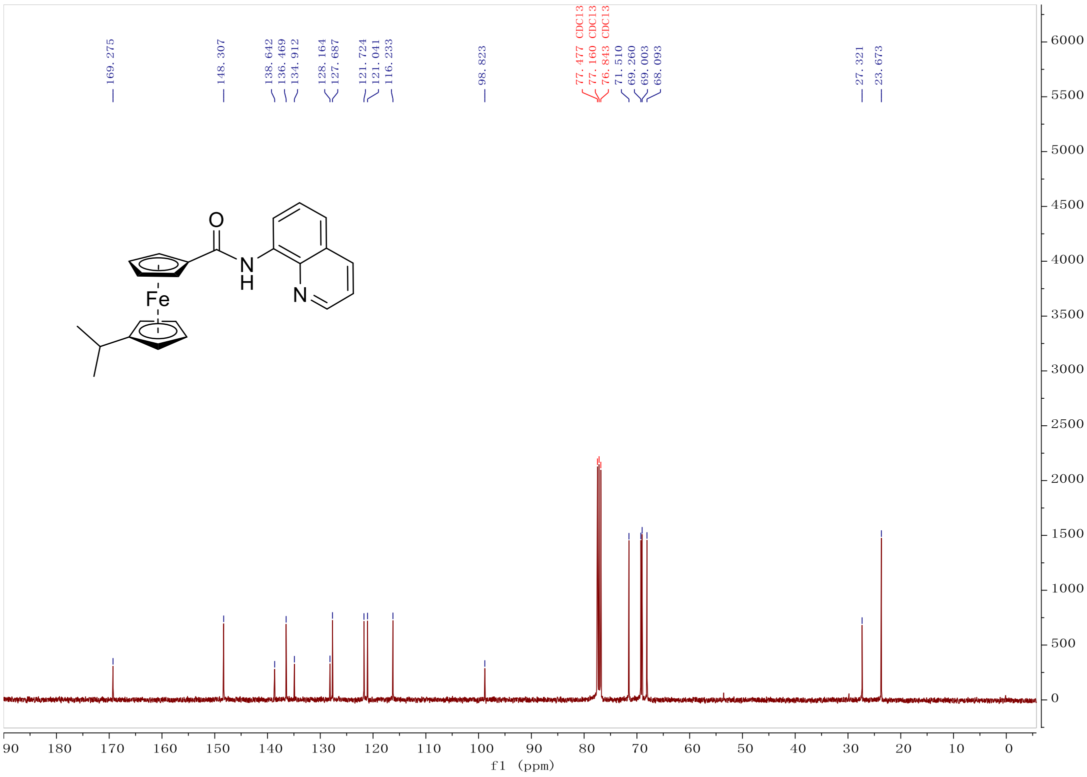

## 2d-$^{1}$H NMR

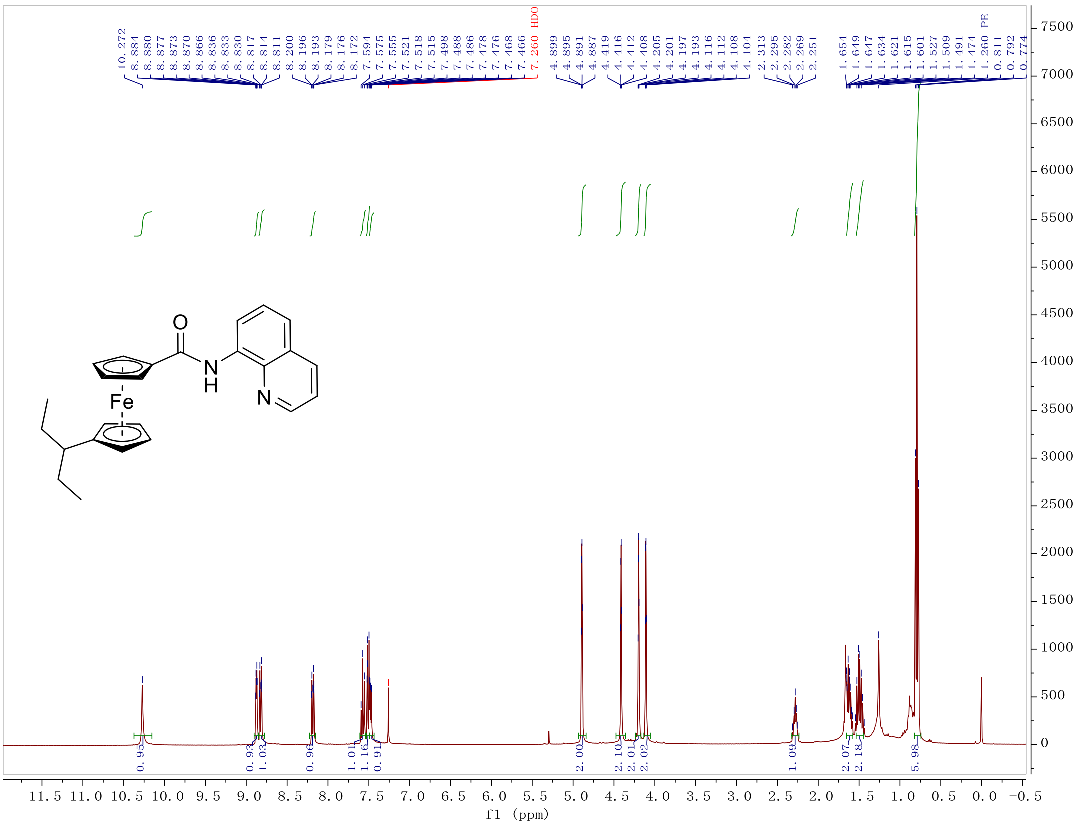

## 2d-$^{13}$C NMR

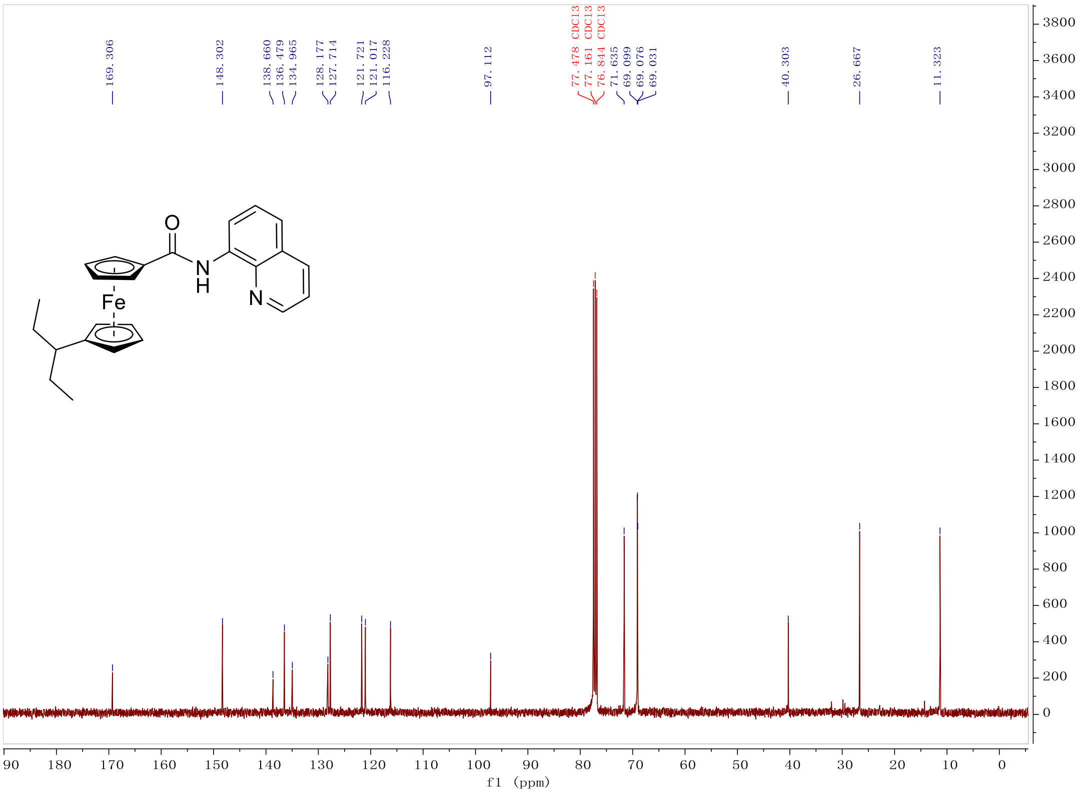

**2e-¹H NMR**

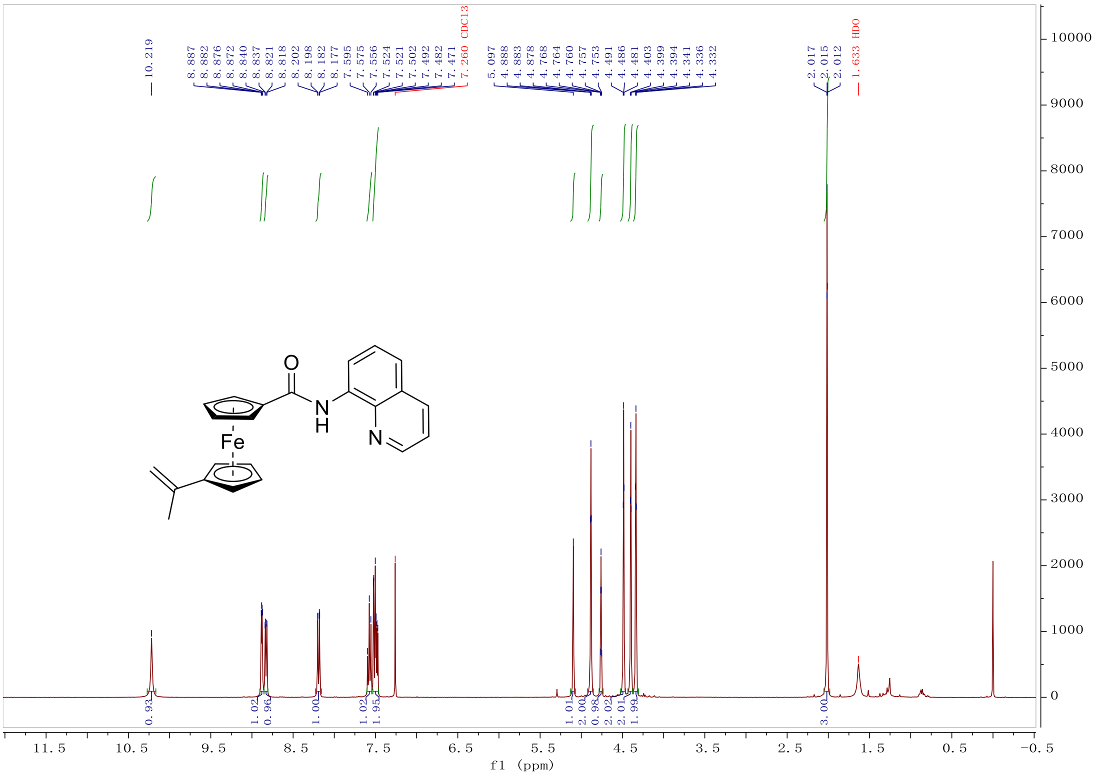


**2e-¹³C NMR**

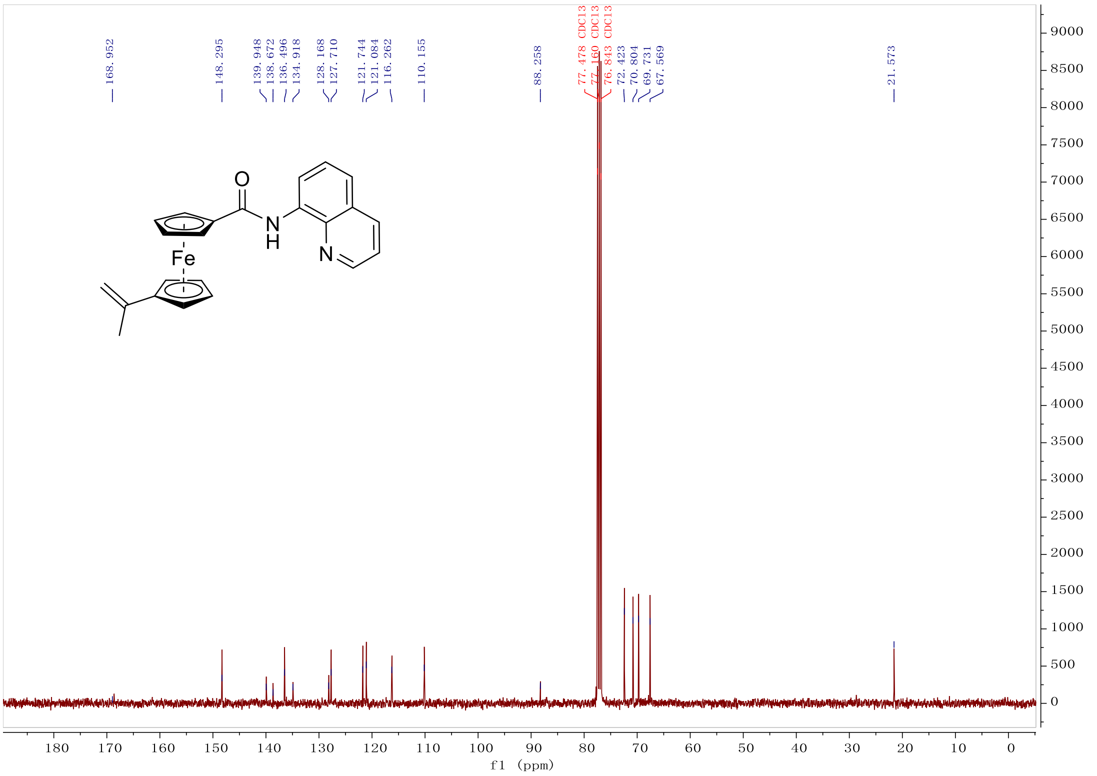

## 2f-¹H NMR

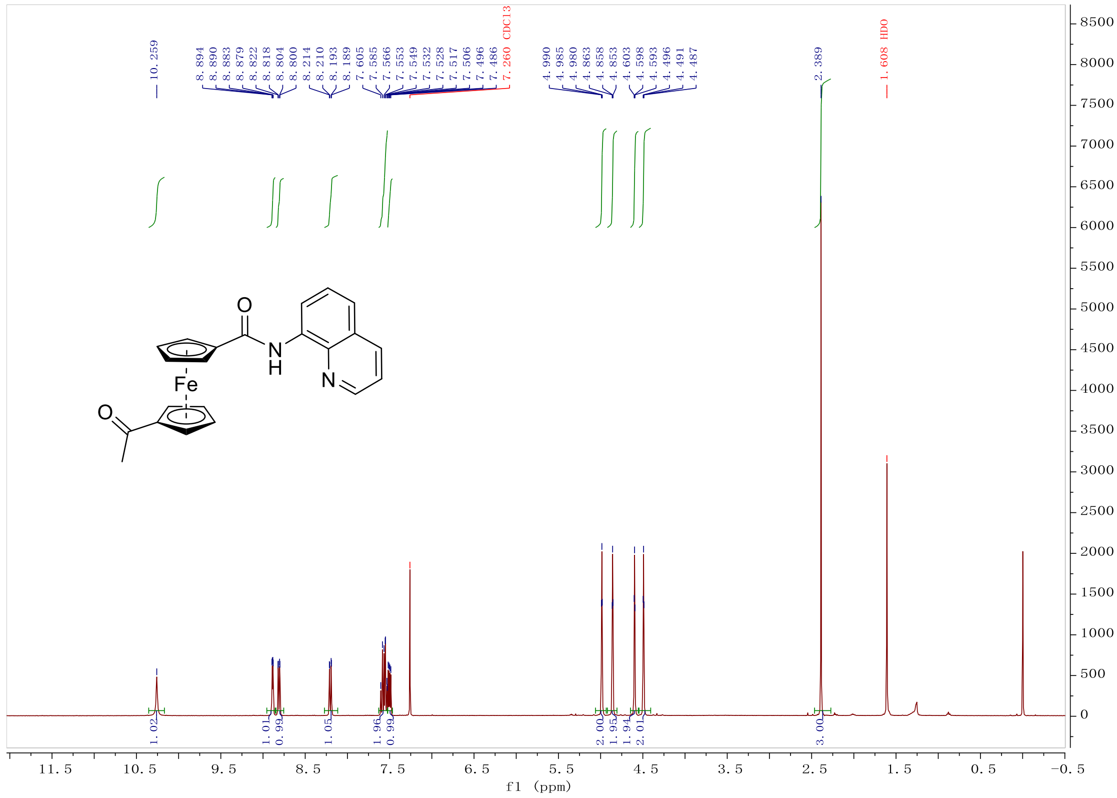

## 2f-¹³C NMR

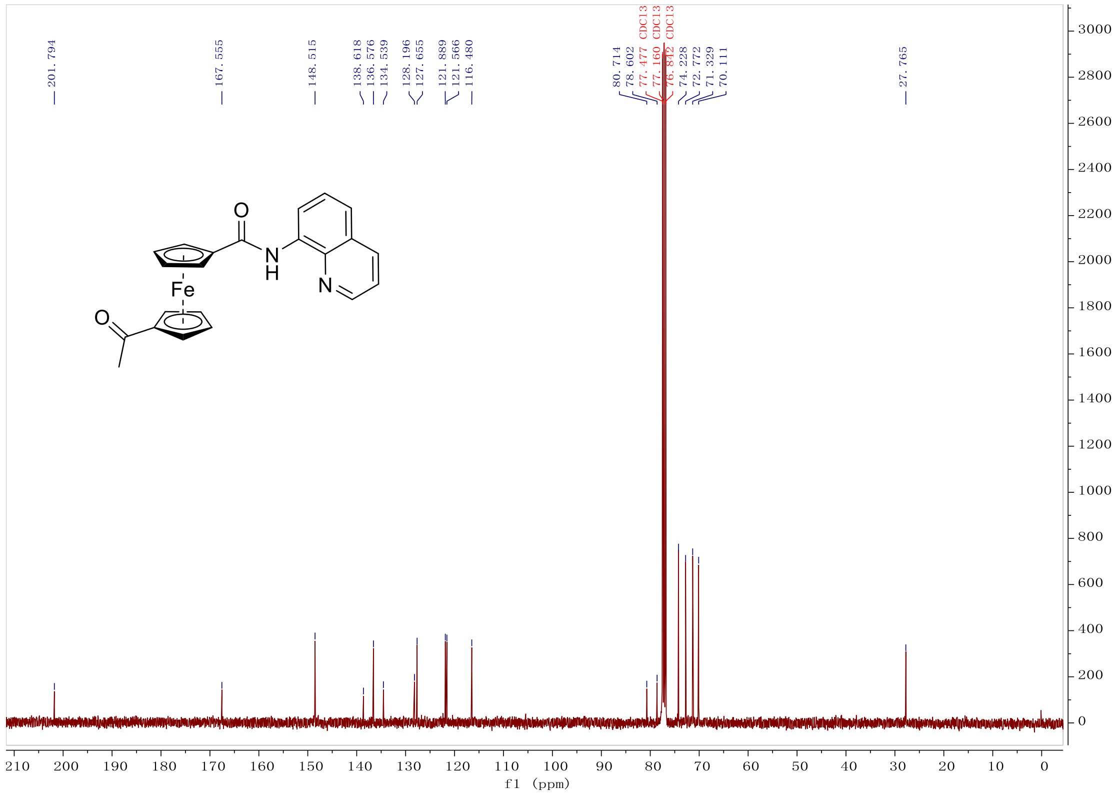



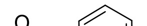

## 2g-$^{1}$H NMR

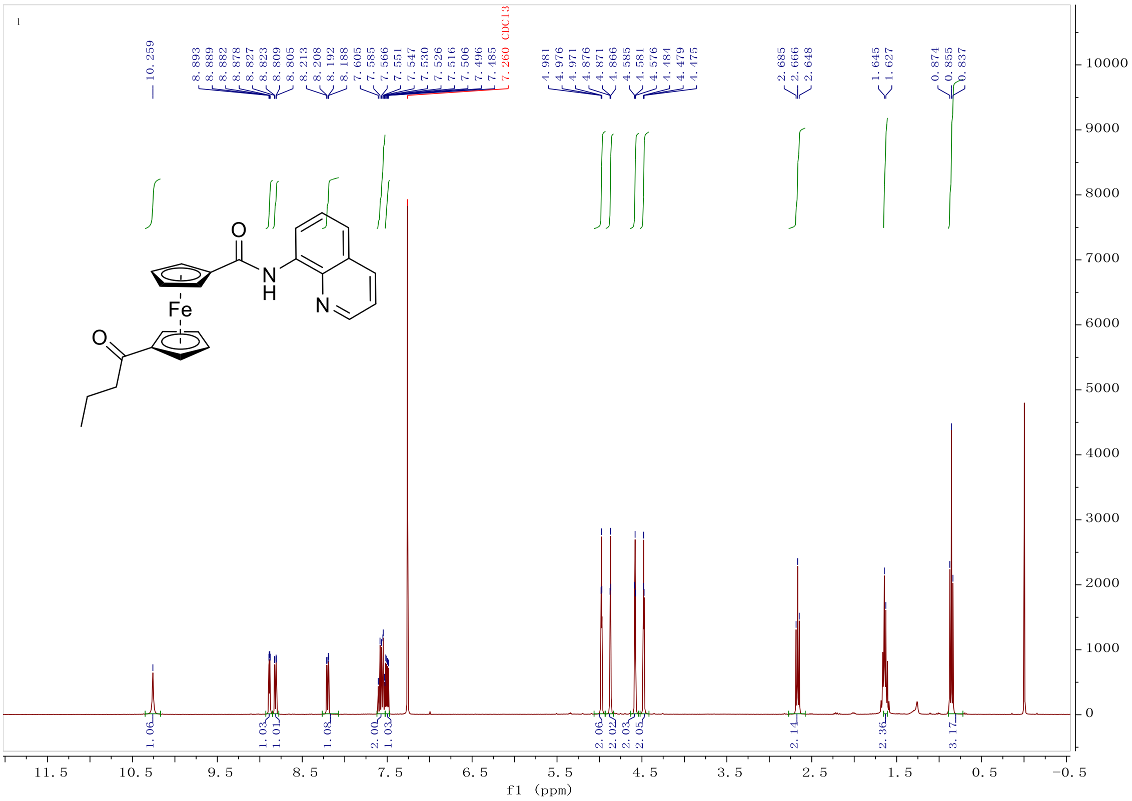

## 2g-$^{13}$C NMR

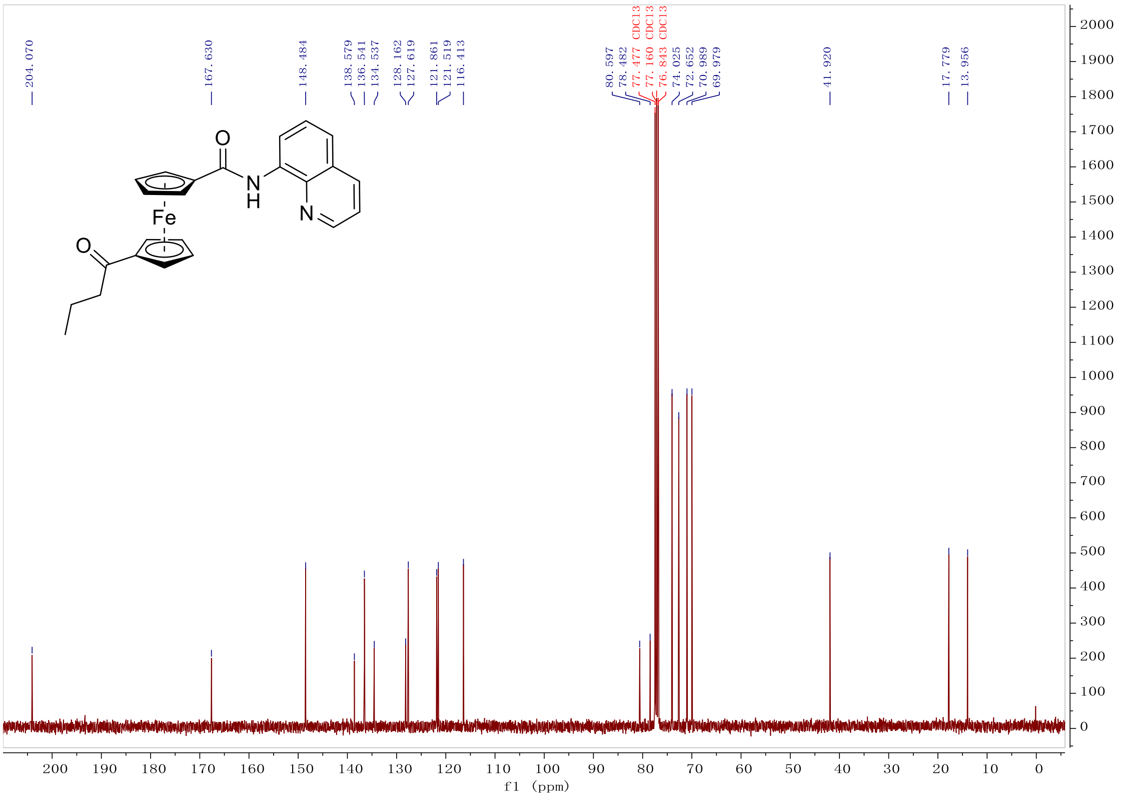

**2h-$^{1}$H NMR**

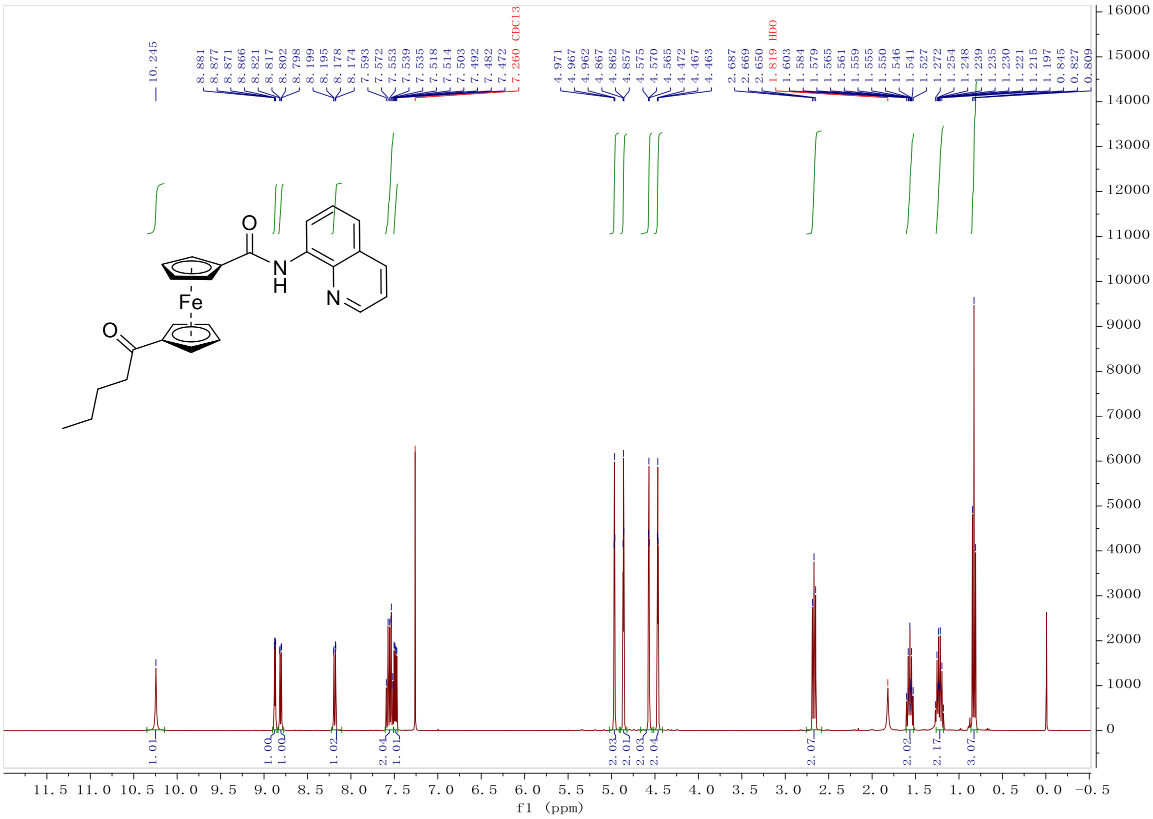

**2h-$^{13}$C NMR**

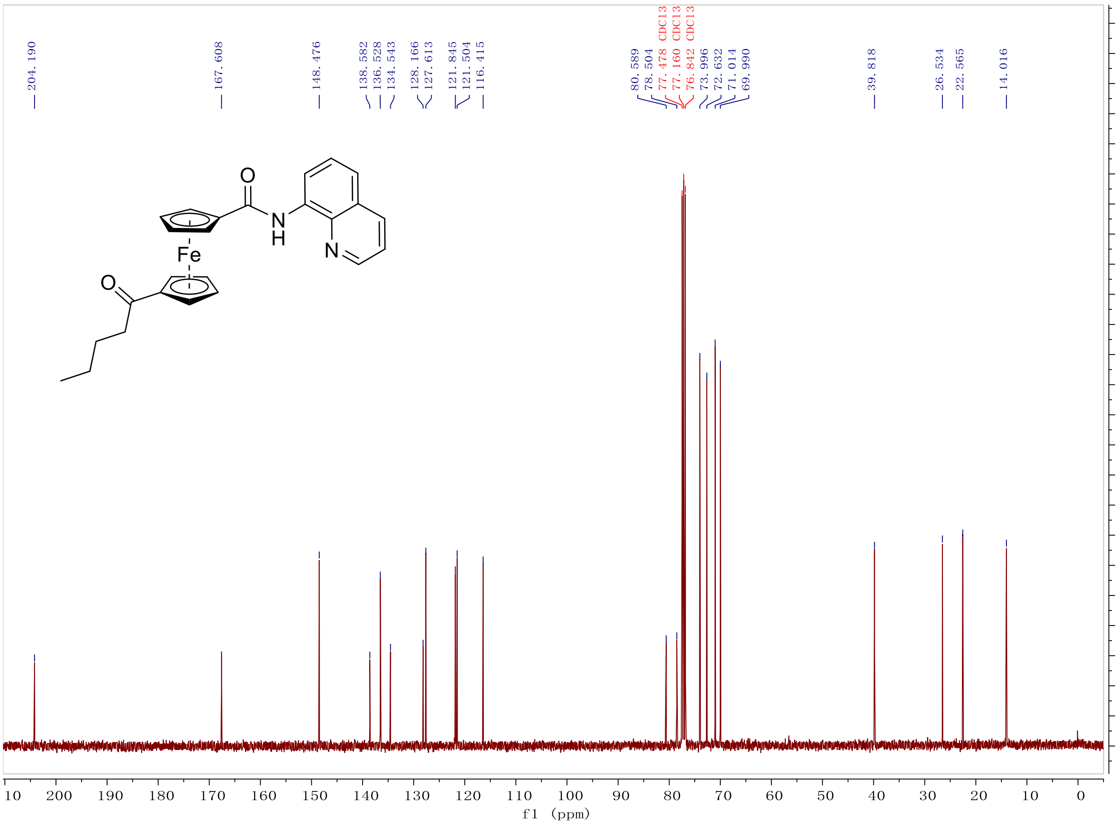

## 2i-$^{1}$H NMR

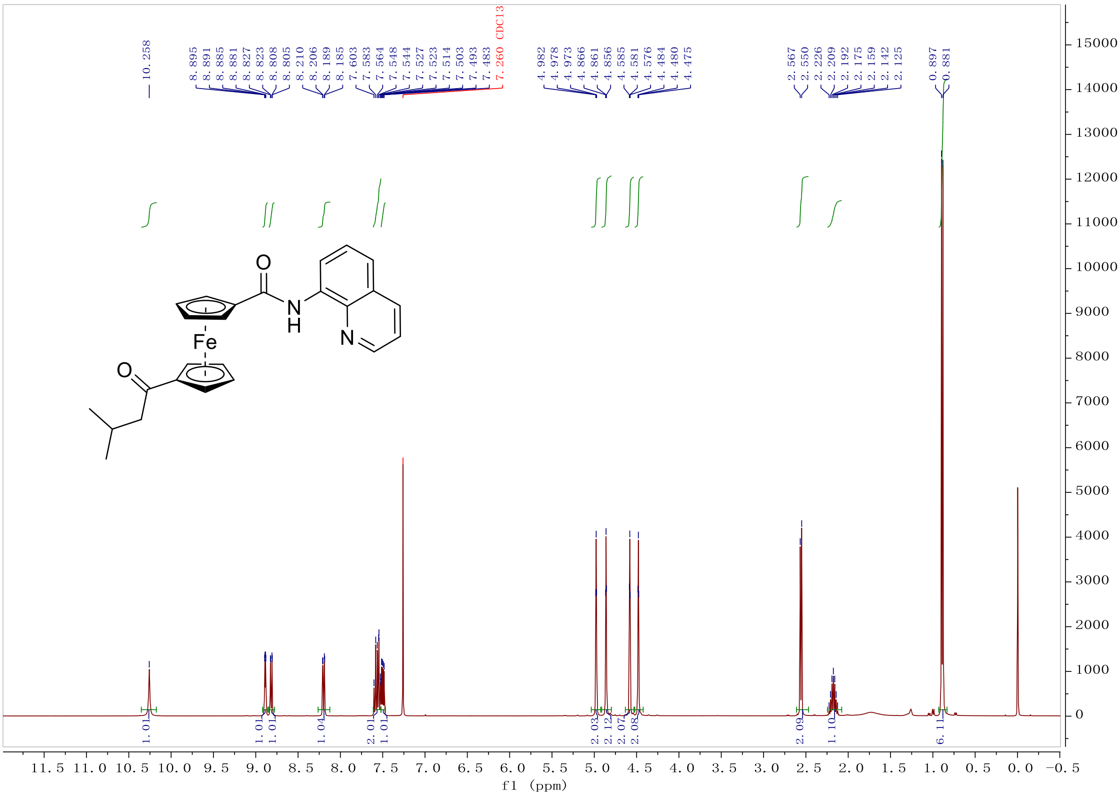


## 2i-$^{13}$C NMR

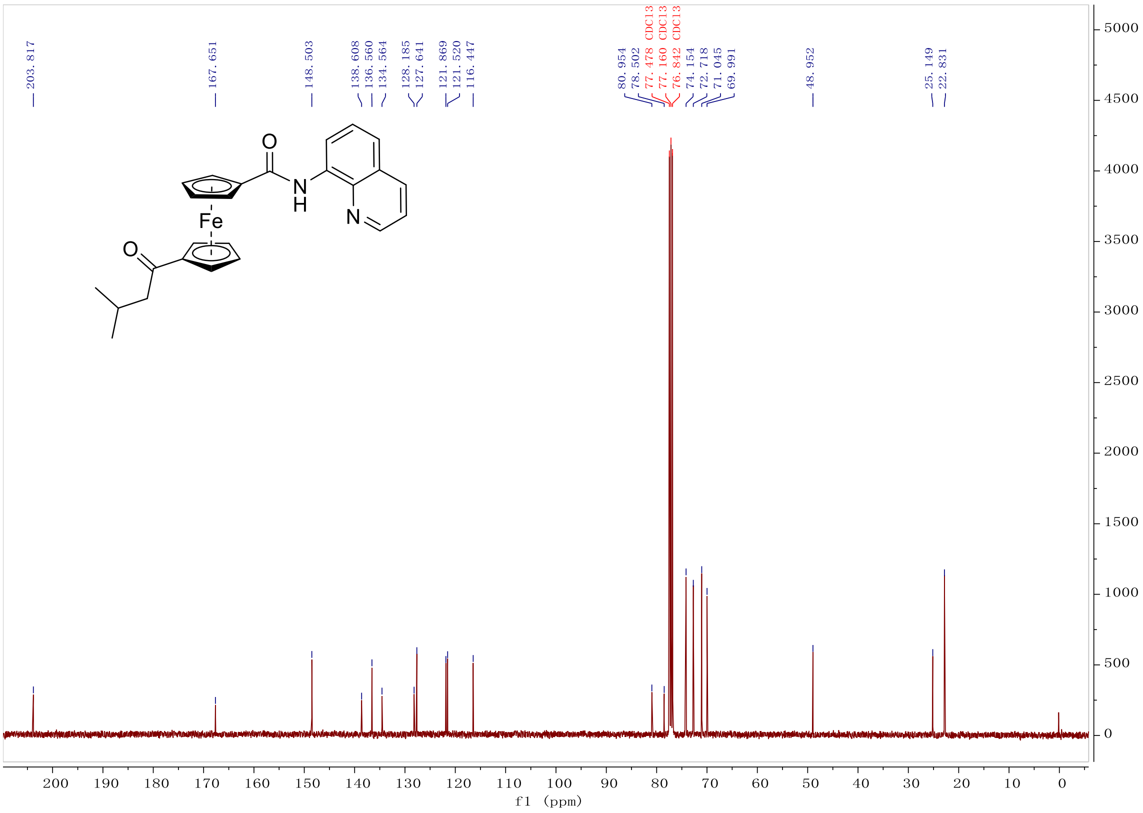

## 2j-$^1$H NMR

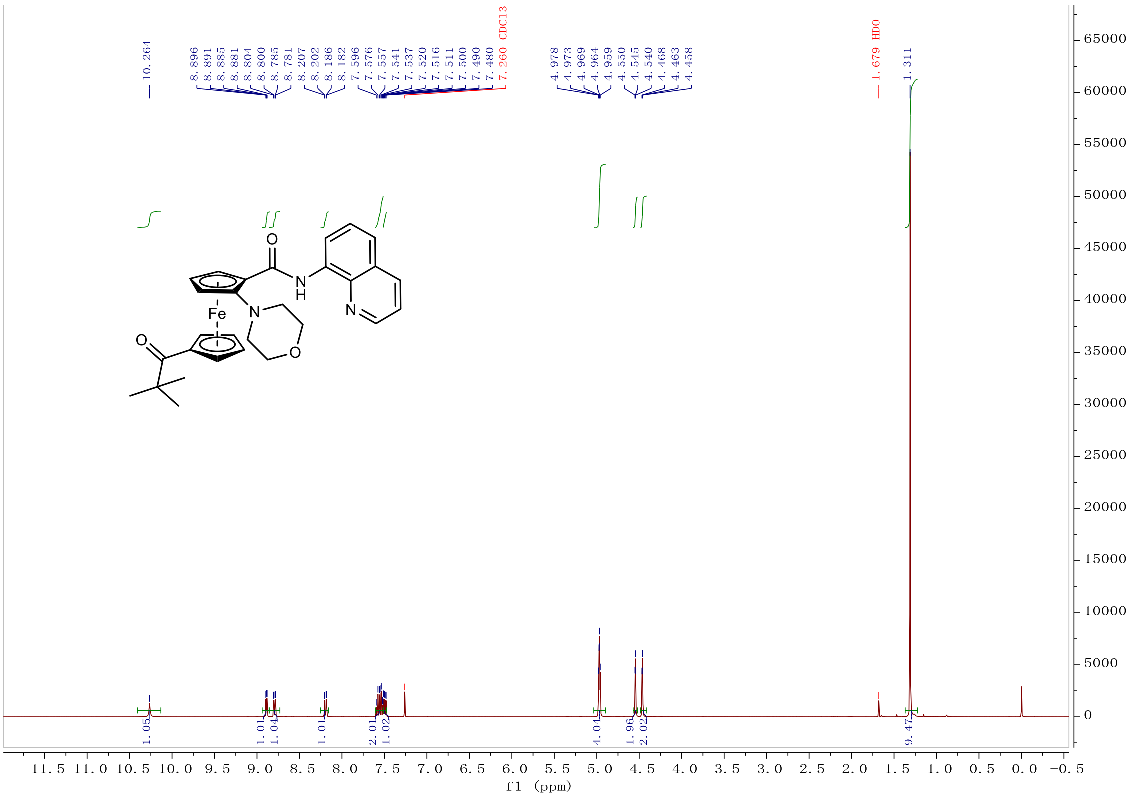

## 2j-$^{13}$C NMR

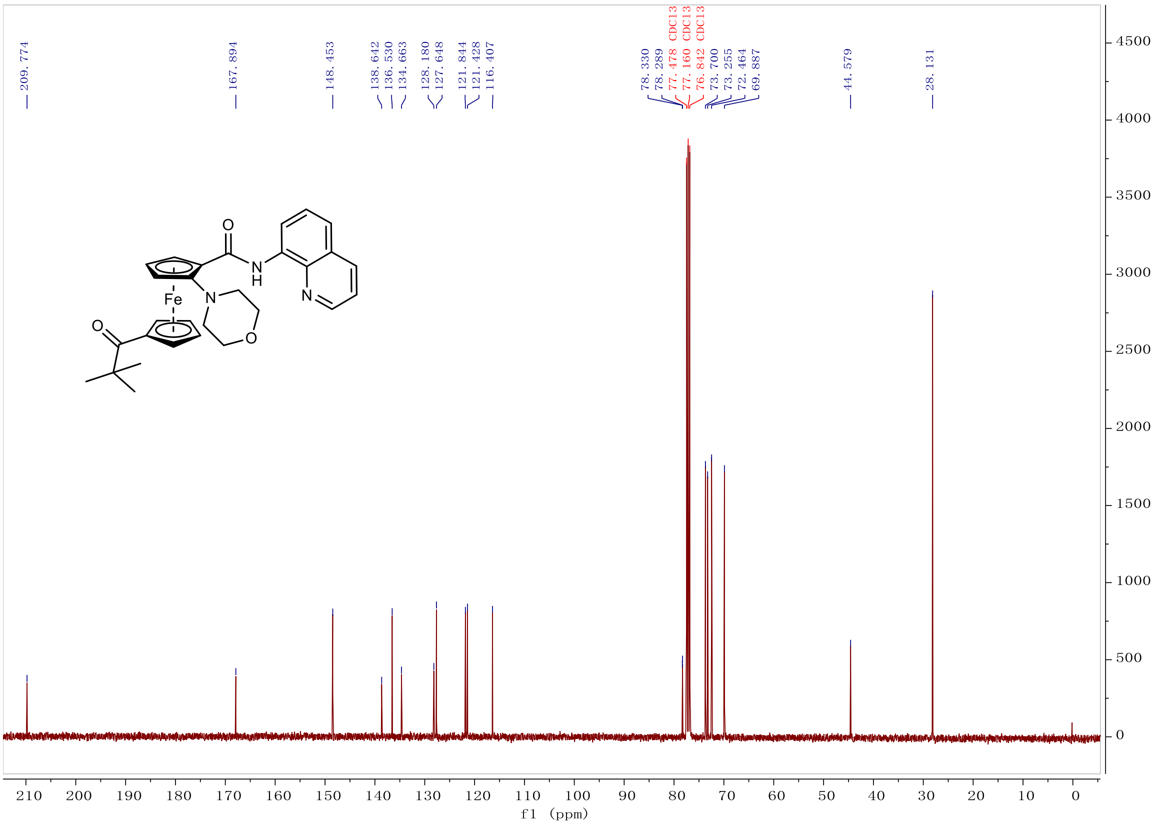

## 2k-$^{1}$H NMR

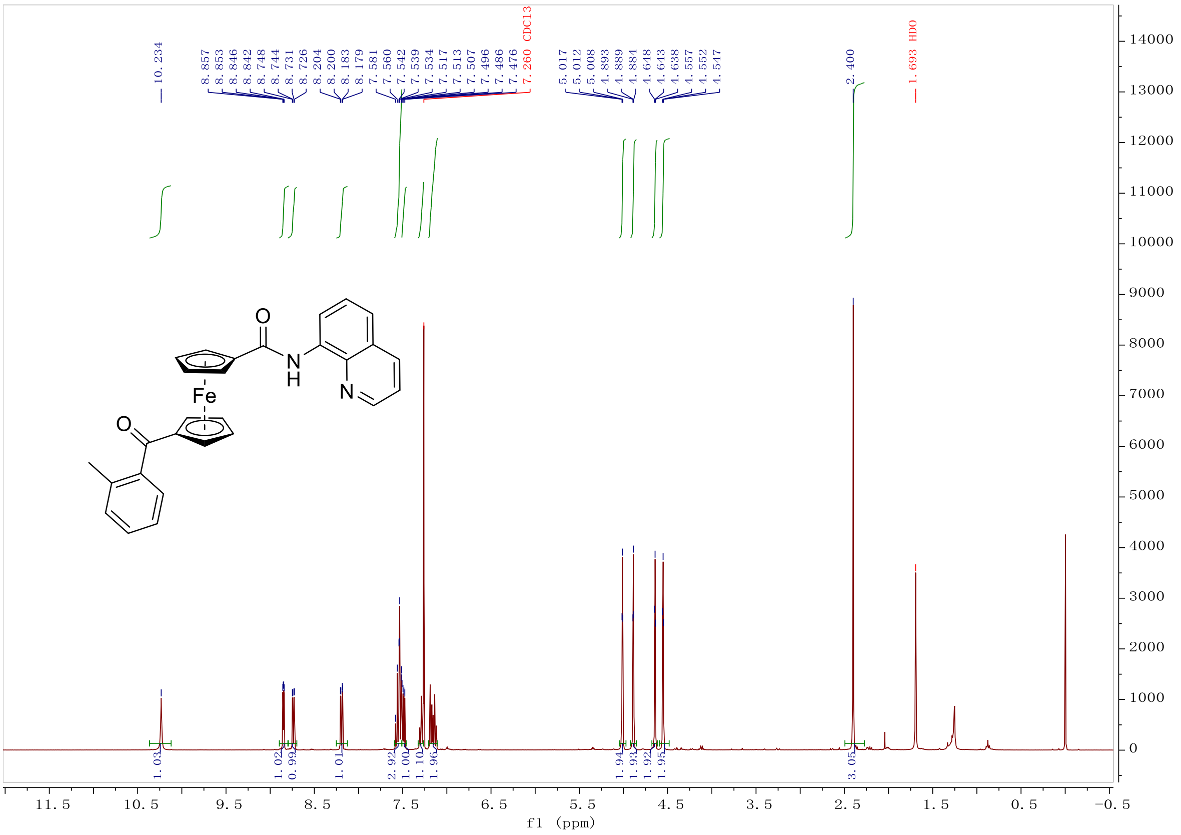

## 2k-$^{13}$C NMR

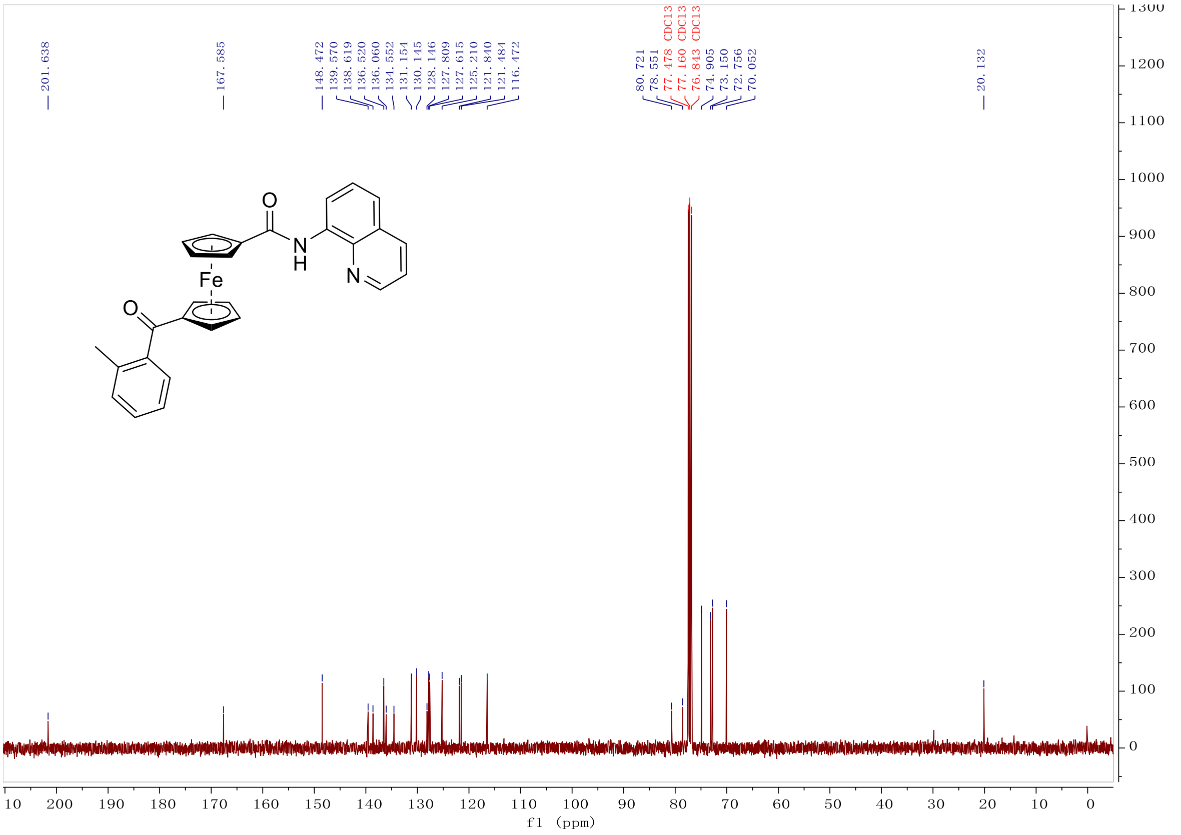

**2l-$^{1}$H NMR**

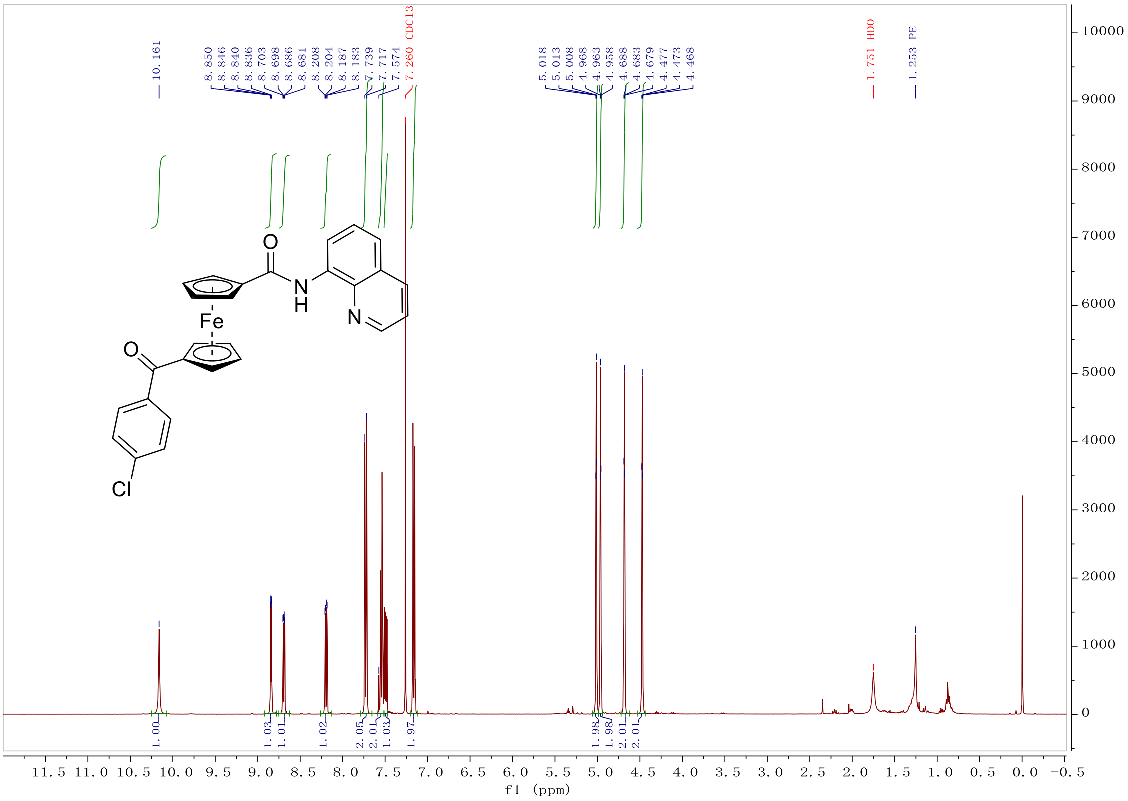

**2l-$^{13}$C NMR**

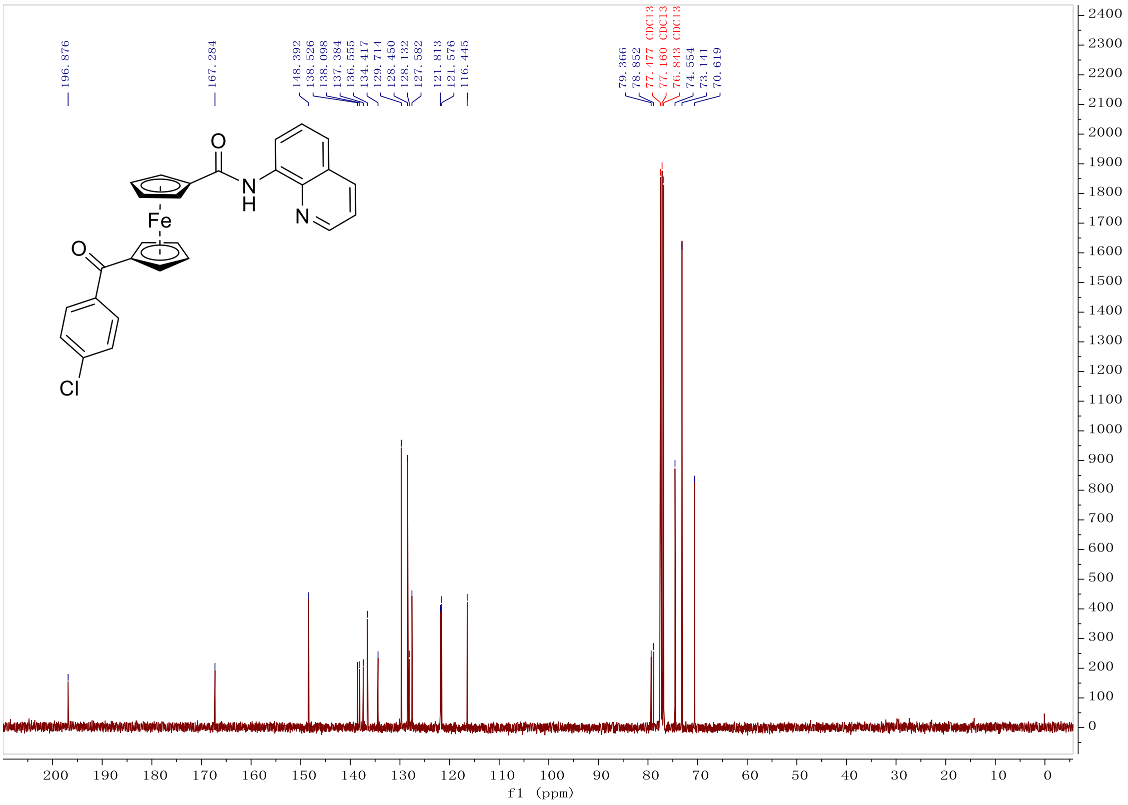

**2m-¹H NMR**

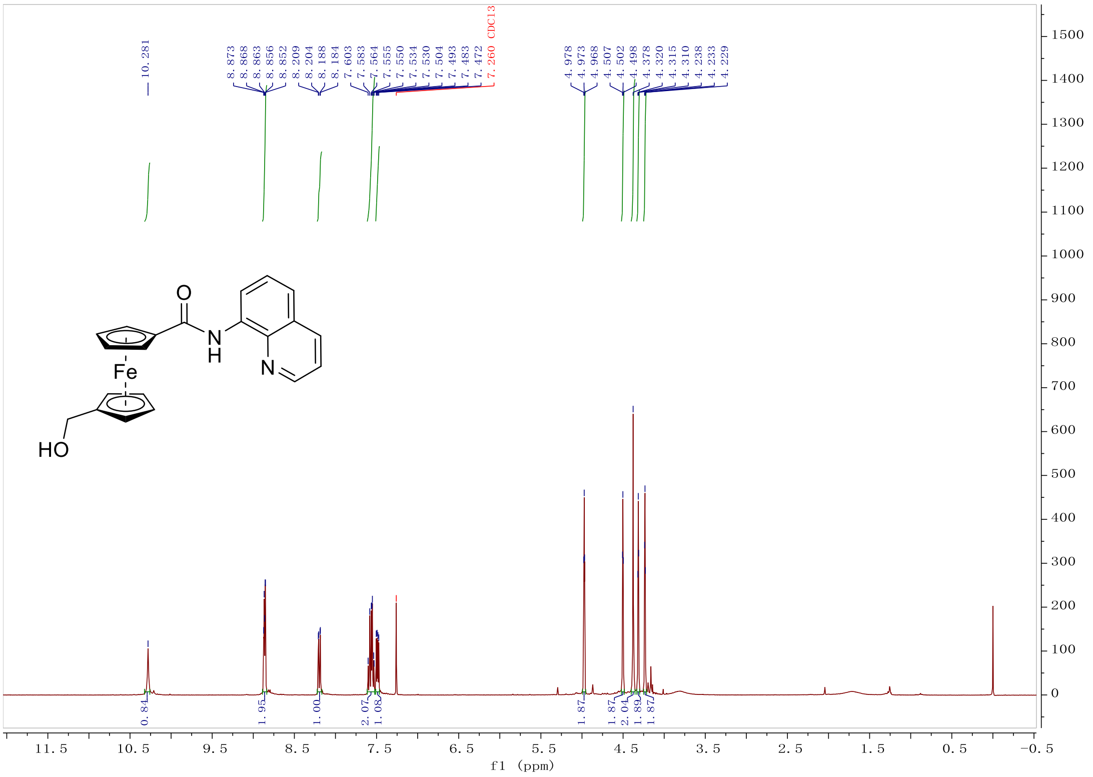

**2m-¹³C NMR**

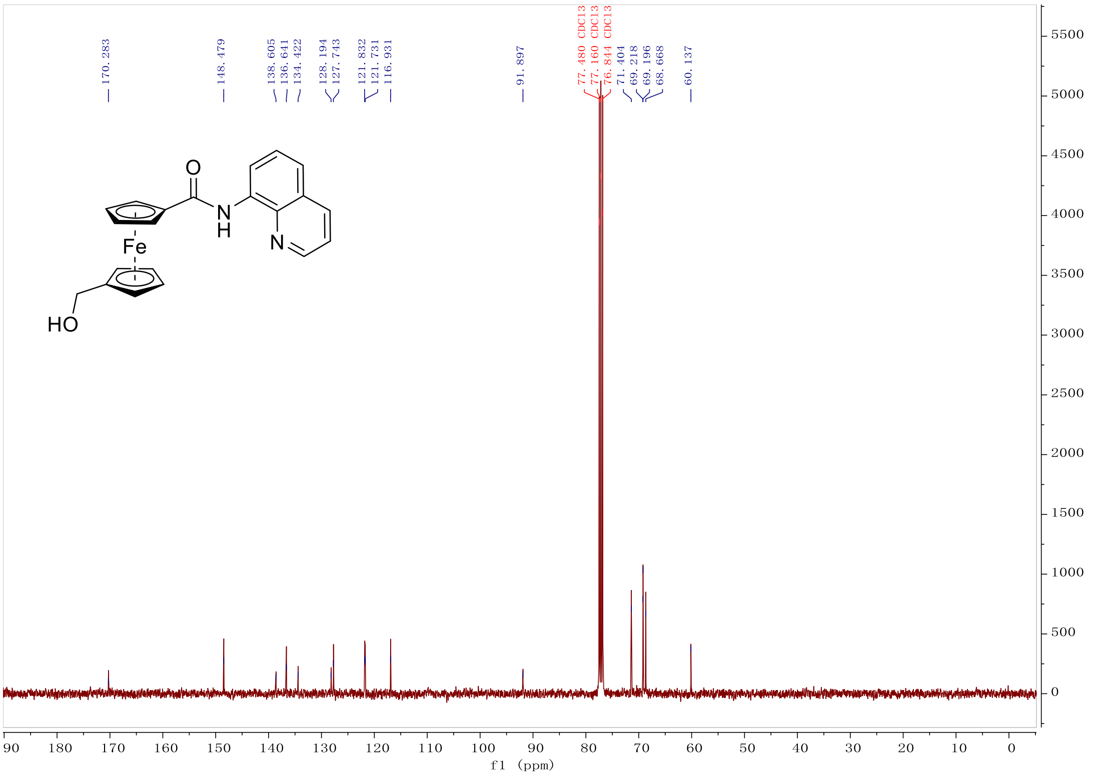

## 3b-¹H NMR

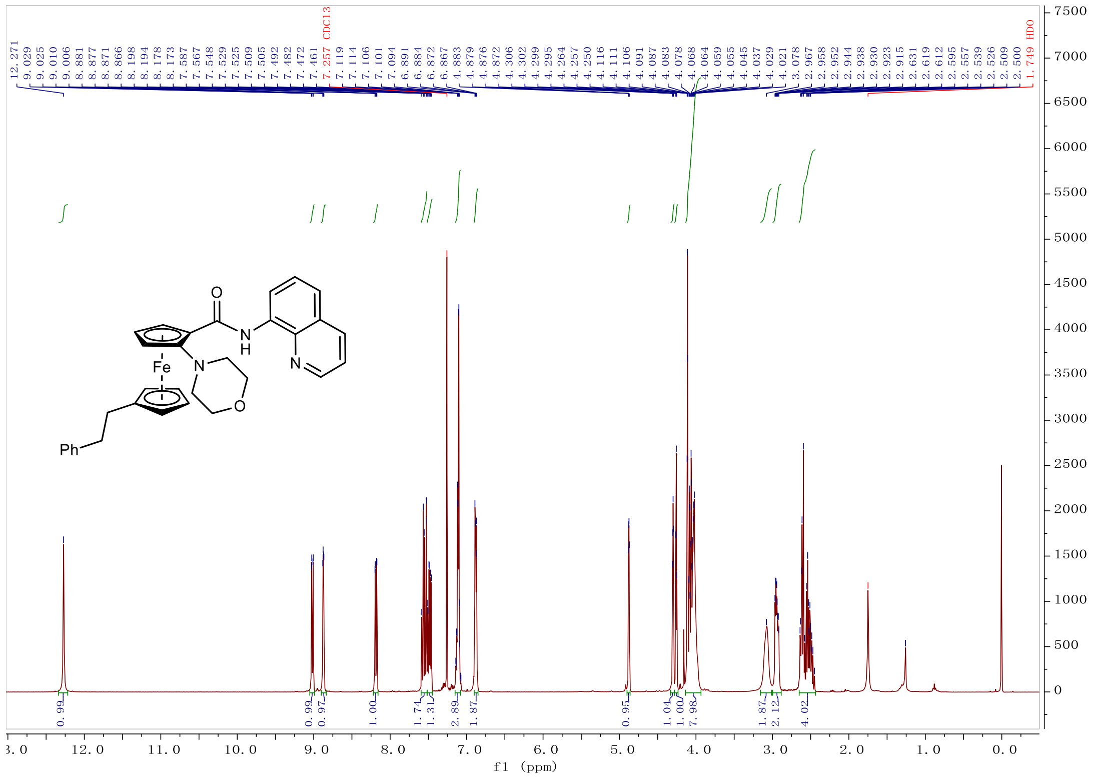


## 3b-¹³C NMR

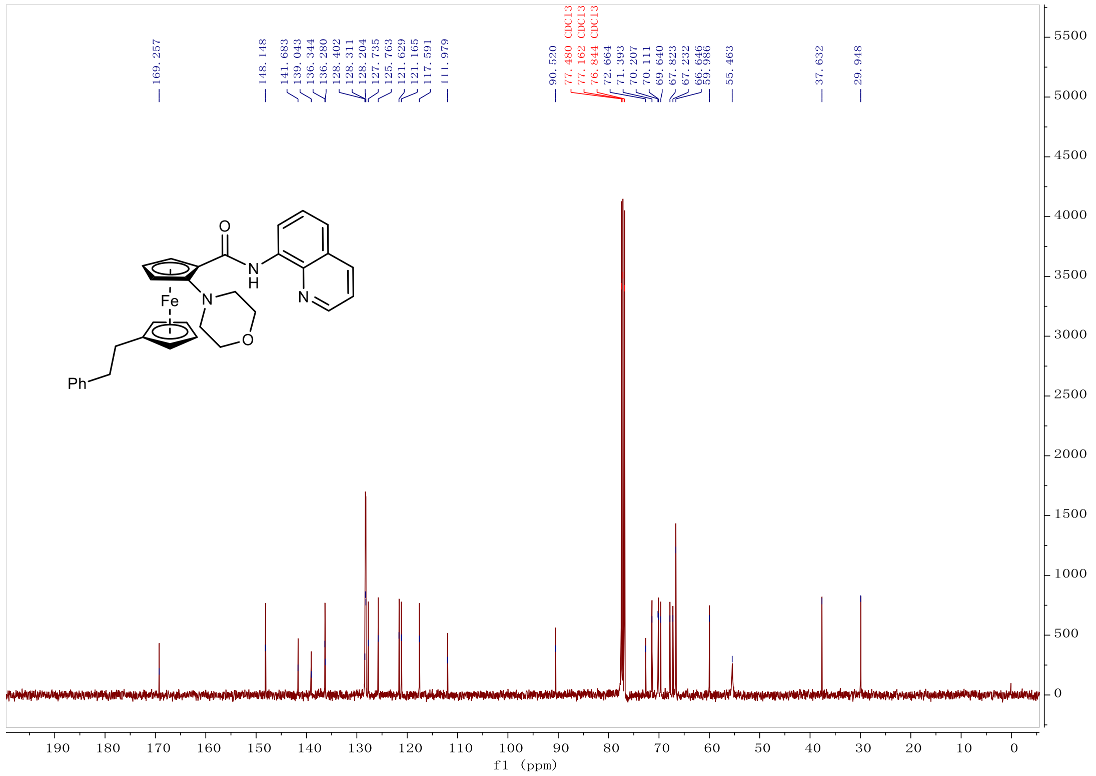

## 3c-$^{1}$H NMR

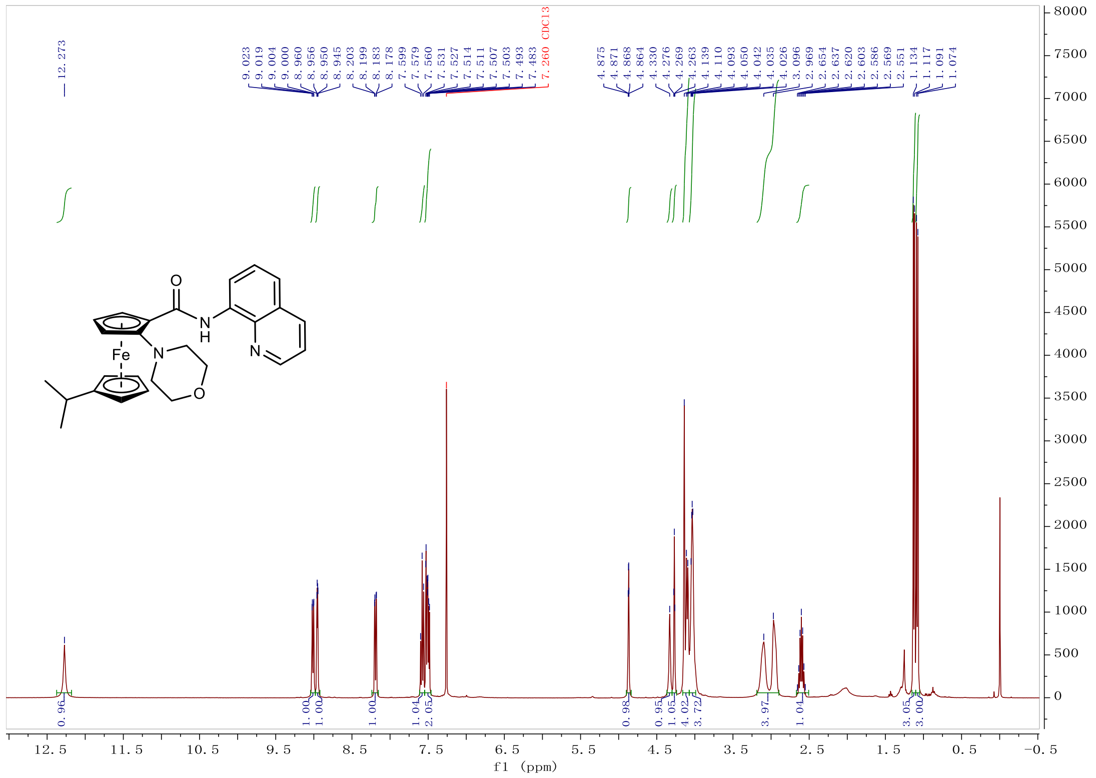

## 3c-$^{13}$C NMR

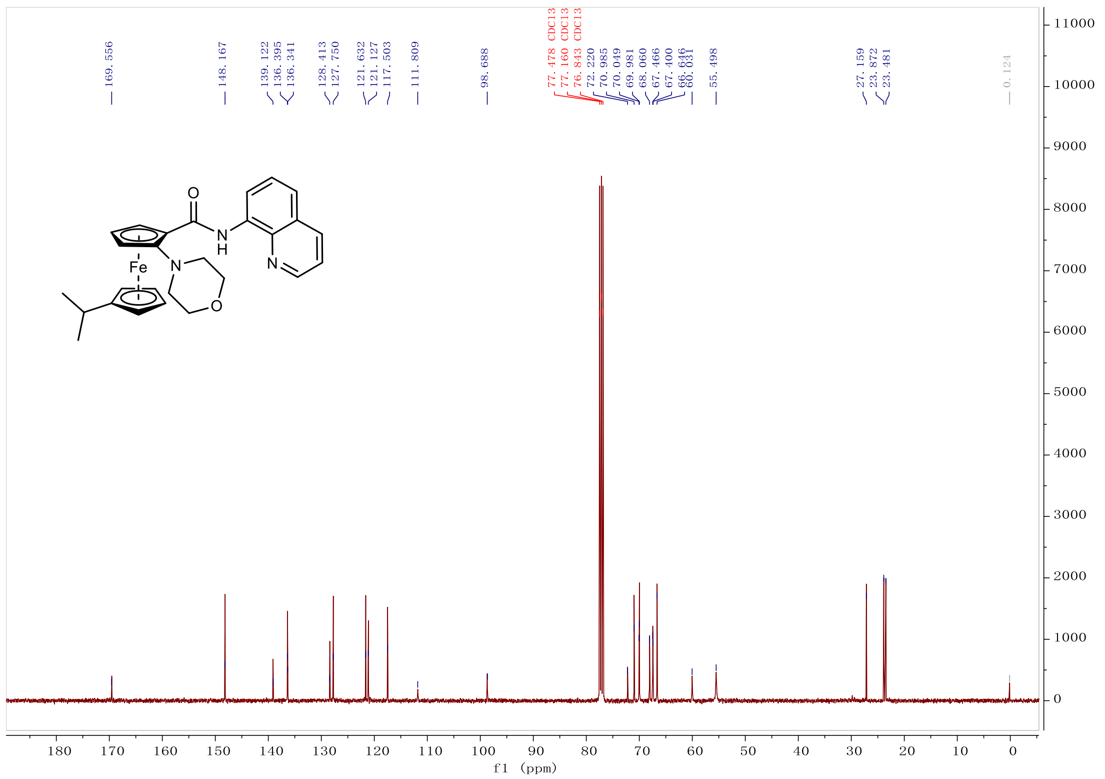

**3d-$^{1}$H NMR**

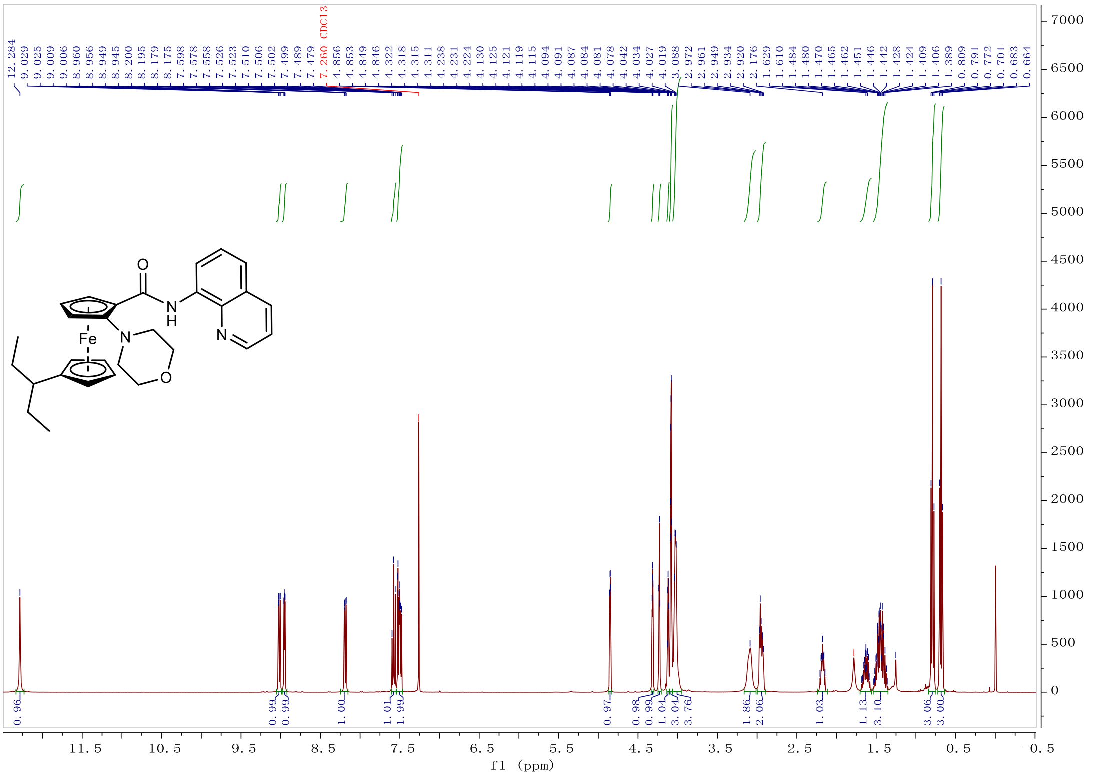

**3d-$^{13}$C NMR**

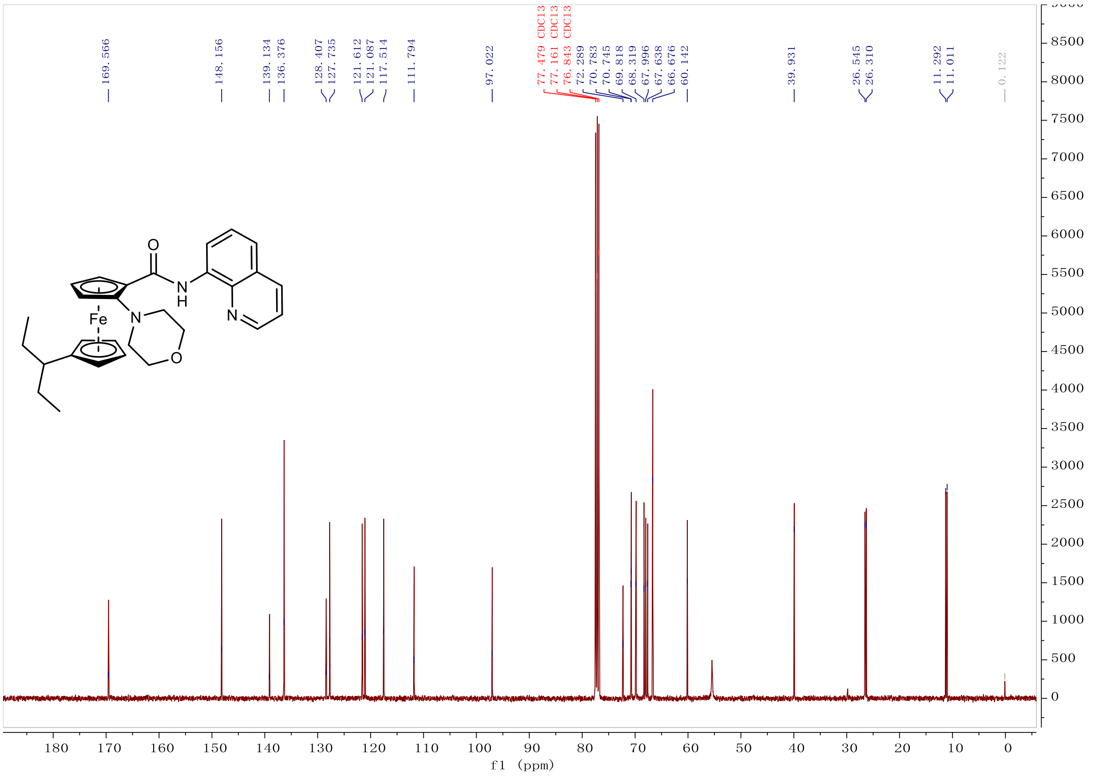

**3e-$^{1}$H NMR**

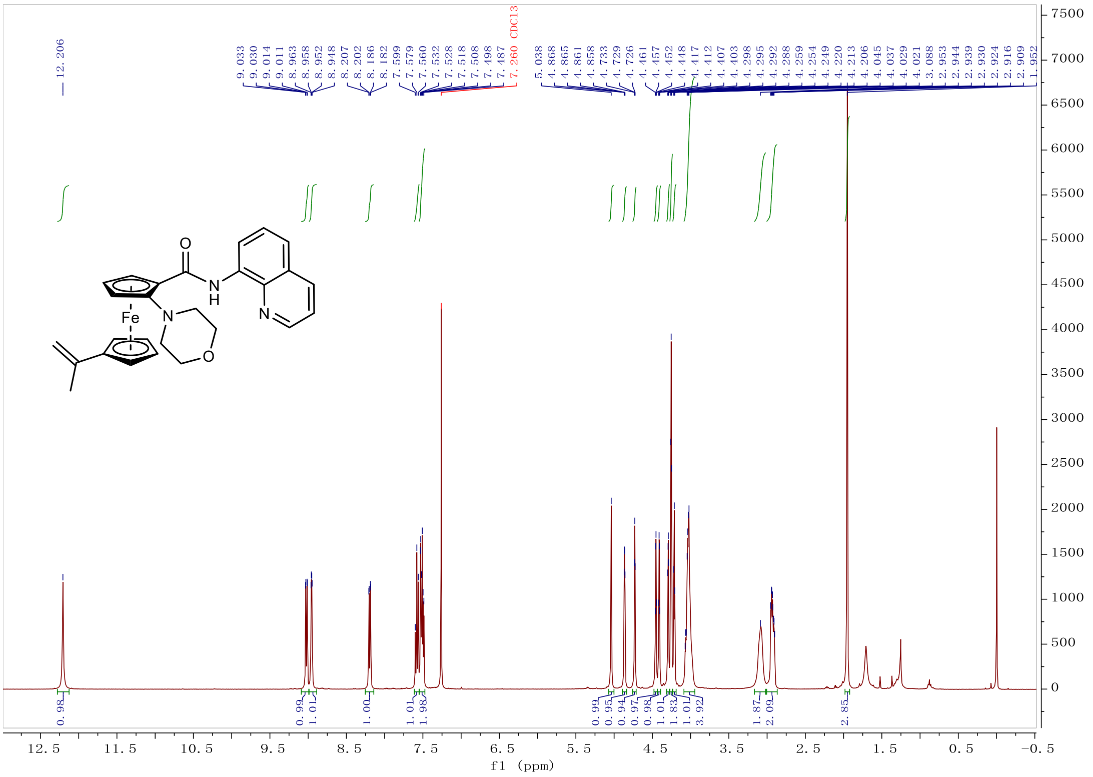

**3e-$^{13}$C NMR**

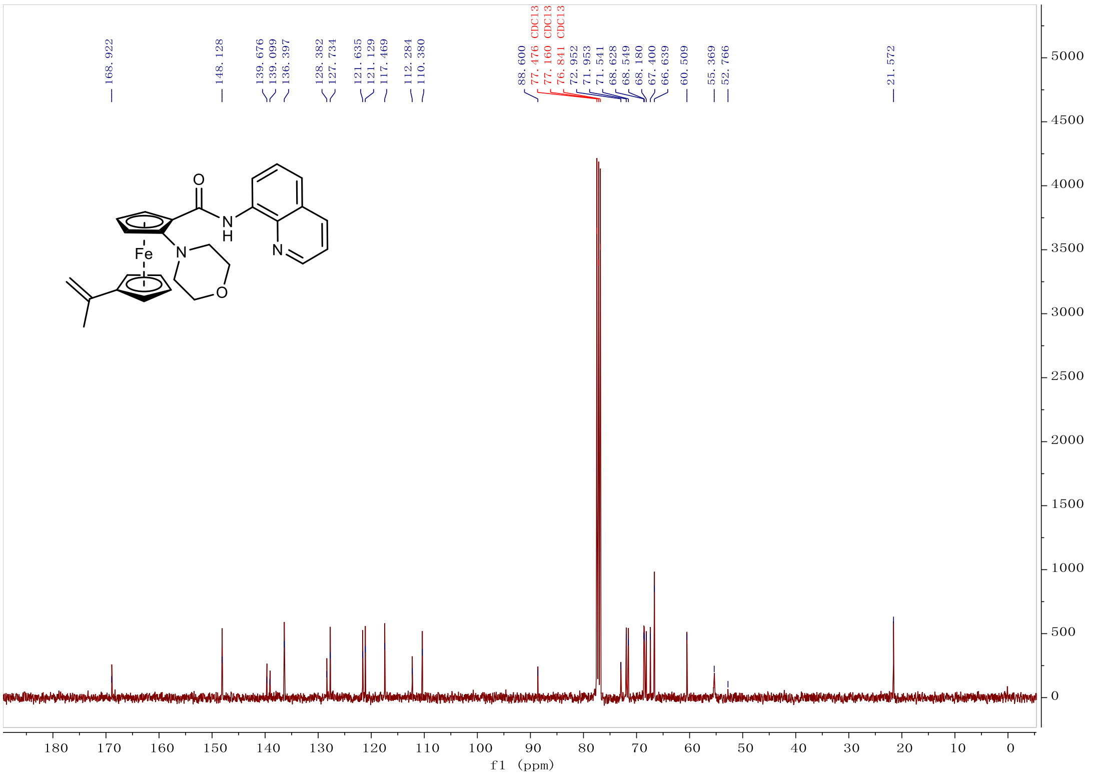

## 3f-$^{1}$H NMR

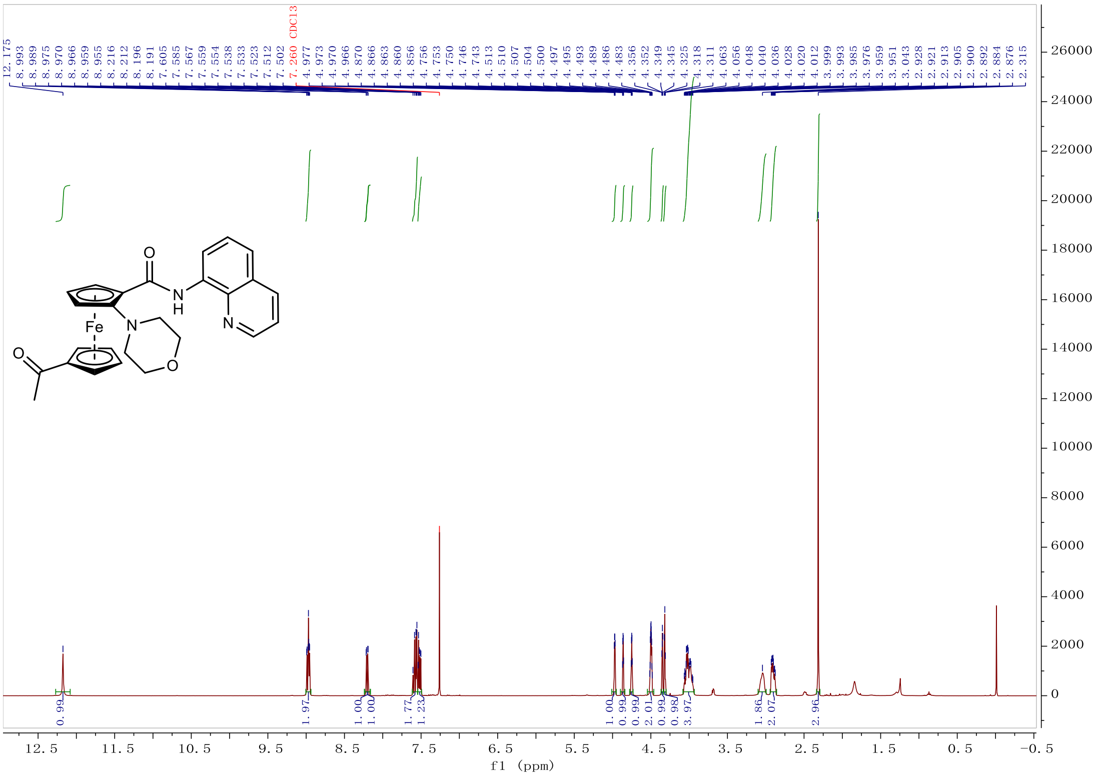


## 3f-$^{13}$C NMR

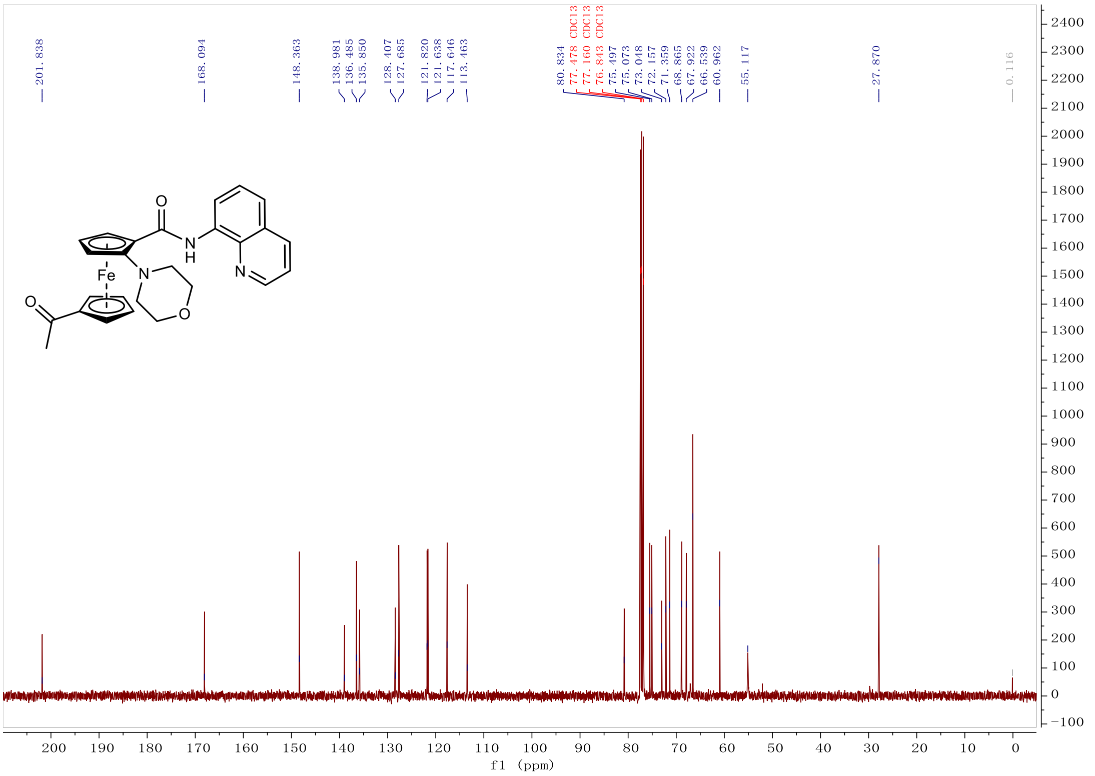

## 3g-$^{1}$H NMR

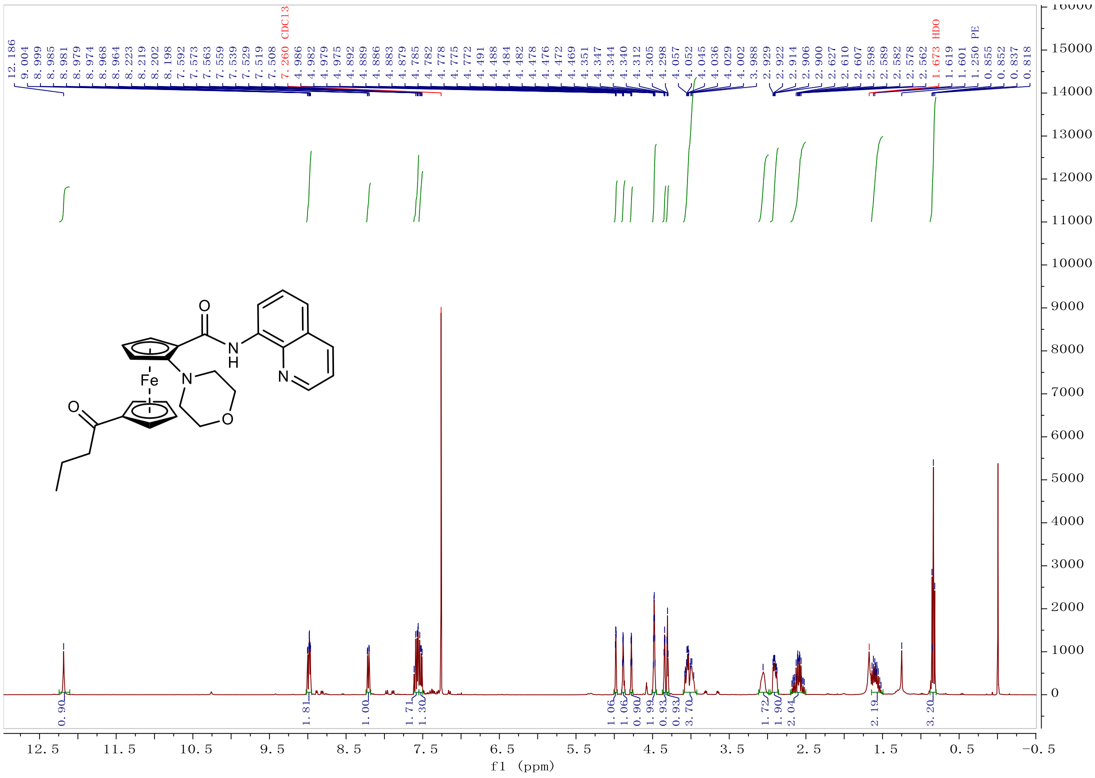


## 3g-$^{13}$C NMR

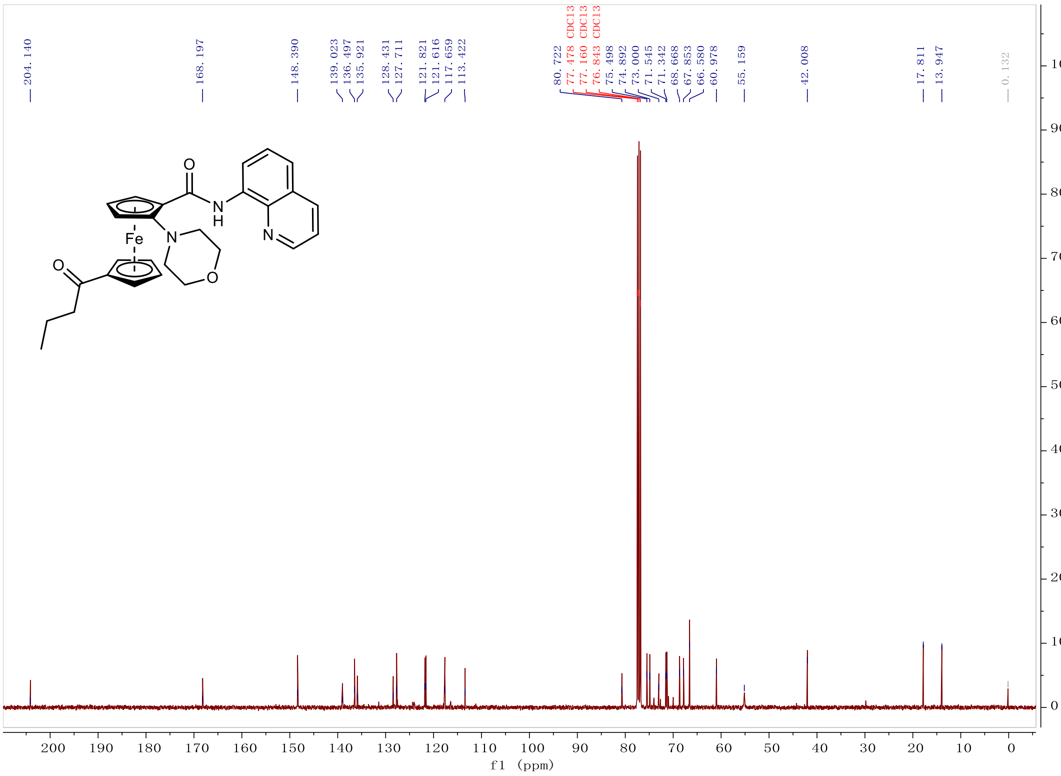

## 3h-¹H NMR

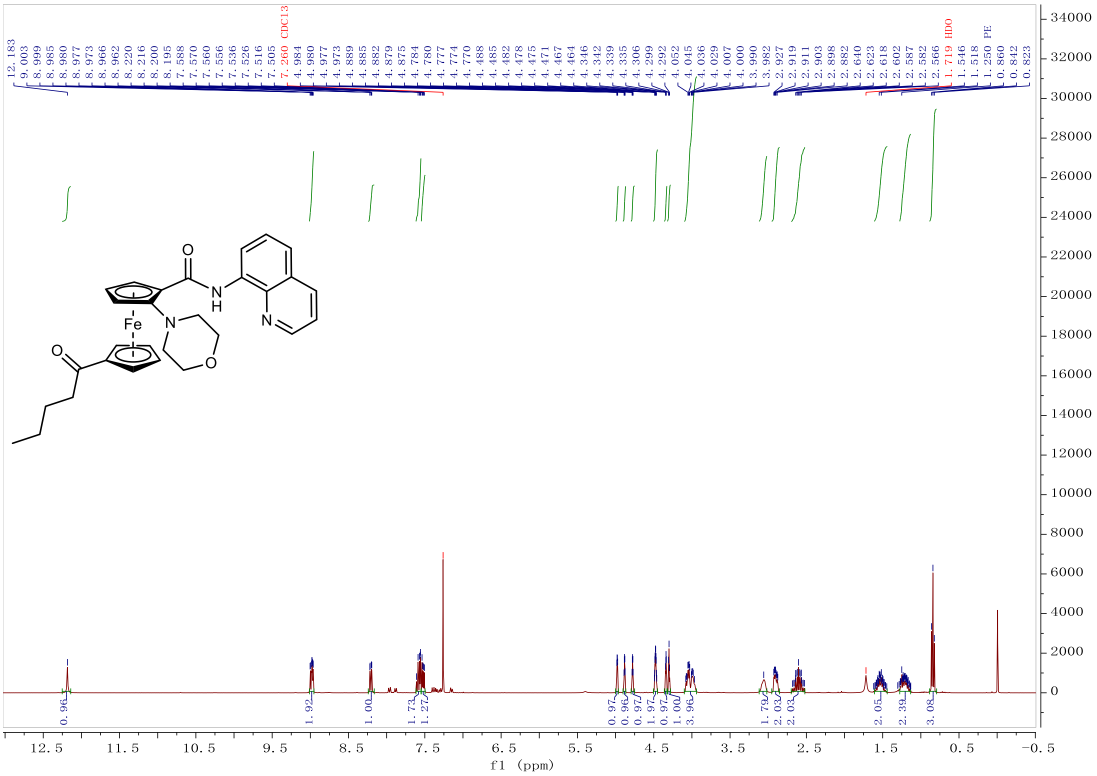

## 3h-¹³C NMR

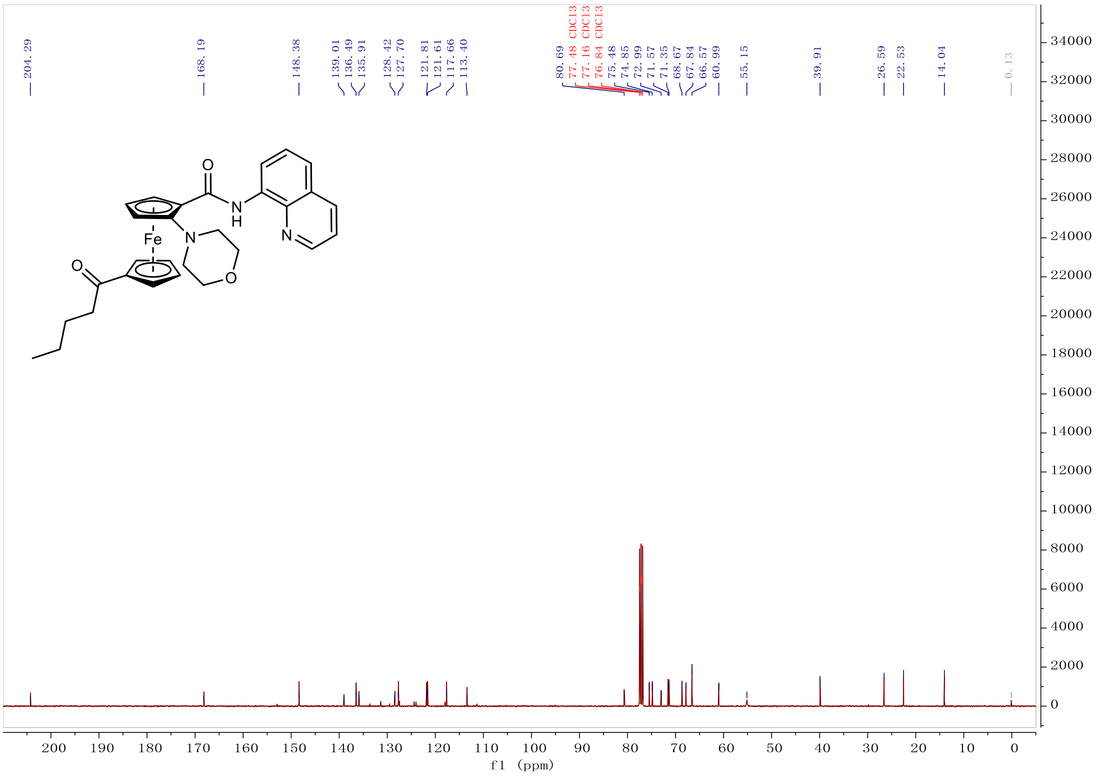

## 3i-$^{1}$H NMR

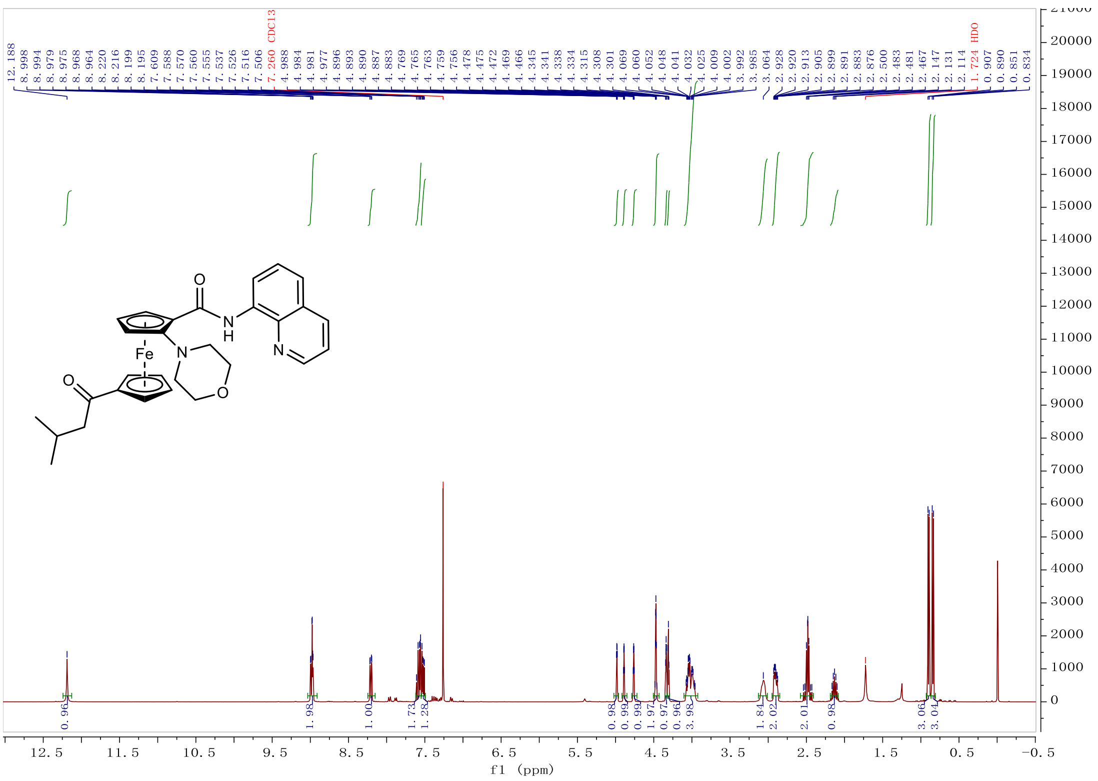

## 3i-$^{13}$C NMR

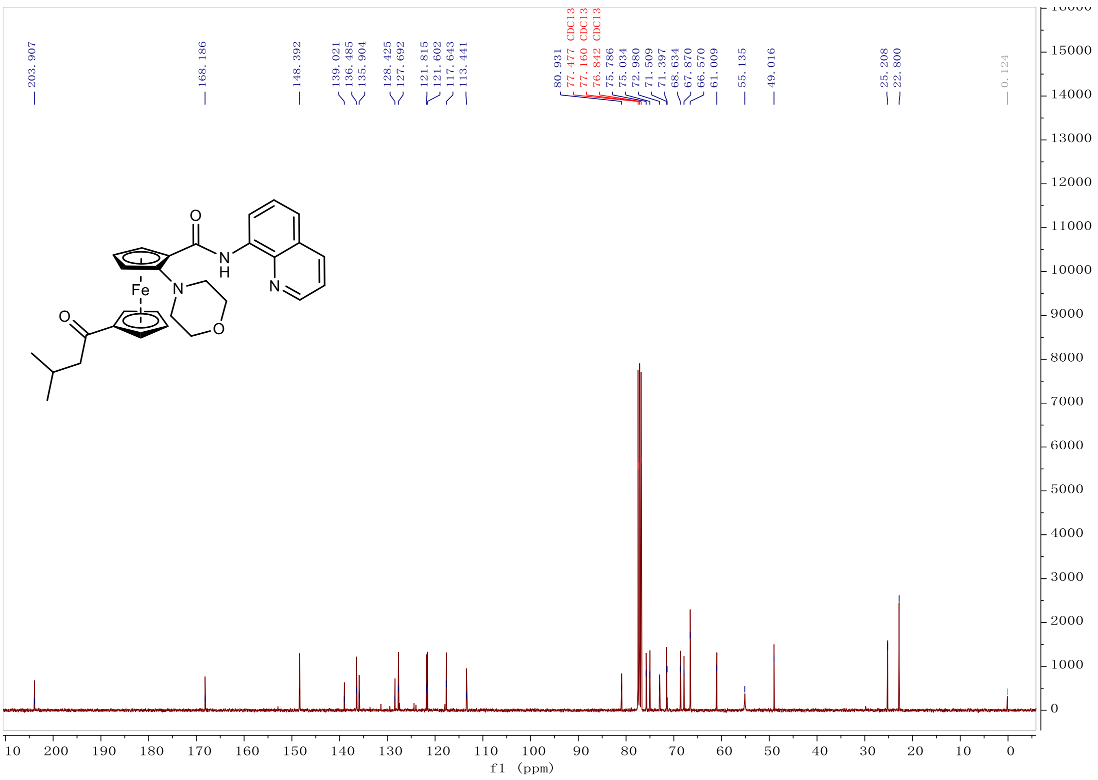

## 3j-$^{1}$H NMR

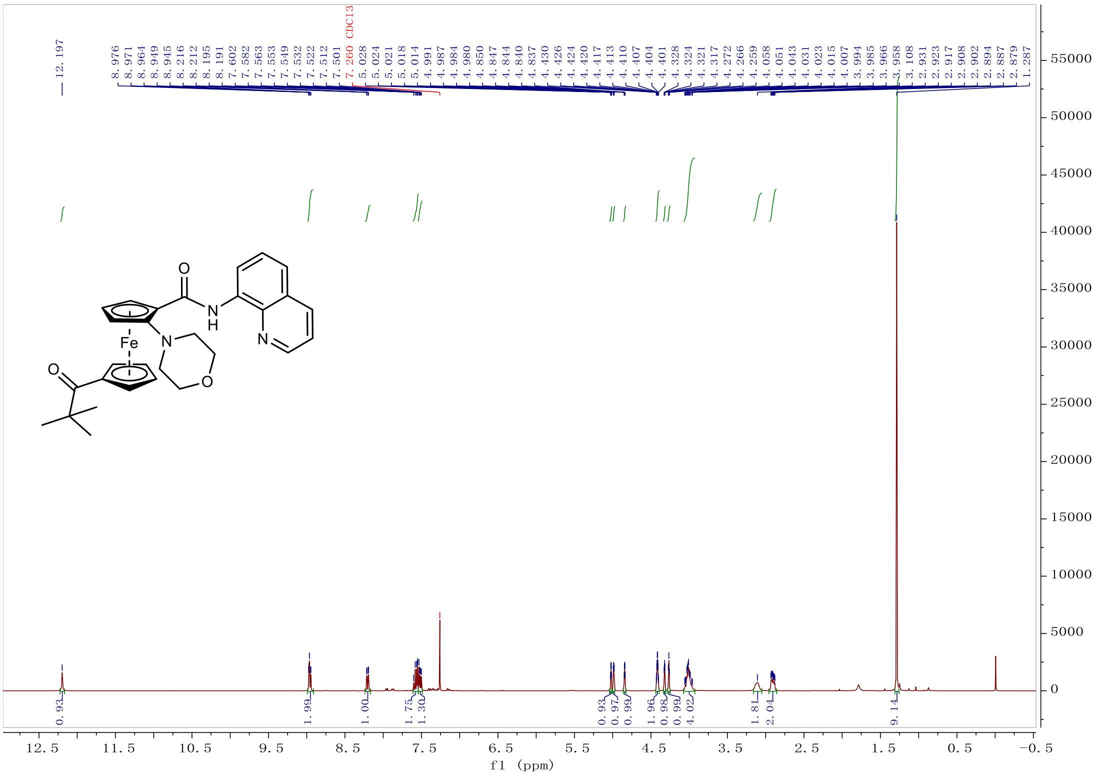

## 3j-$^{13}$C NMR

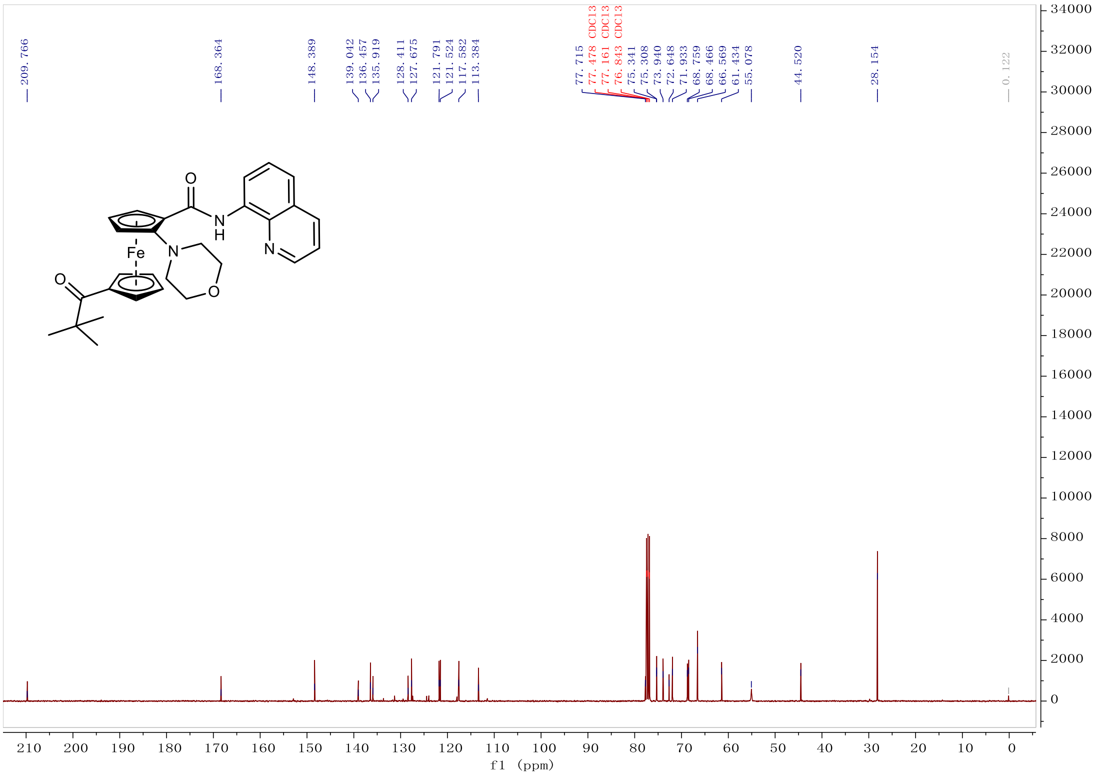

## 3k-$^{1}$H NMR

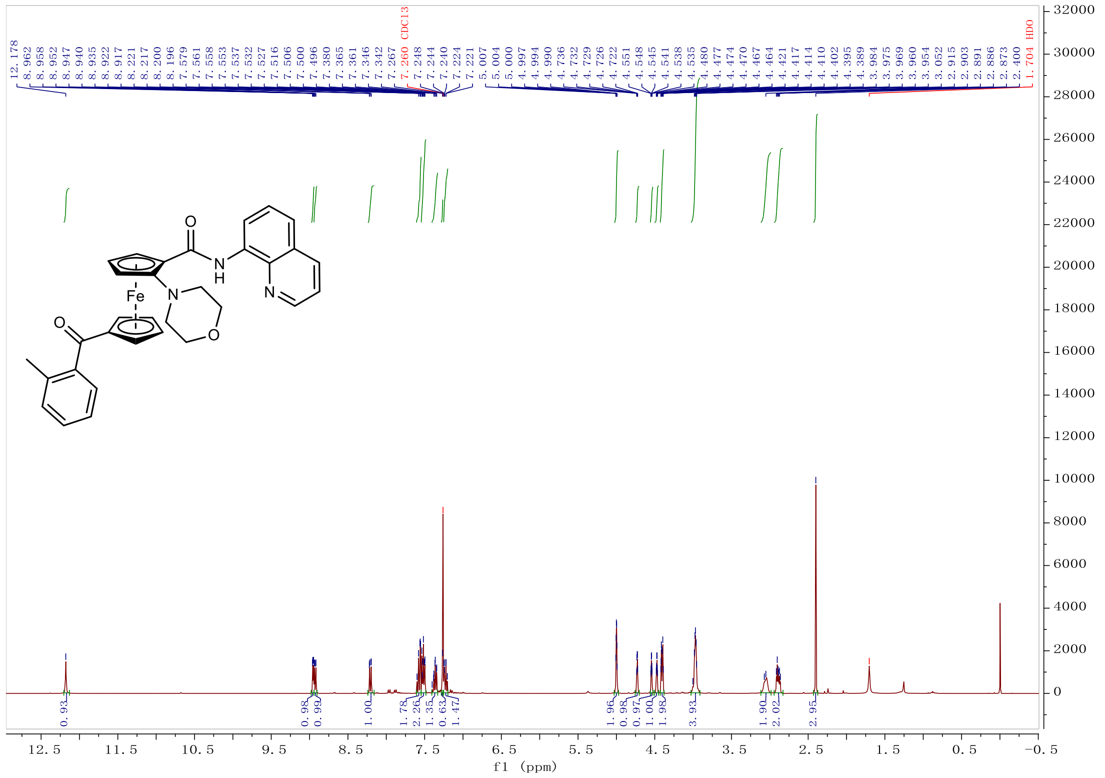

## 3k-$^{13}$C NMR

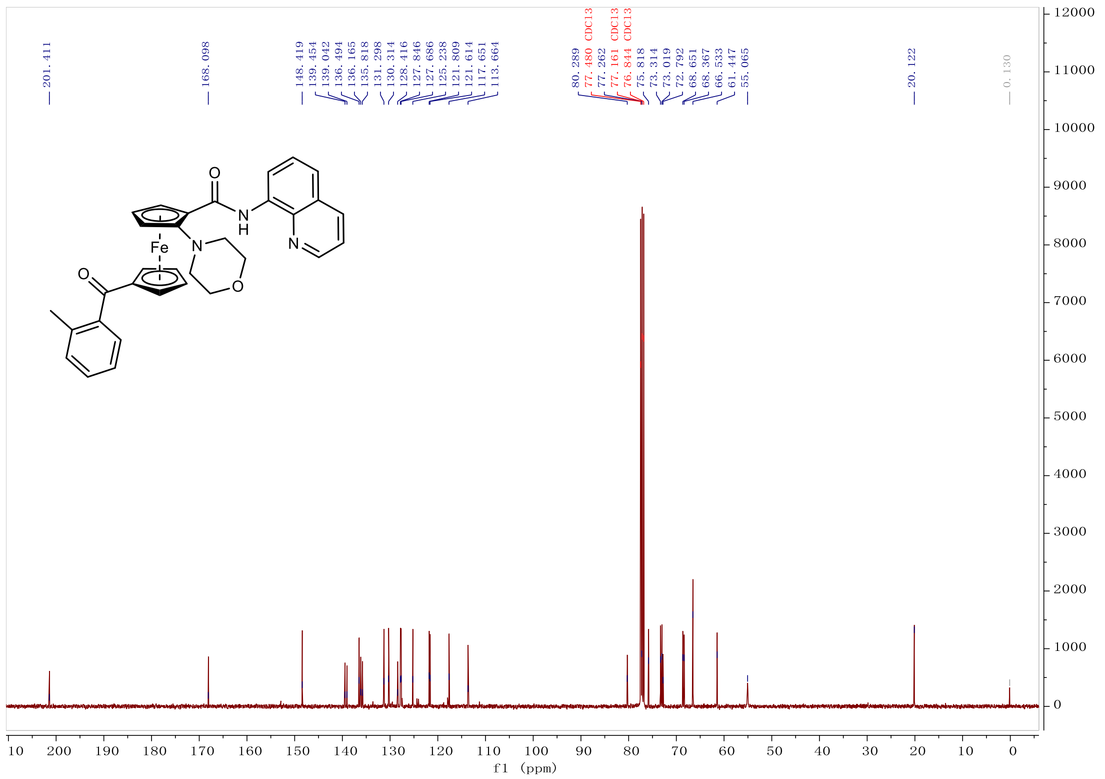

## 3l-$^{1}$H NMR

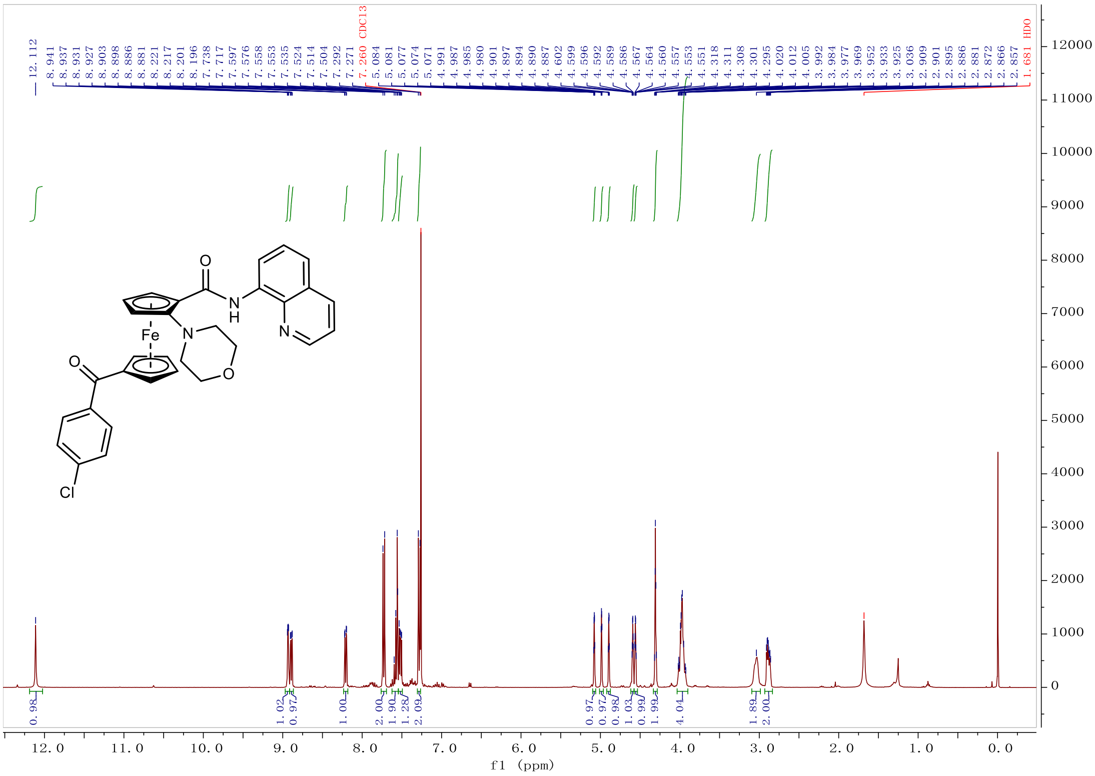

## 3l-$^{13}$C NMR

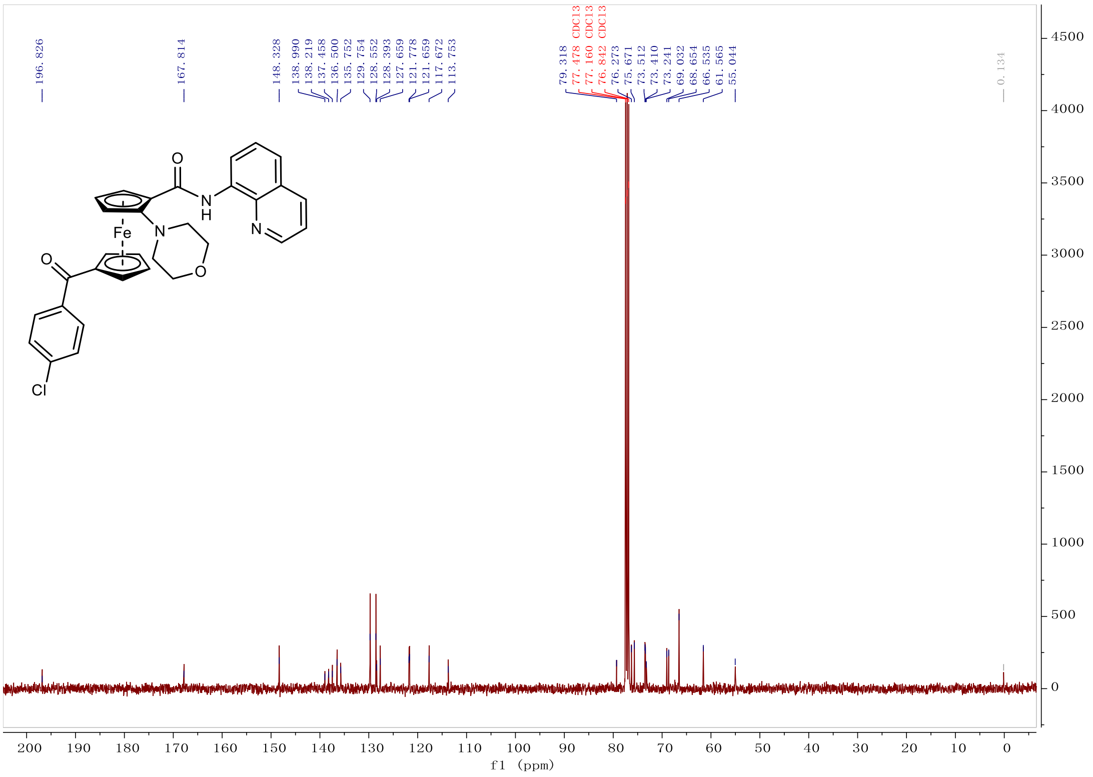

## 3m-$^1$H NMR

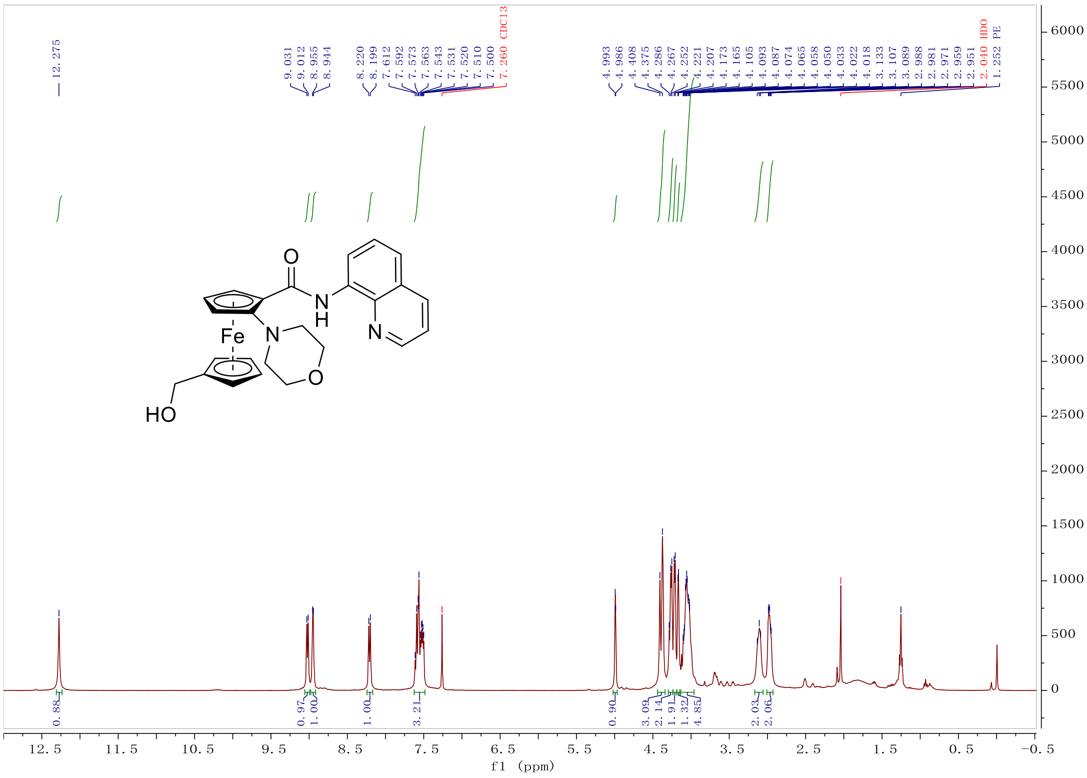


## 3m-$^{13}$C NMR

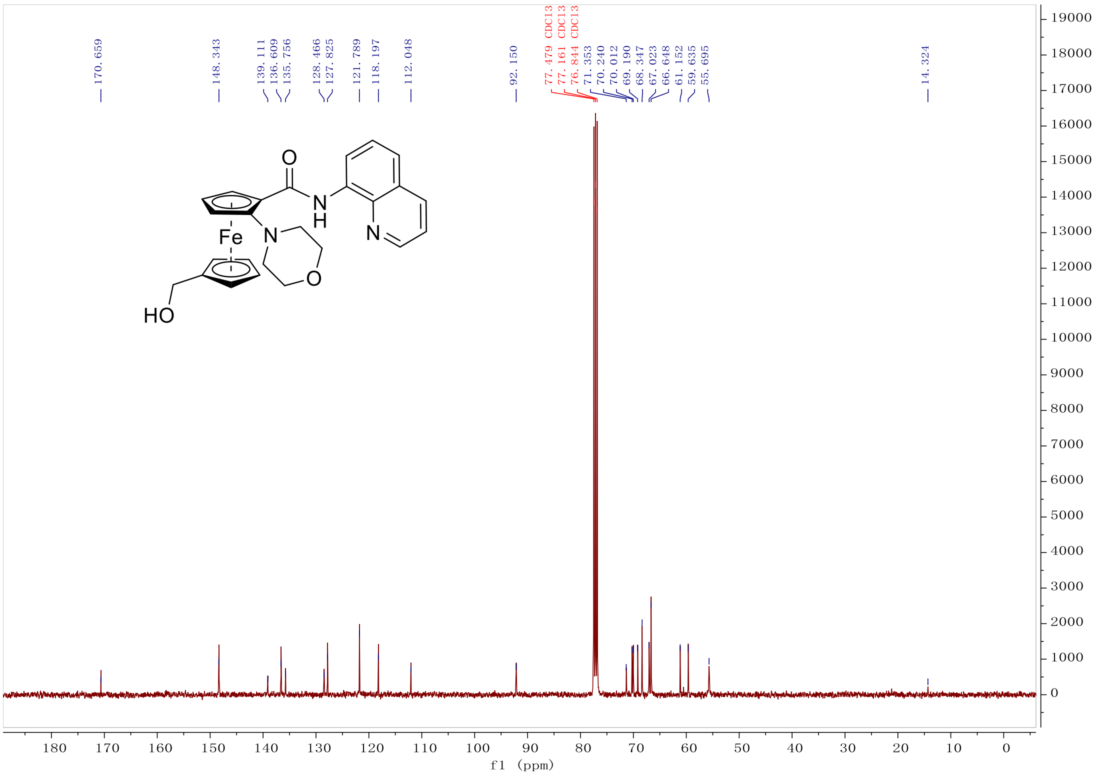

## 4a-$^{1}$H NMR

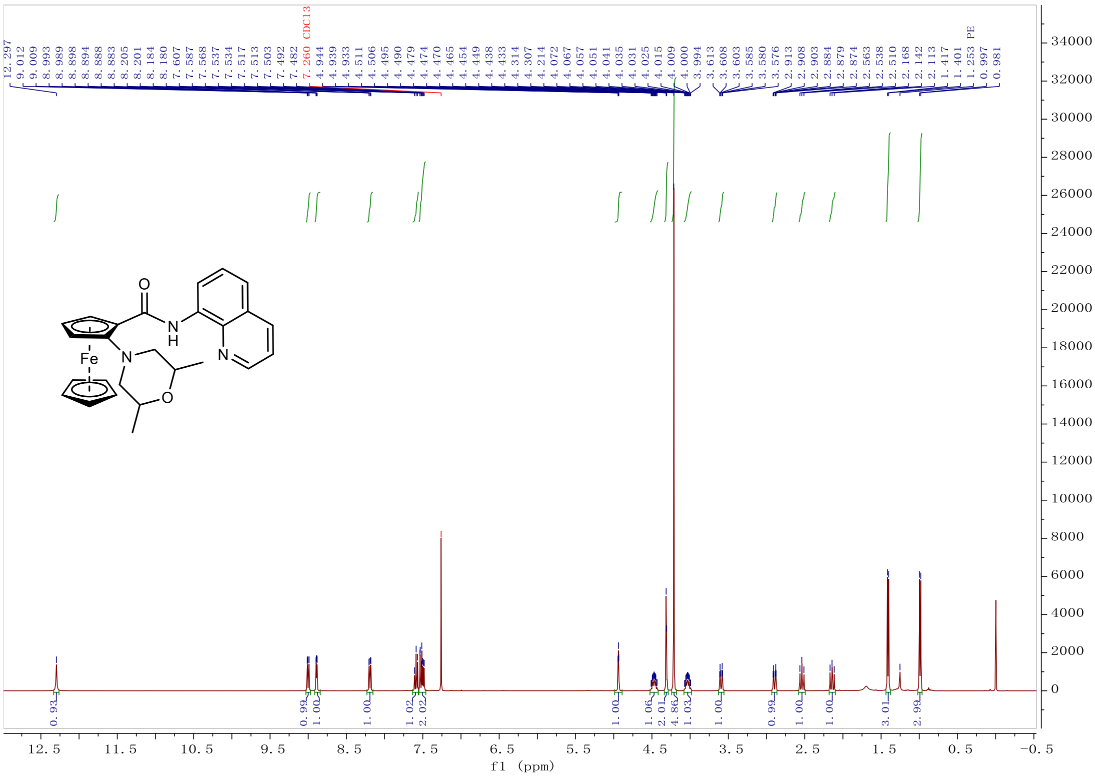

## 4a-$^{13}$C NMR

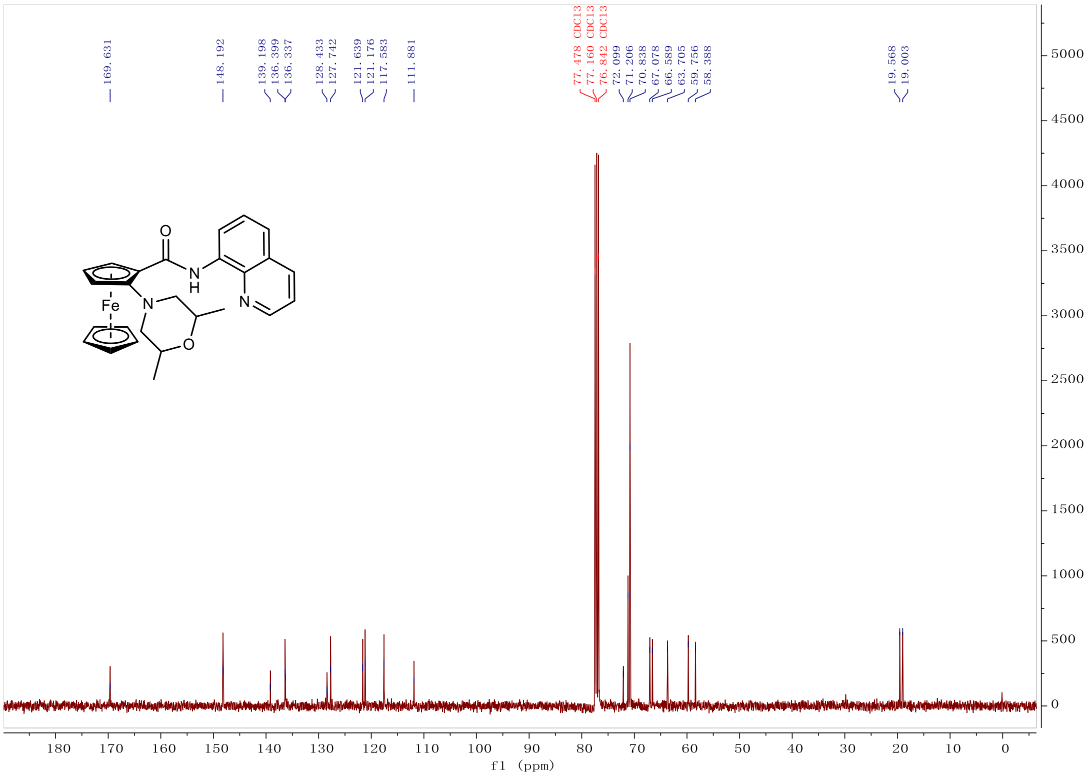

## 4b-1H NMR

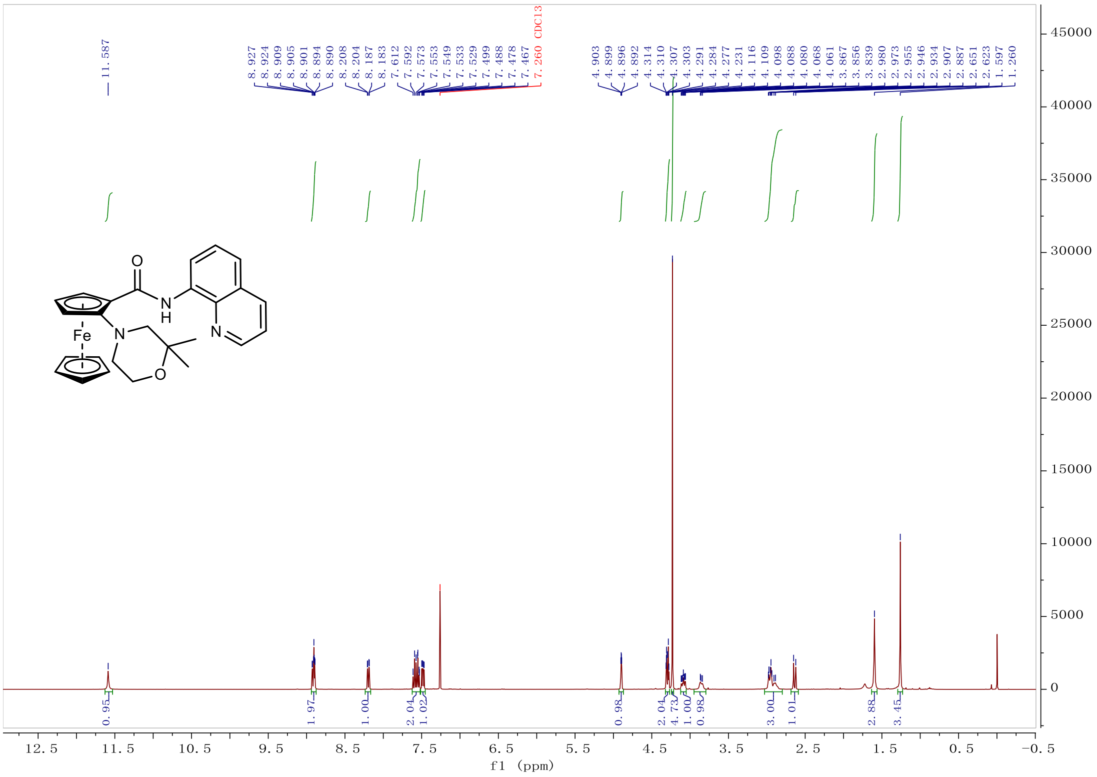

## 4b-13C NMR

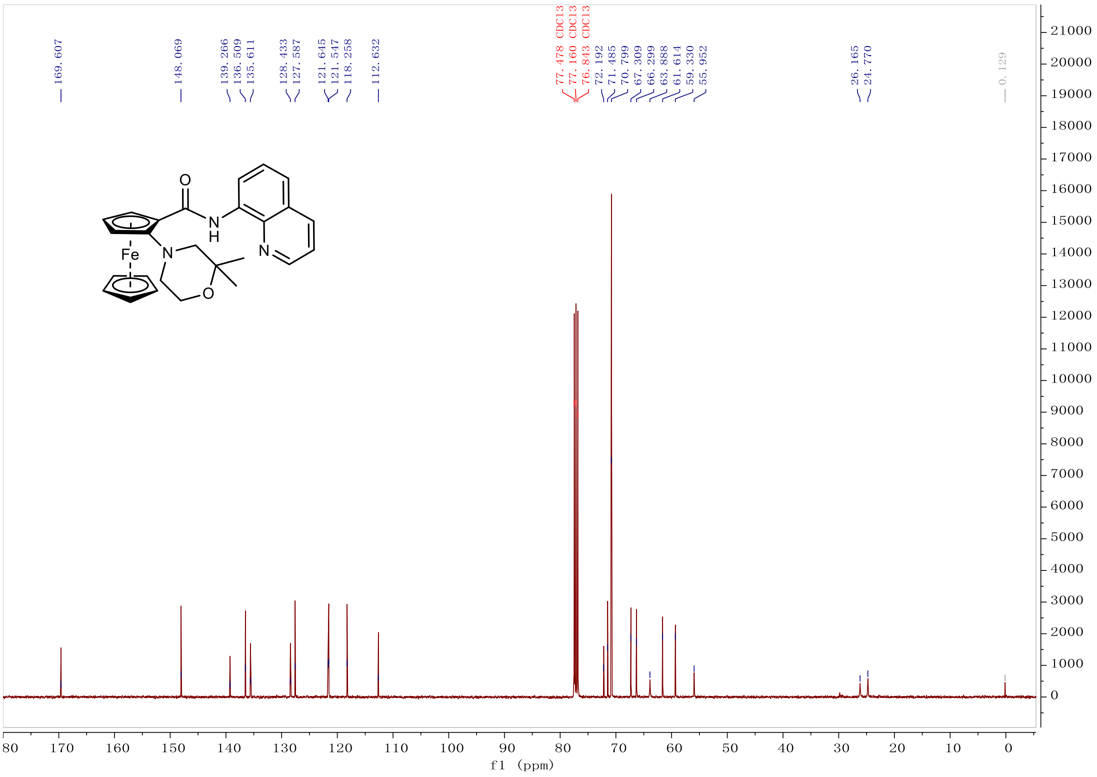

**4c-$^{1}$H NMR**

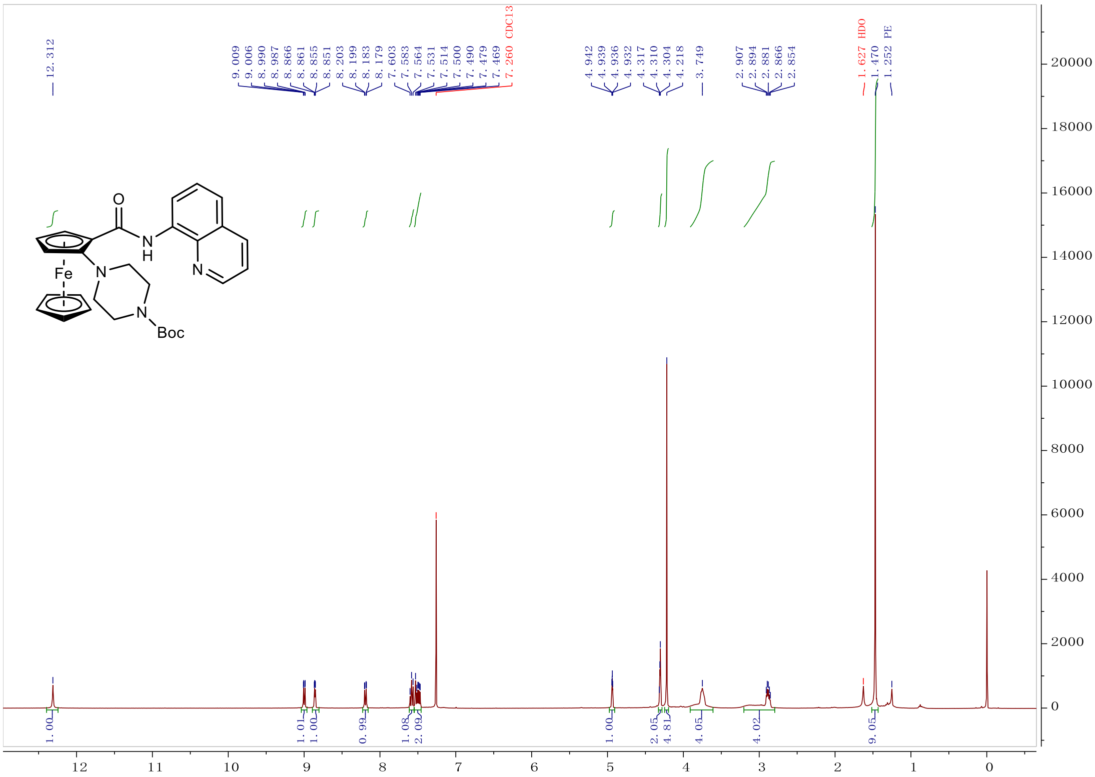

**4c-$^{13}$C NMR**

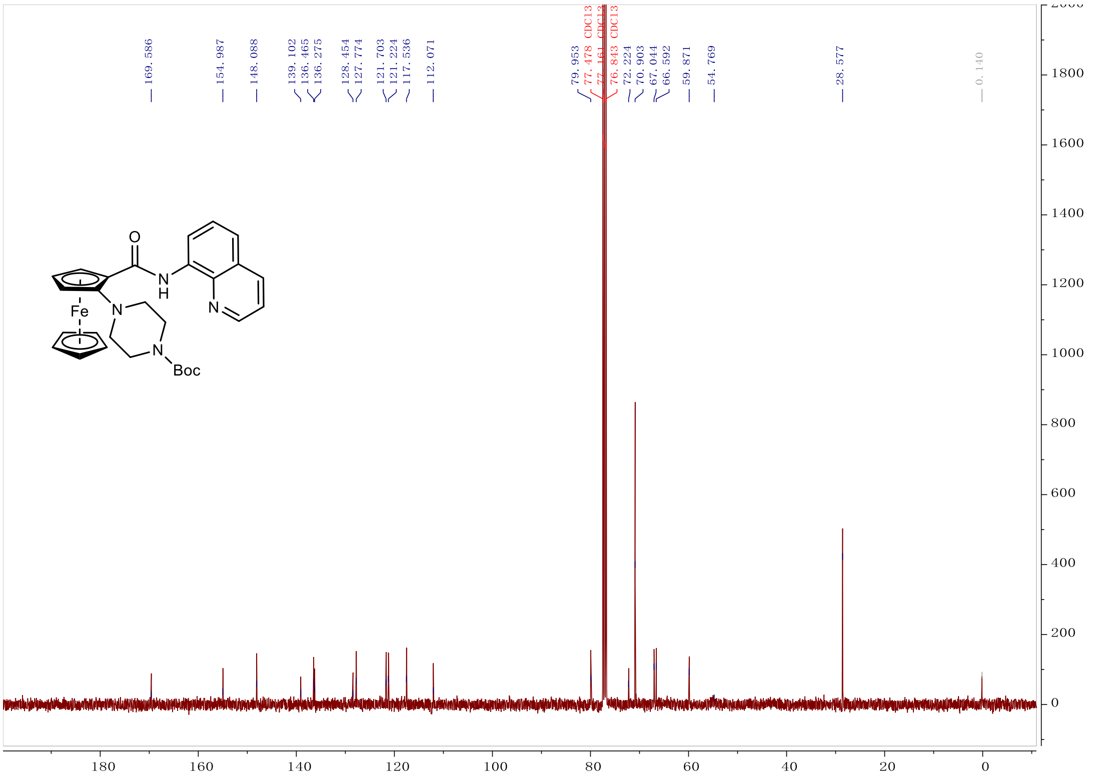

## 4d-$^{1}$H NMR

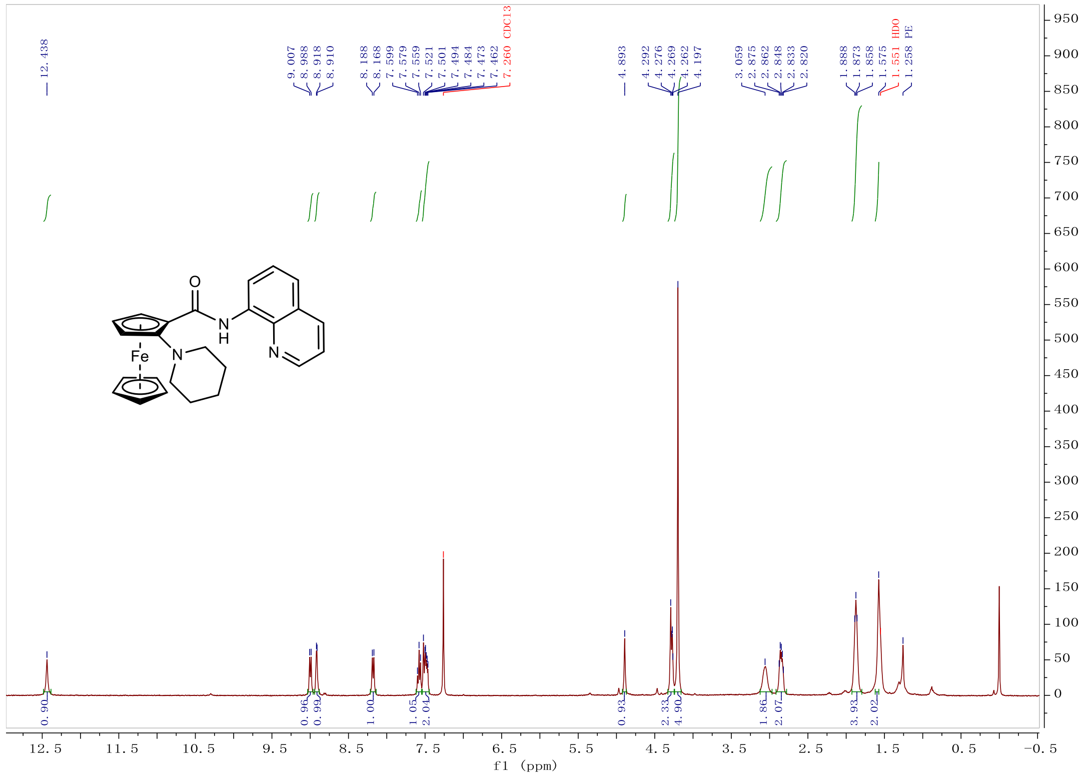


## 4d-$^{13}$C NMR

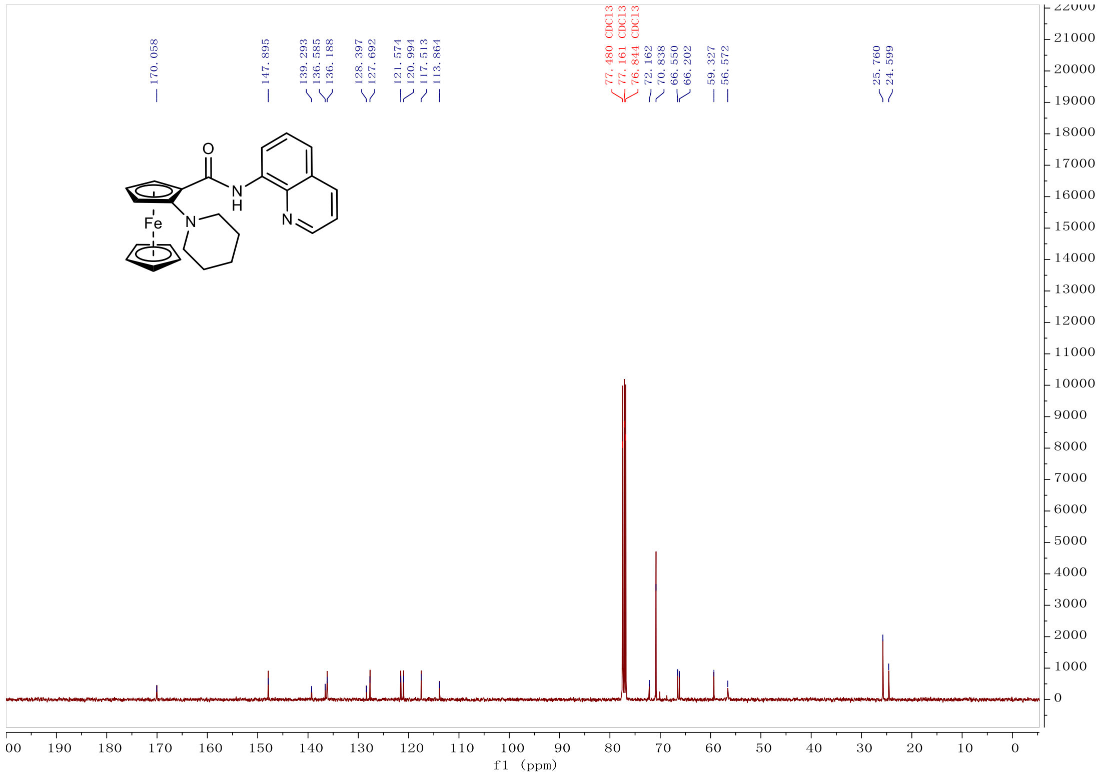

## 4e-$^{1}$H NMR

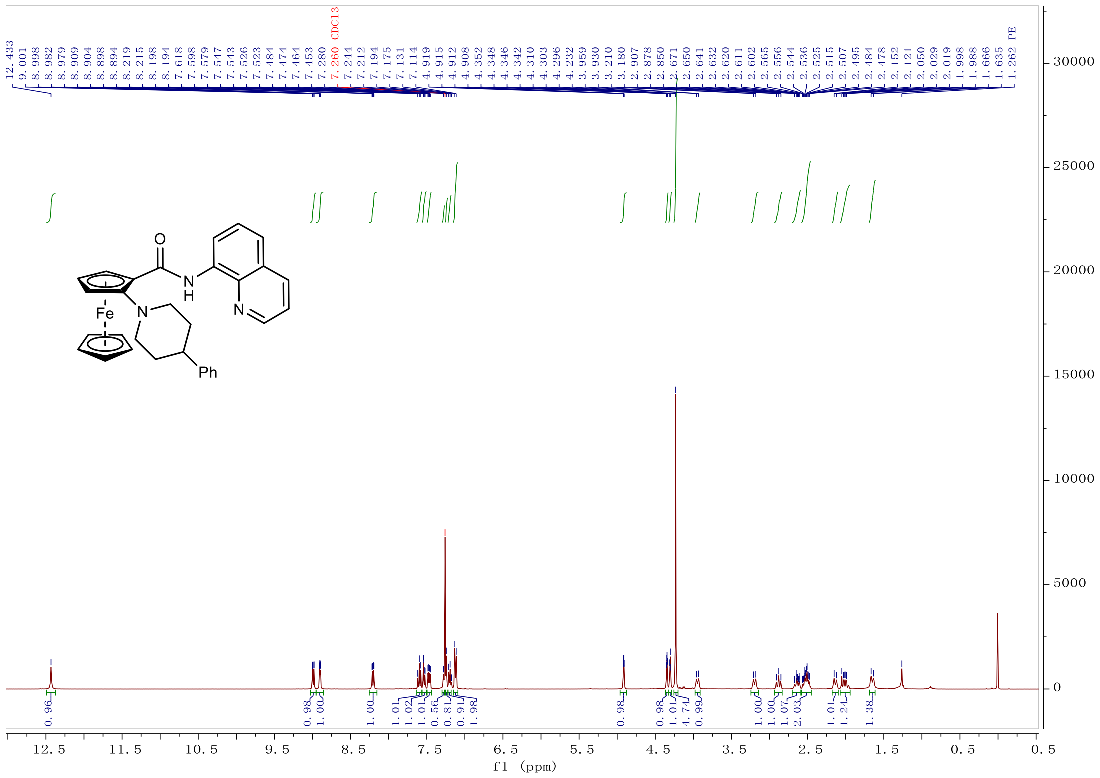

## 4e-$^{13}$C NMR

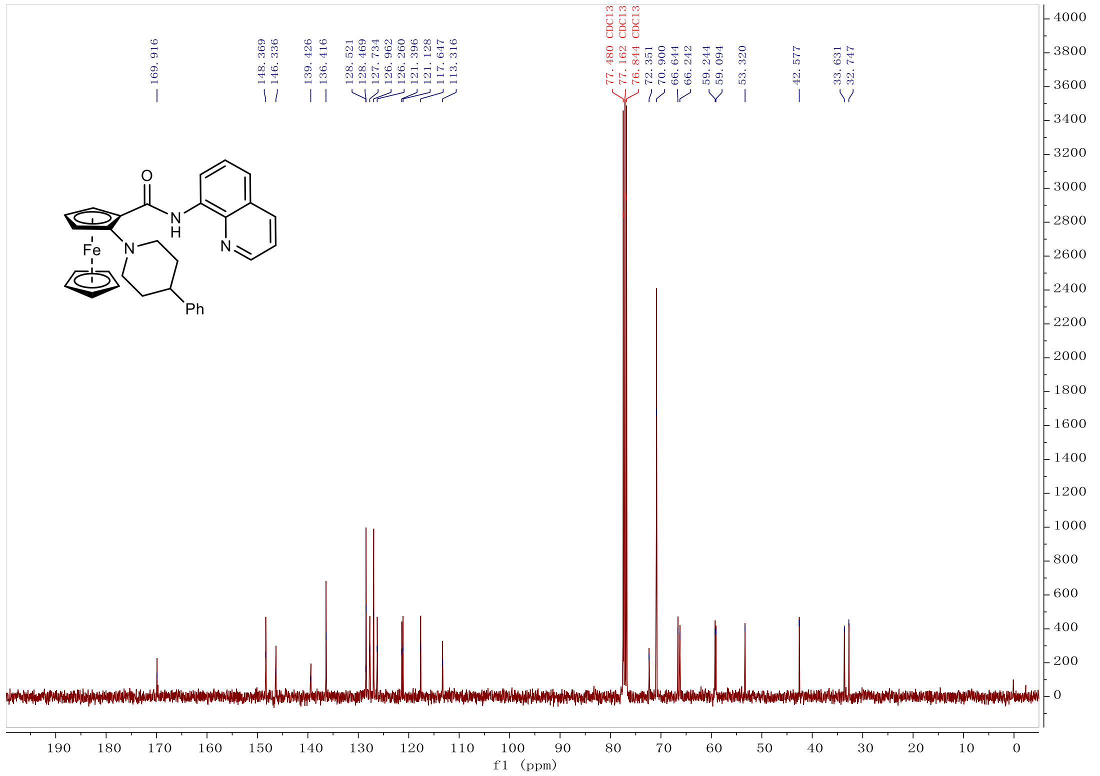

## 4f-¹H NMR

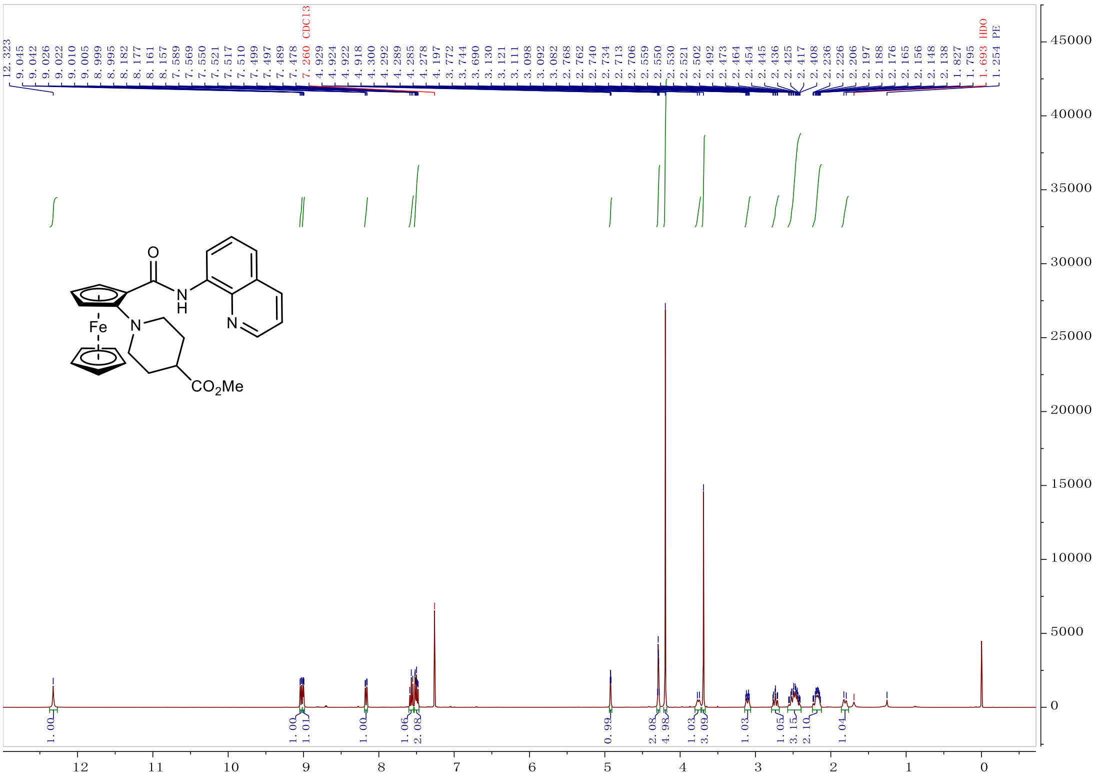

## 4f-¹³C NMR

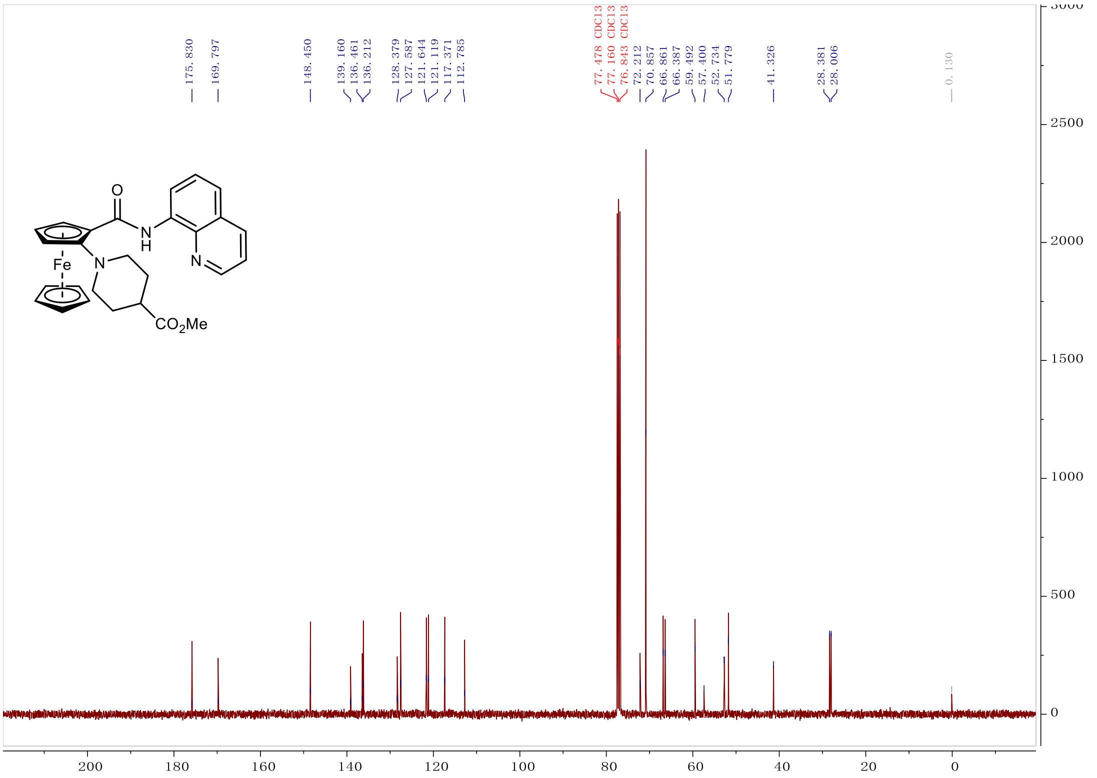

## 4g-$^{1}$H NMR

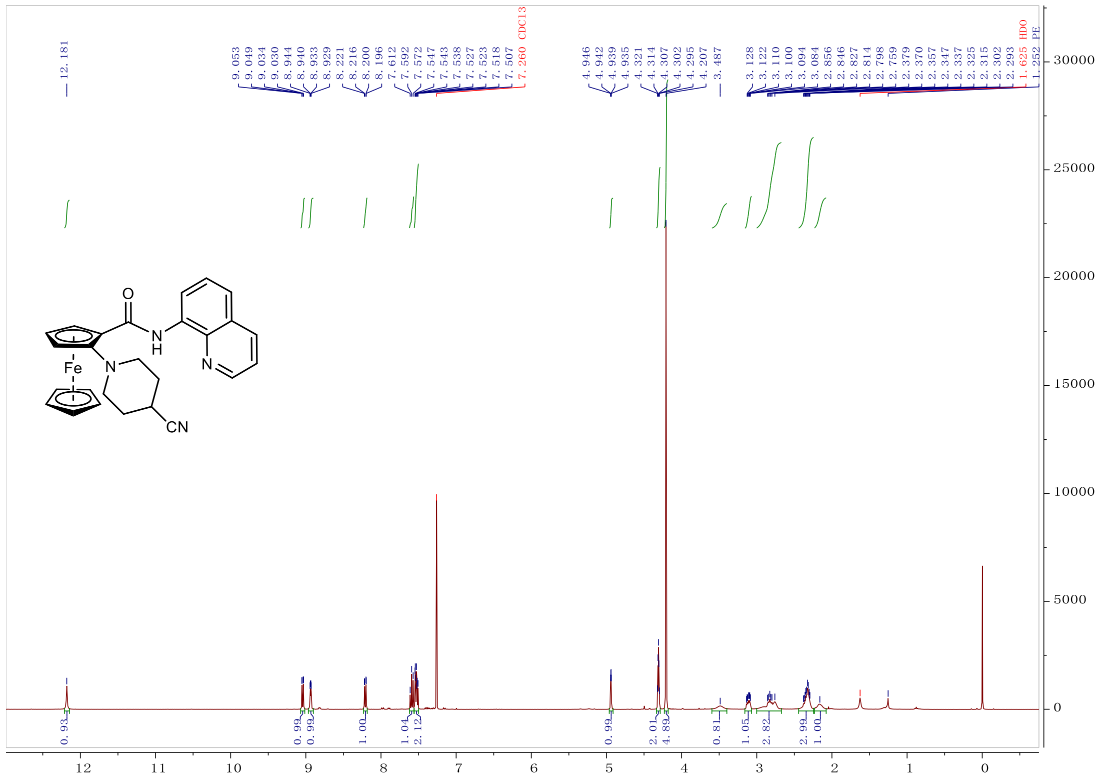

## 4g-$^{13}$C NMR

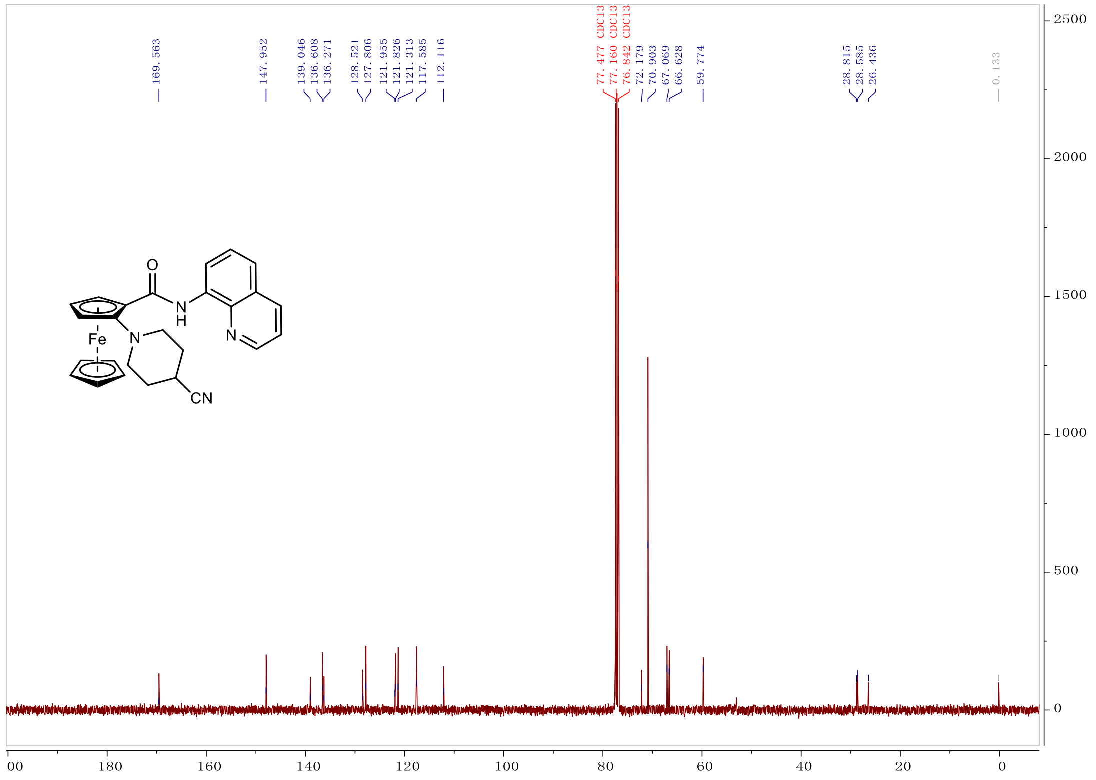

## 4h-$^{1}$H NMR

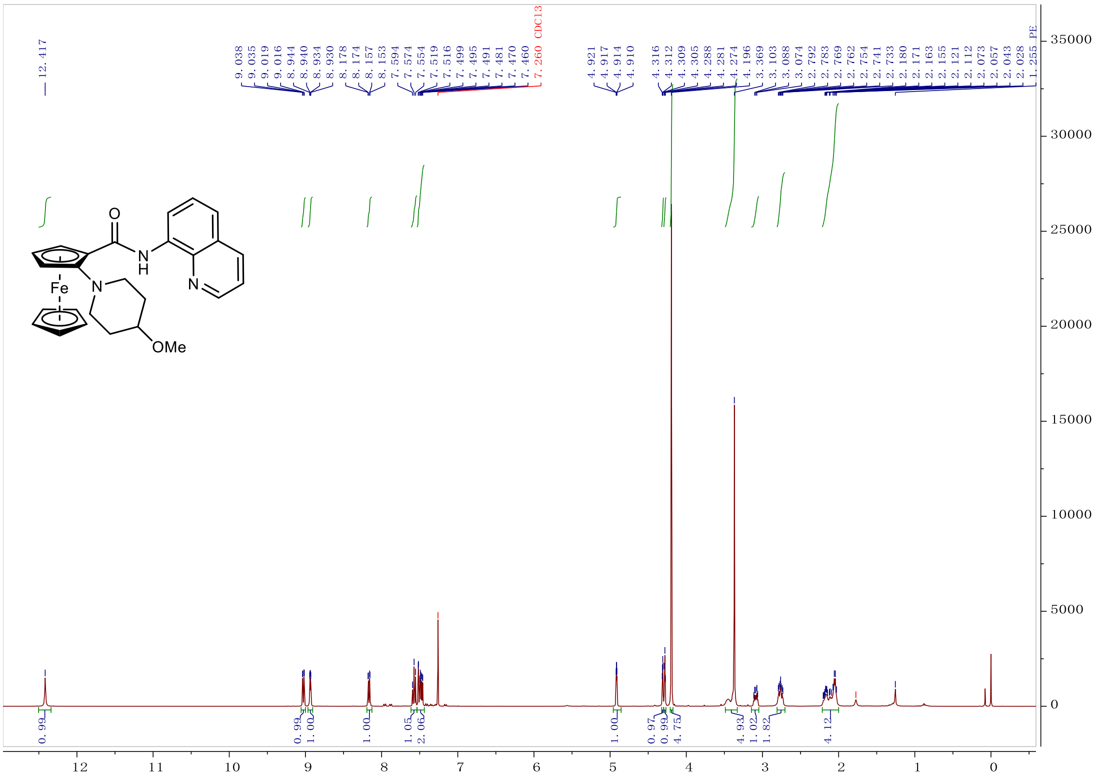

## 4h-$^{13}$C NMR

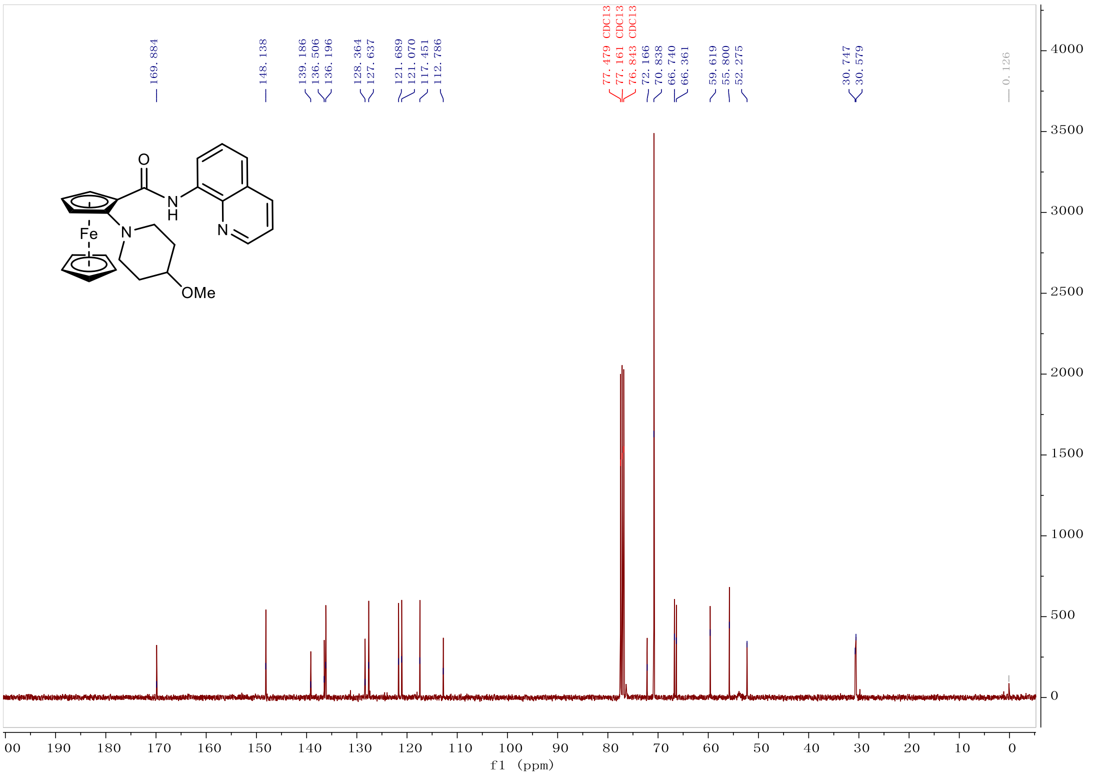

## 4i-$^{1}$H NMR

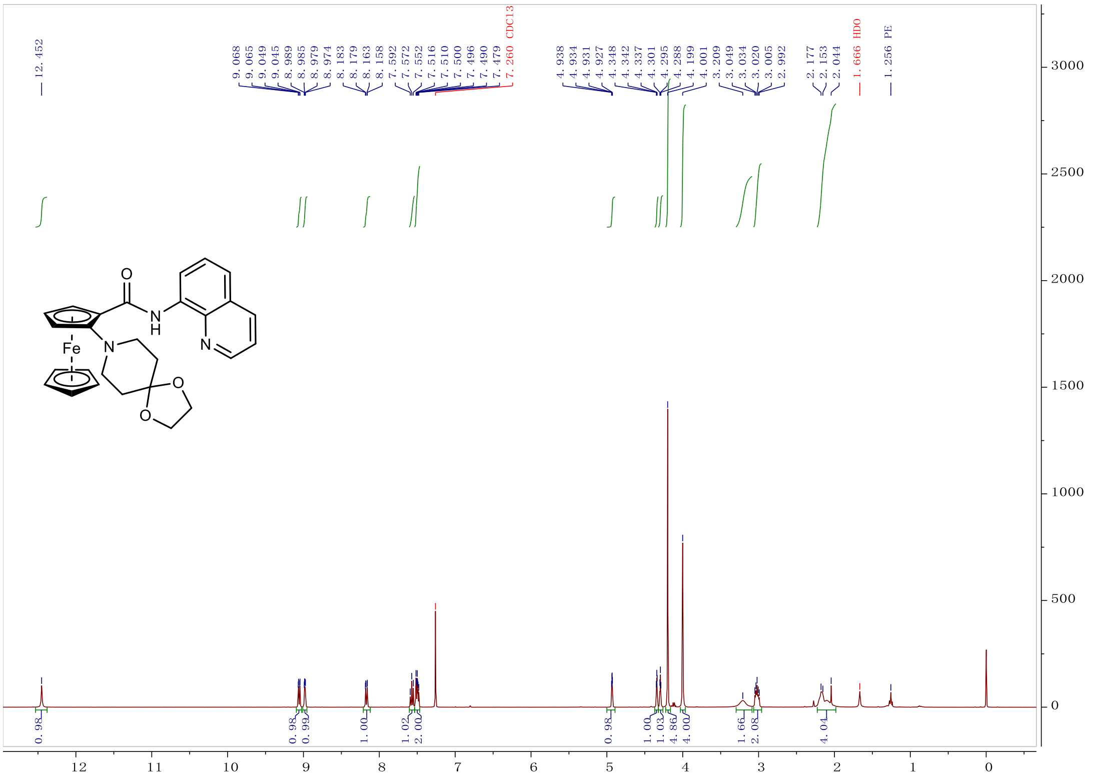


## 4i-$^{13}$C NMR

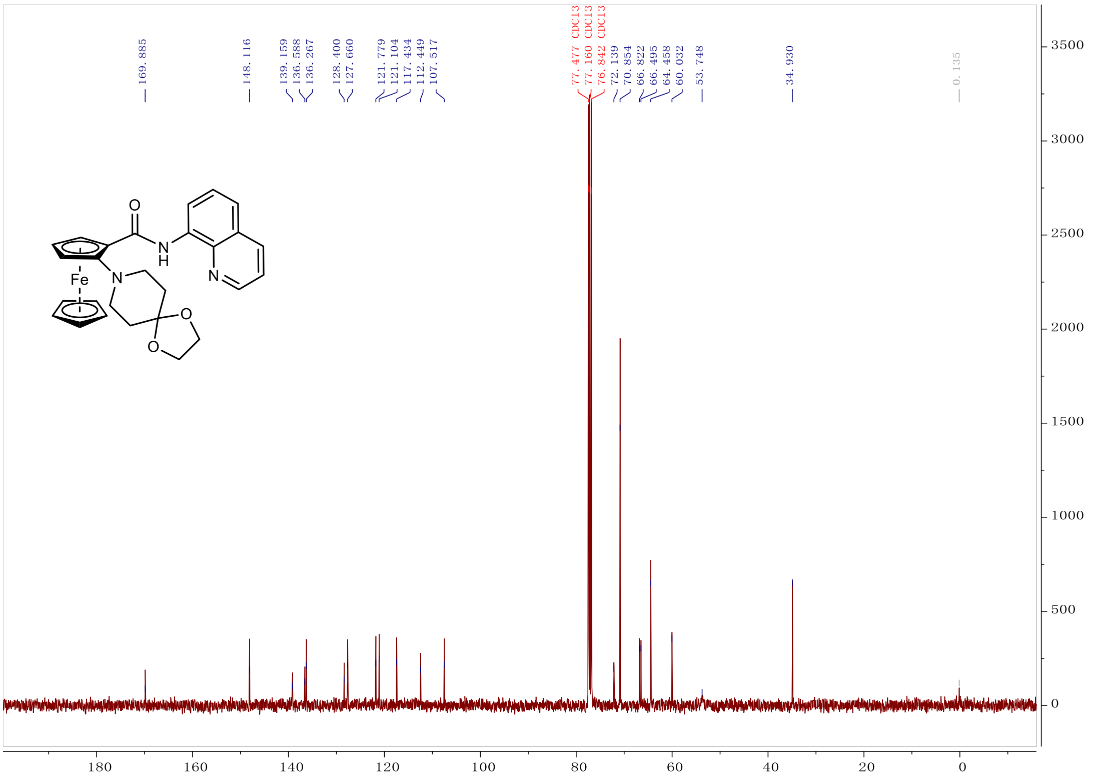

## 4j-$^{1}$H NMR

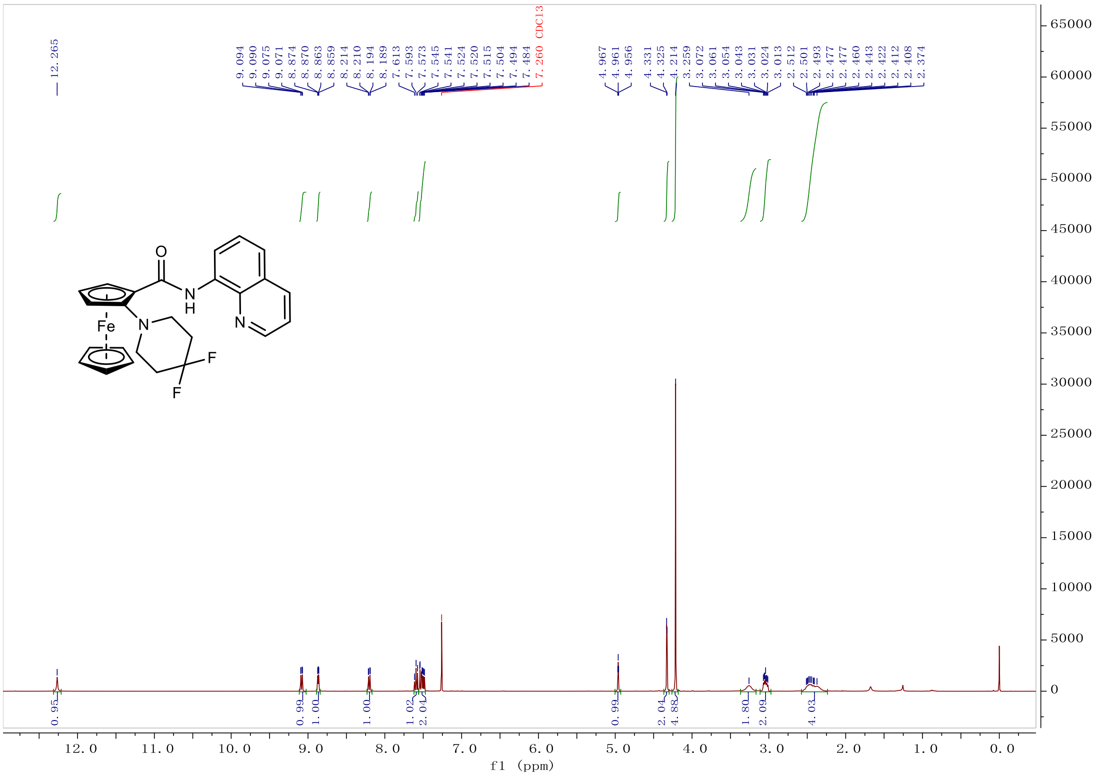

## 4j-$^{13}$C NMR

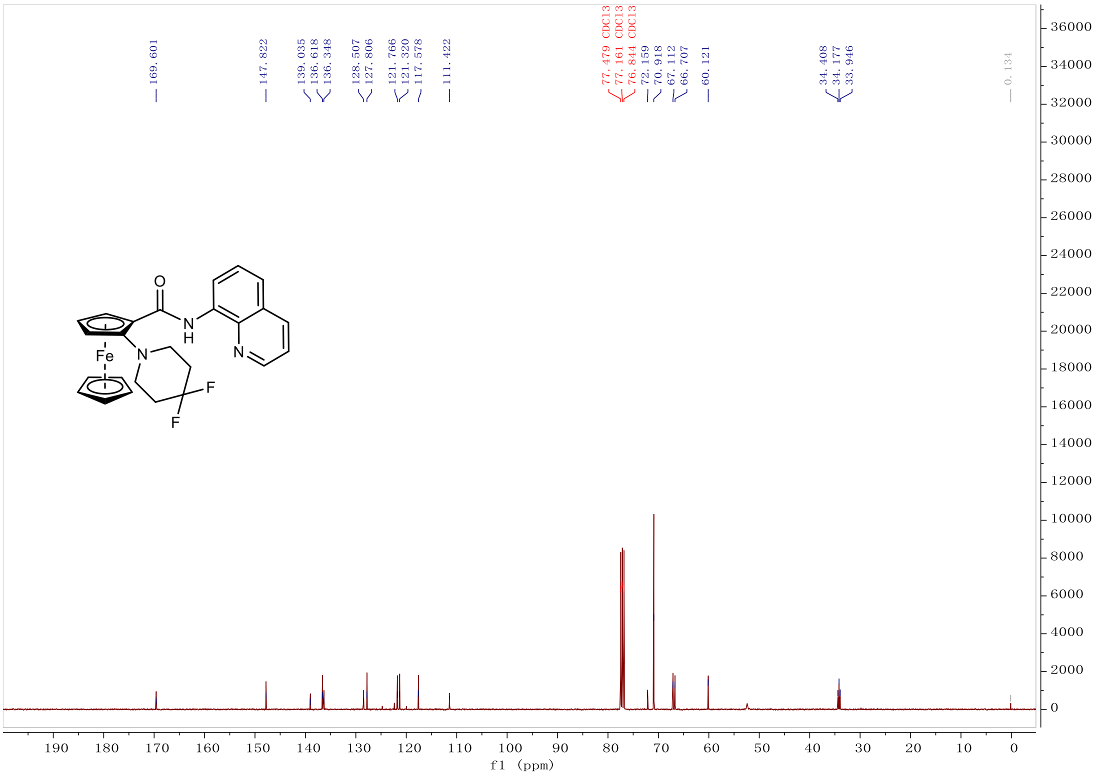

## 4k-$^{1}$H NMR

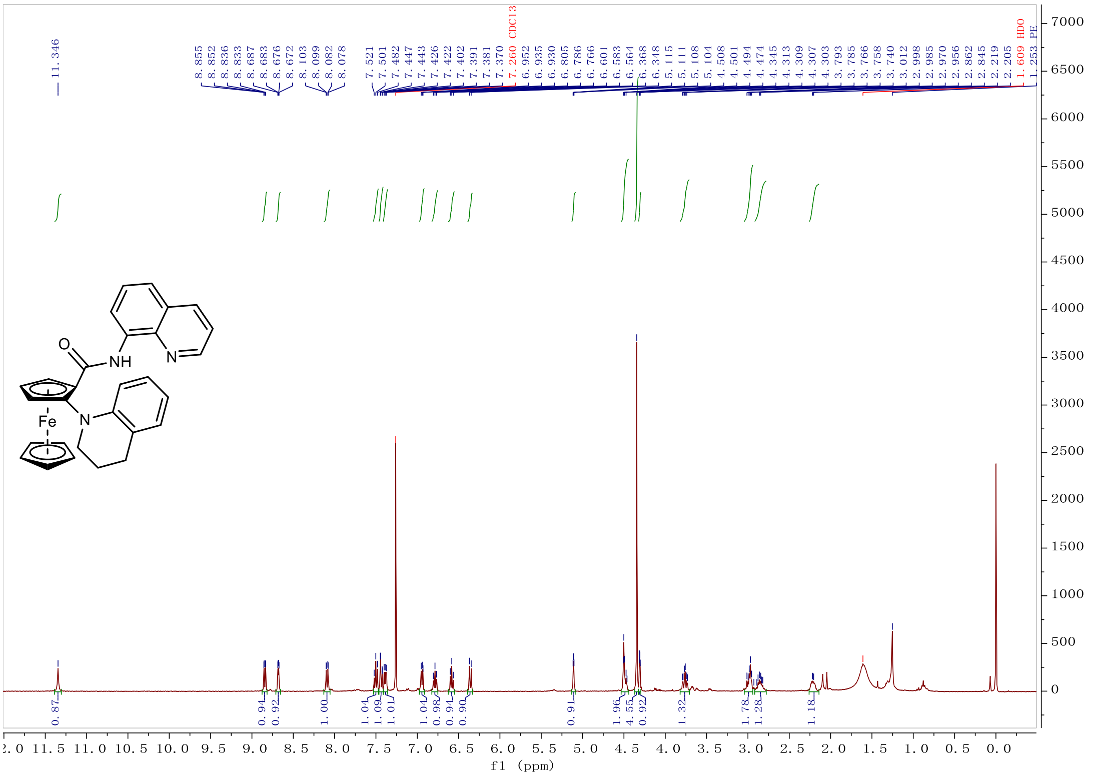

## 4k-$^{13}$C NMR

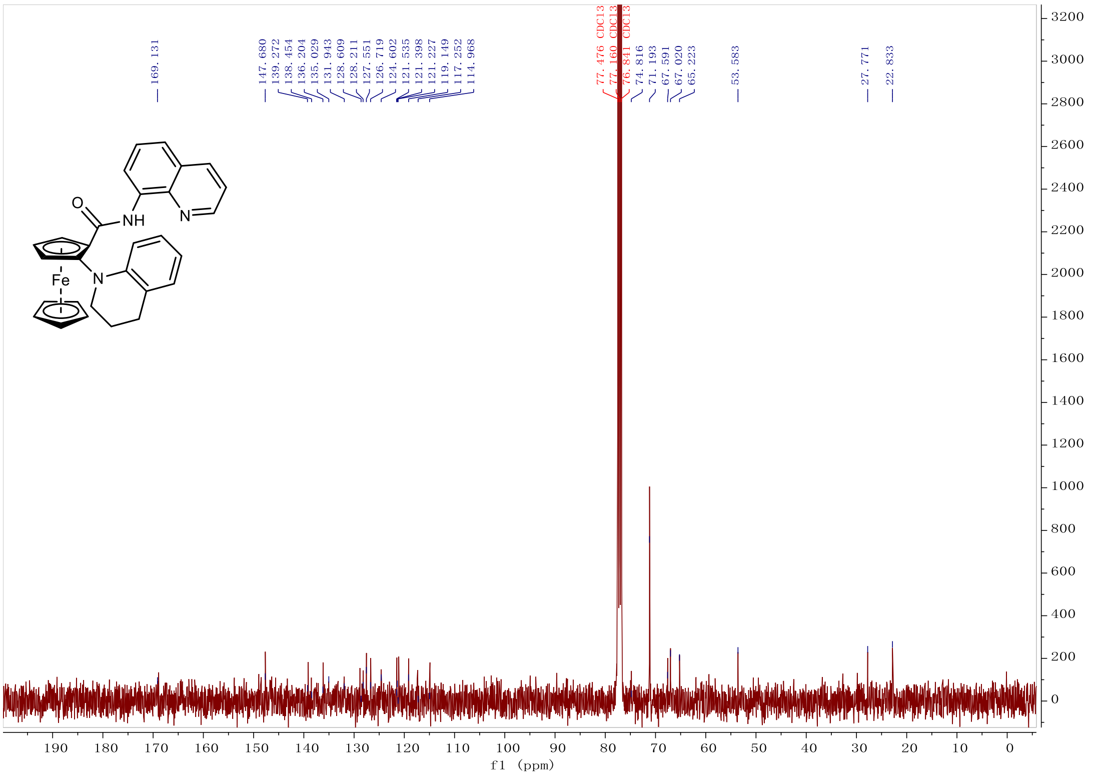

**4l-1H NMR**

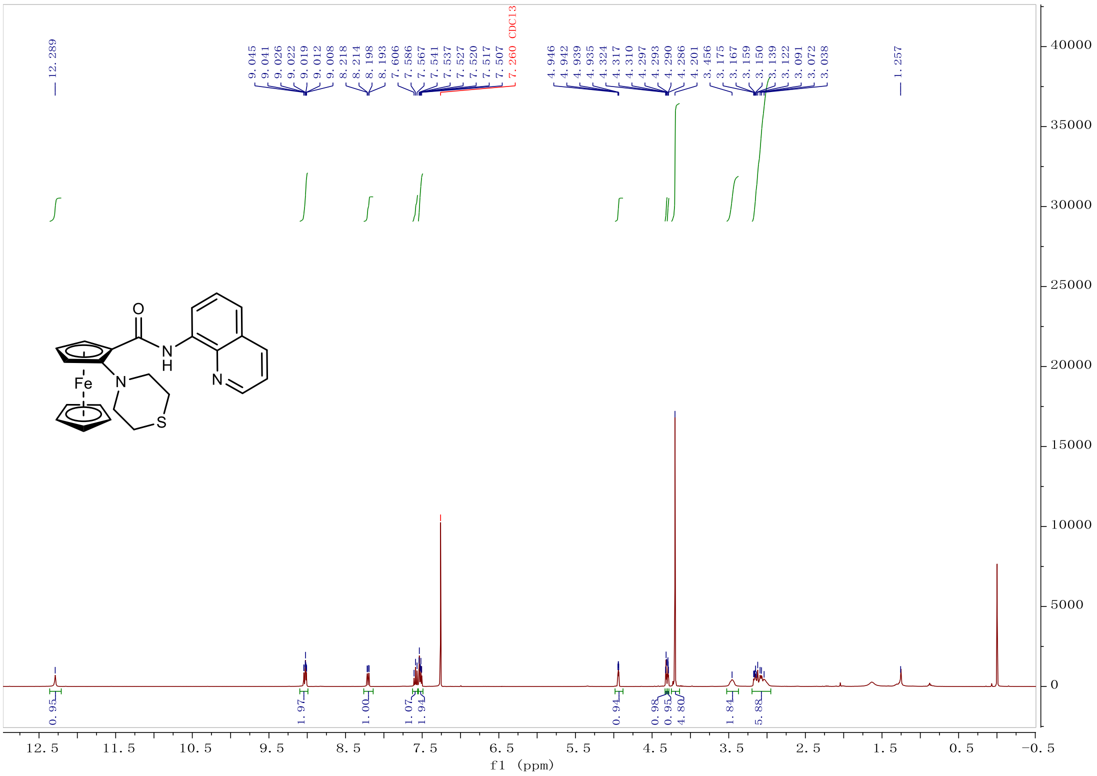

**4l-13C NMR**

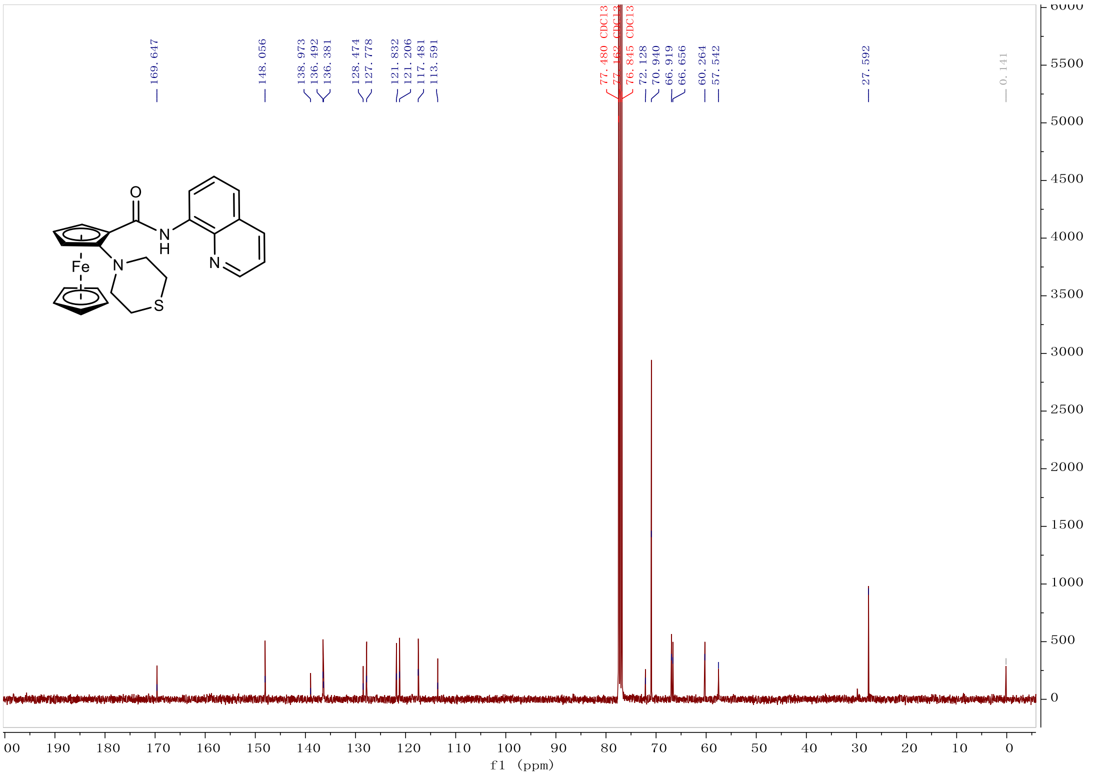

## 4m-$^{1}$H NMR

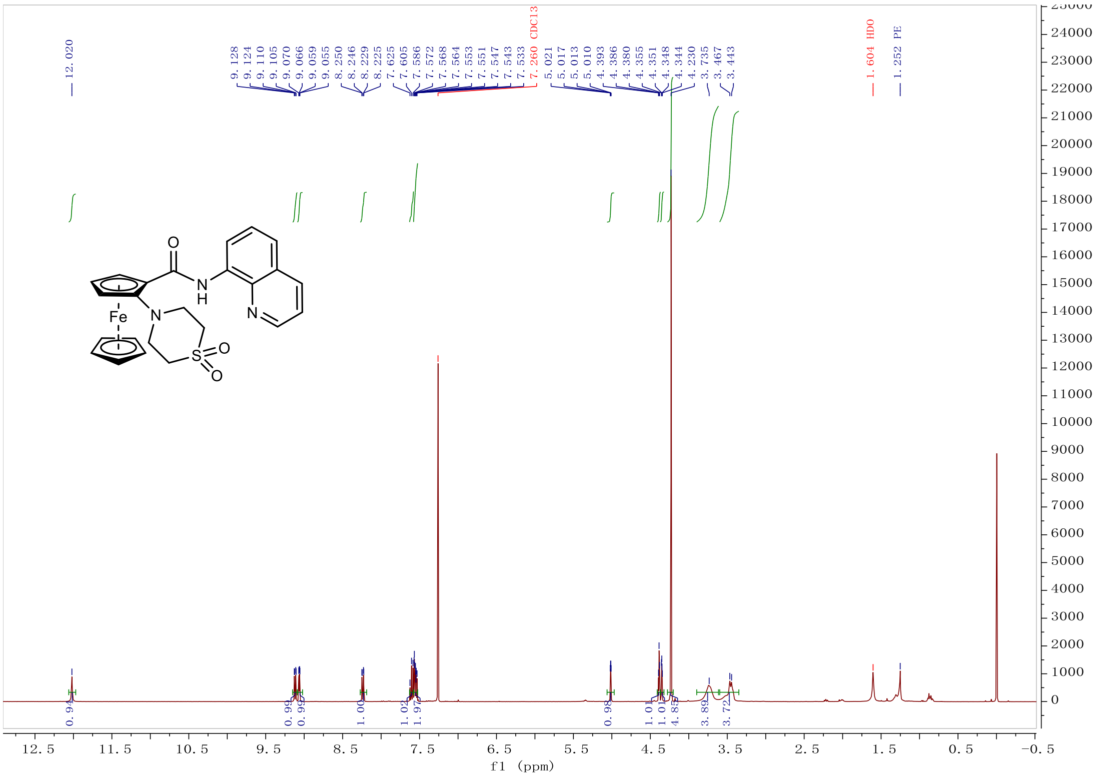

## 4m-$^{13}$C NMR

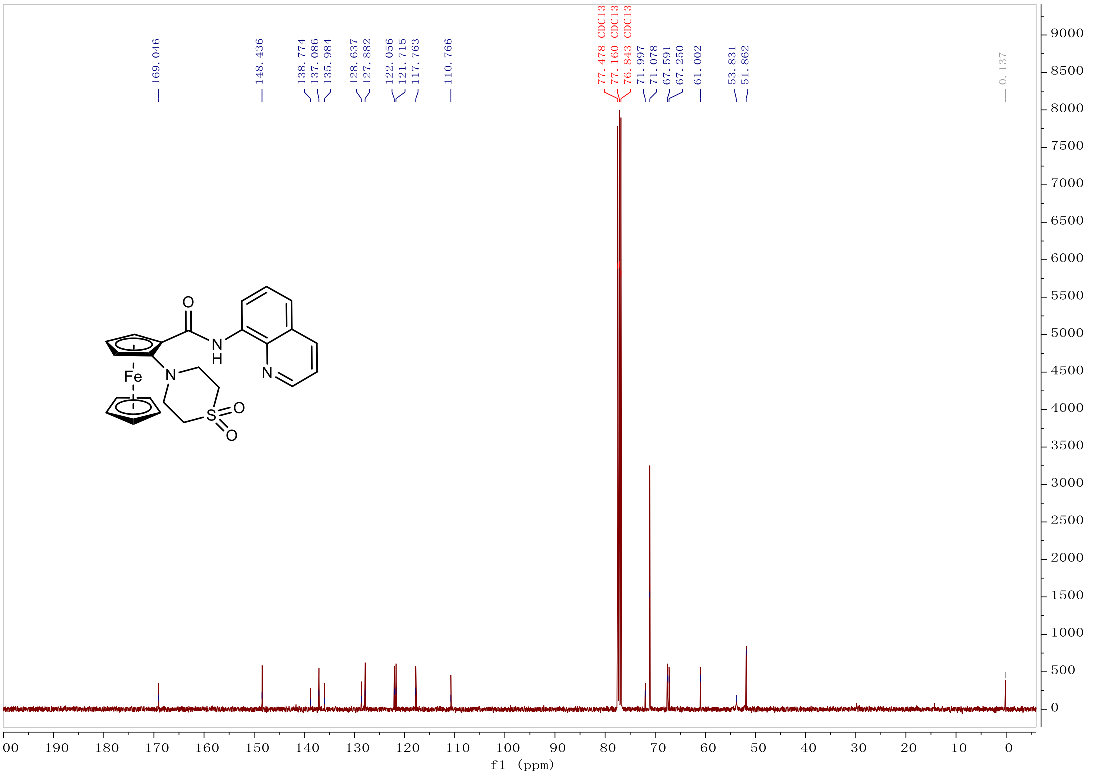

## 4n-$^1$H NMR

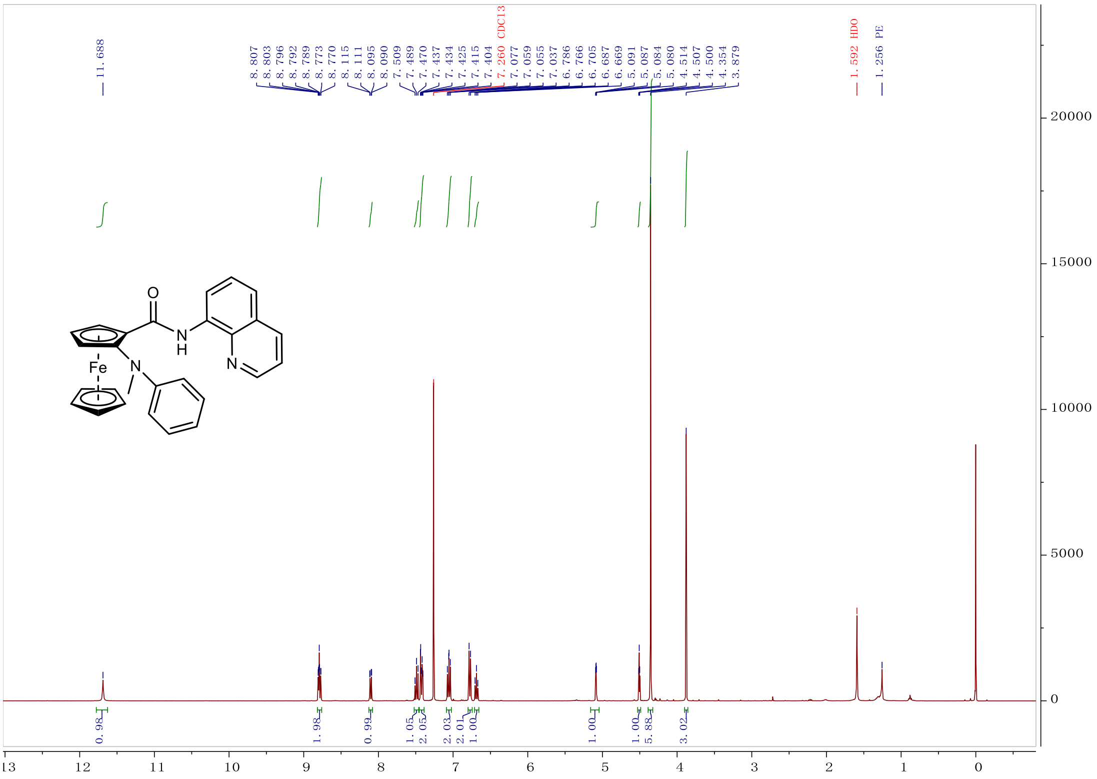


## 4n-$^{13}$C NMR

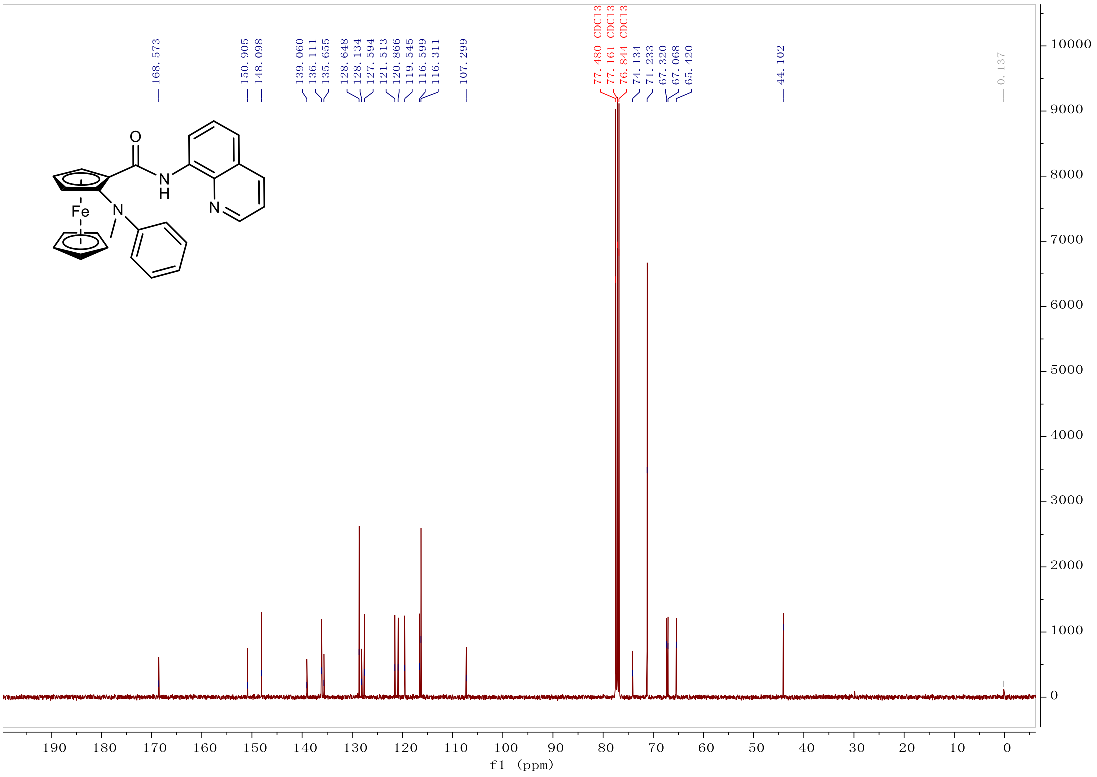

## 4o-$^1$H NMR

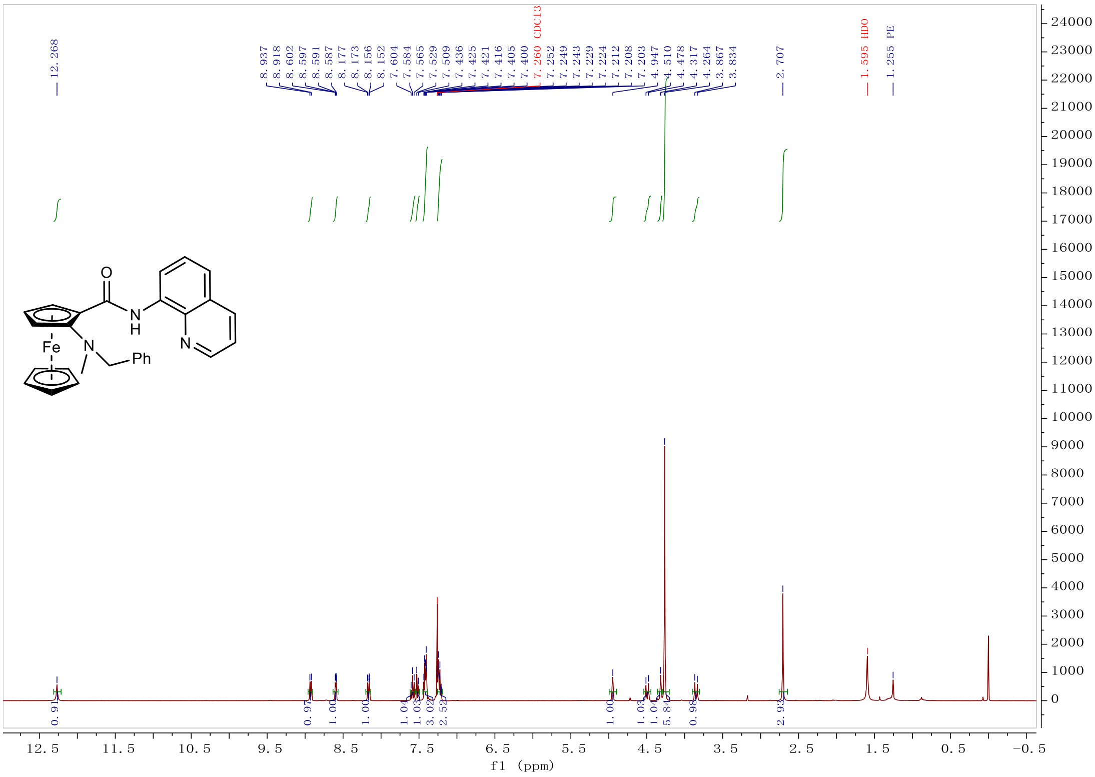

## 4o-$^{13}$C NMR

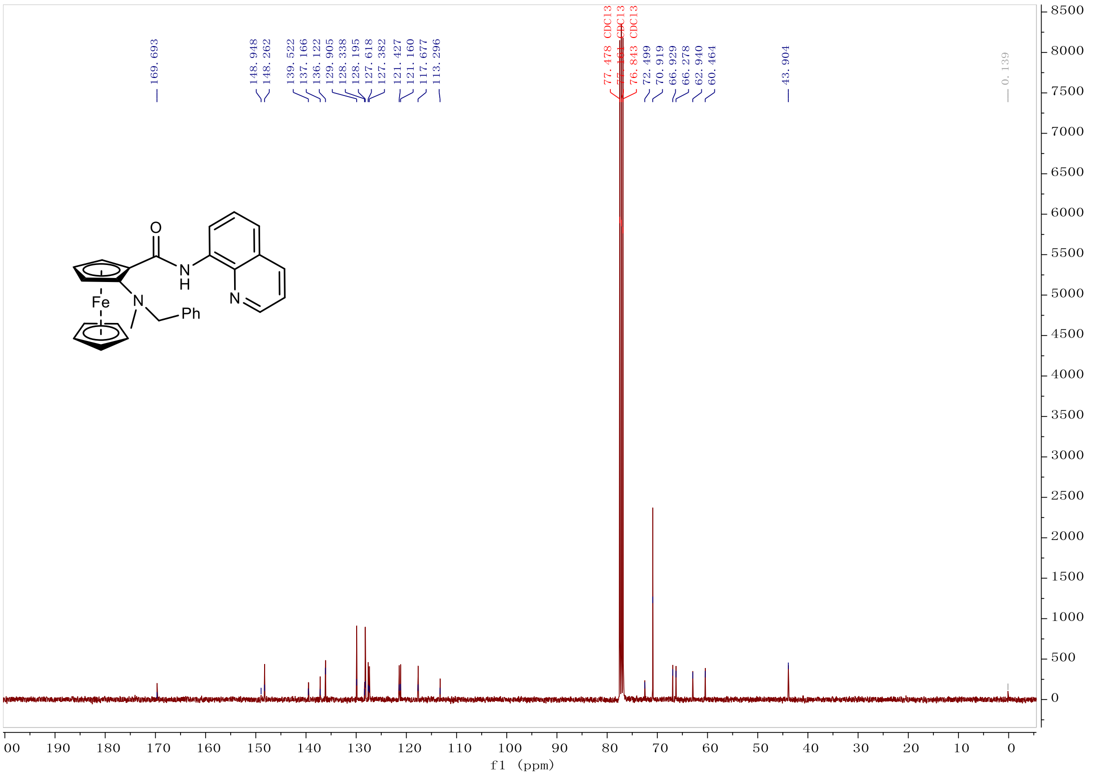

## 4p-$^{1}$H NMR

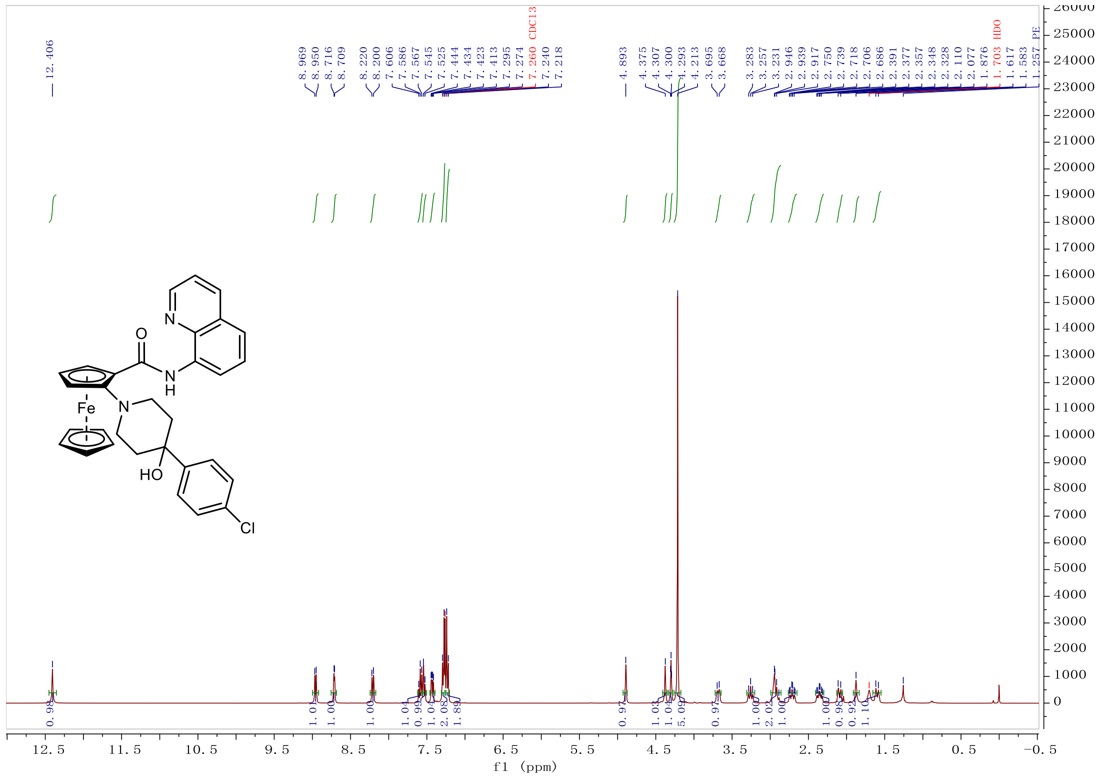

## 4p-$^{13}$C NMR

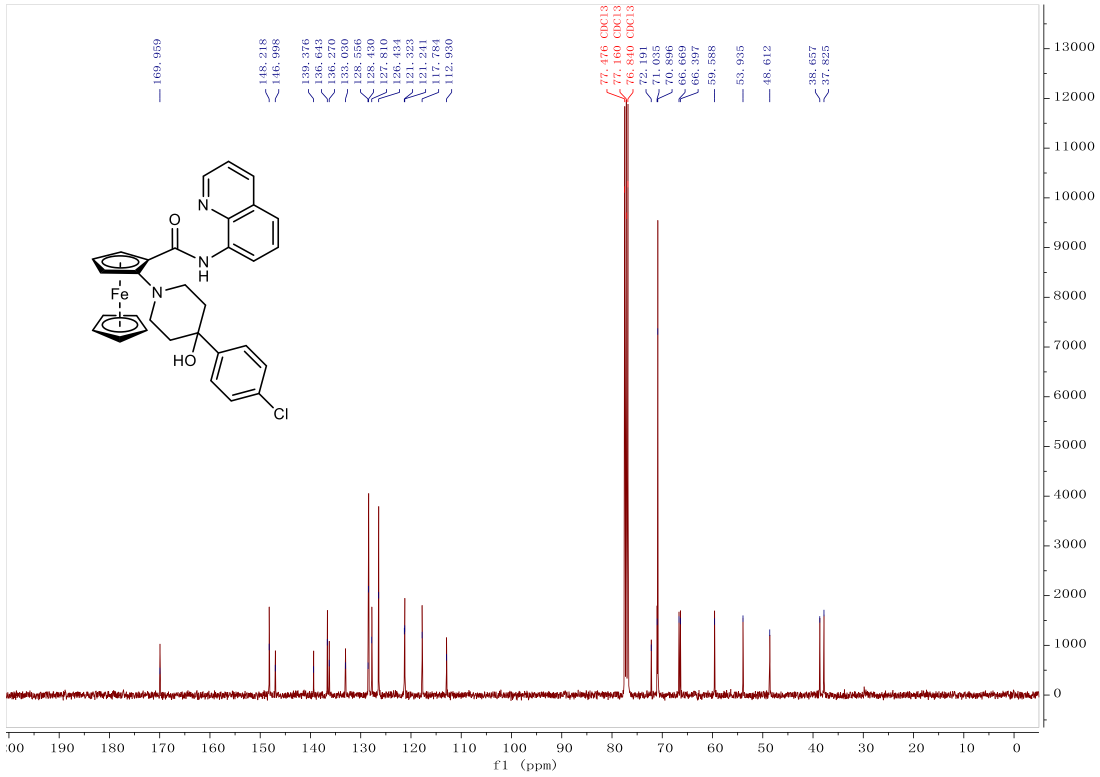

## 4q-¹H NMR

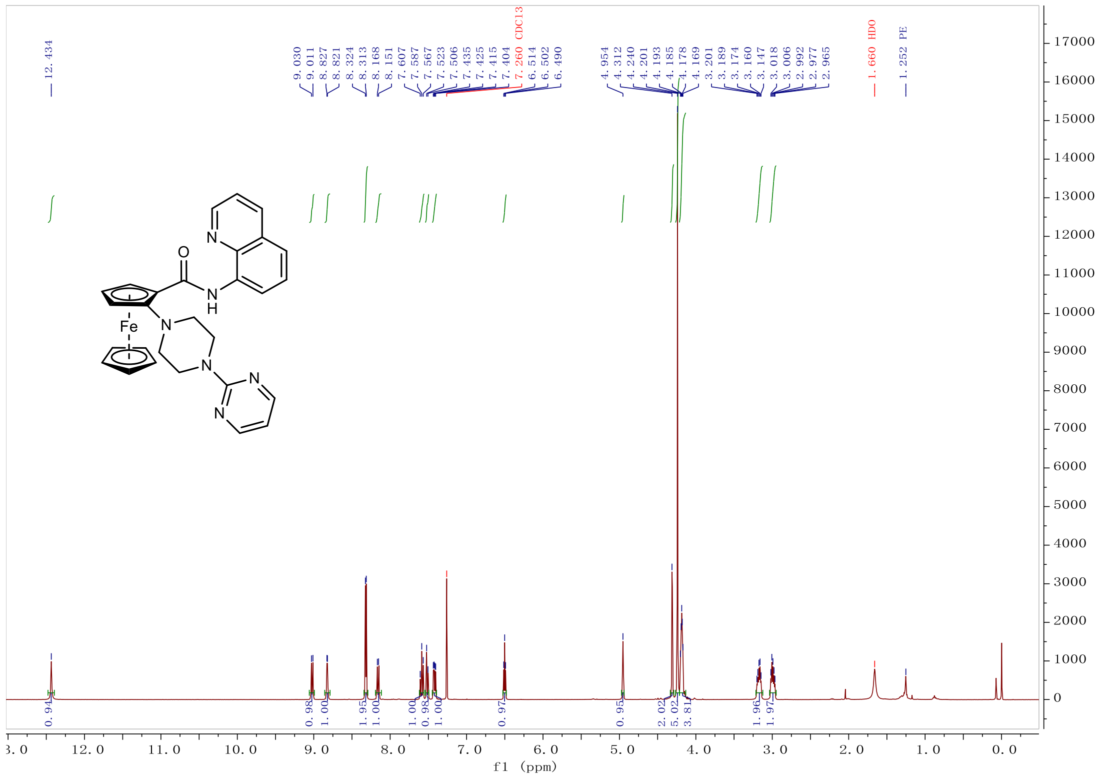

## 4q-¹³C NMR

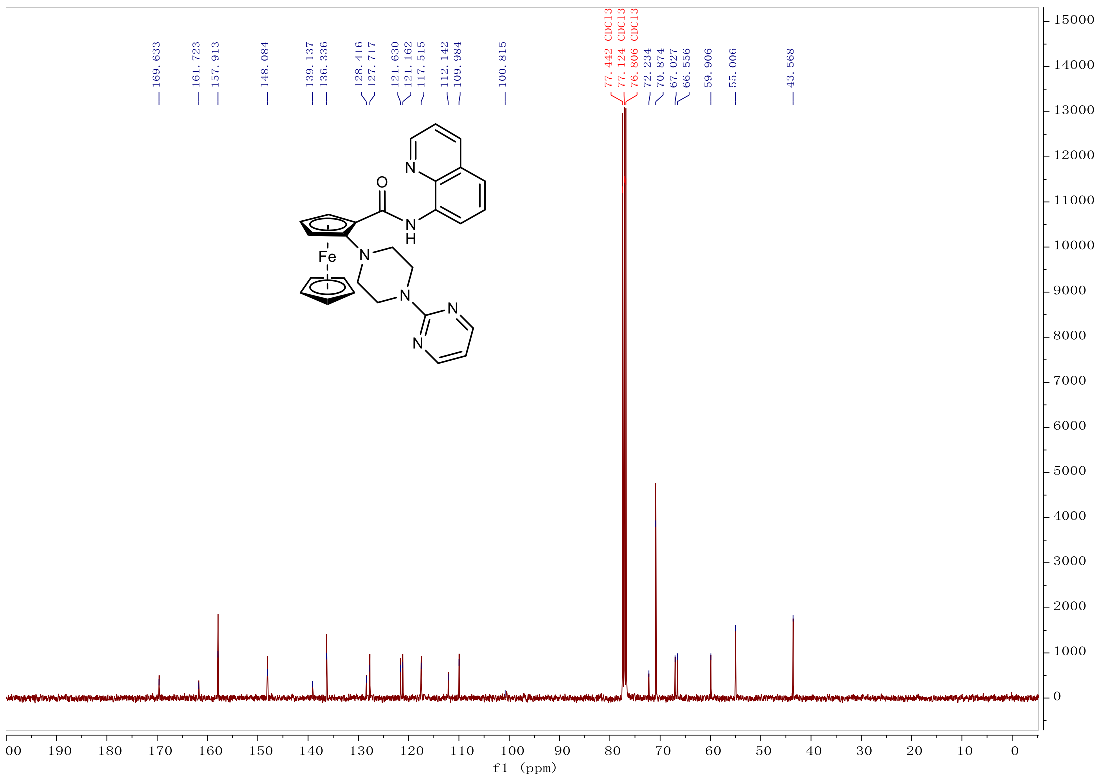

## 4r-$^{1}$H NMR

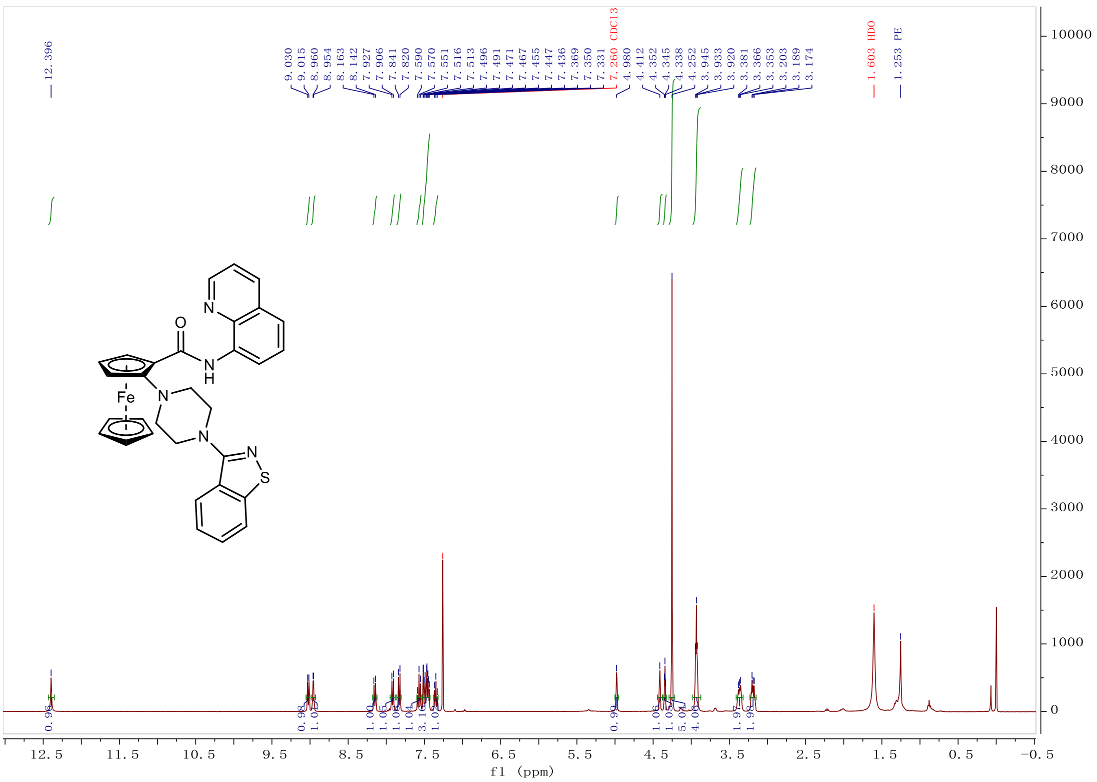

## 4r-$^{13}$C NMR

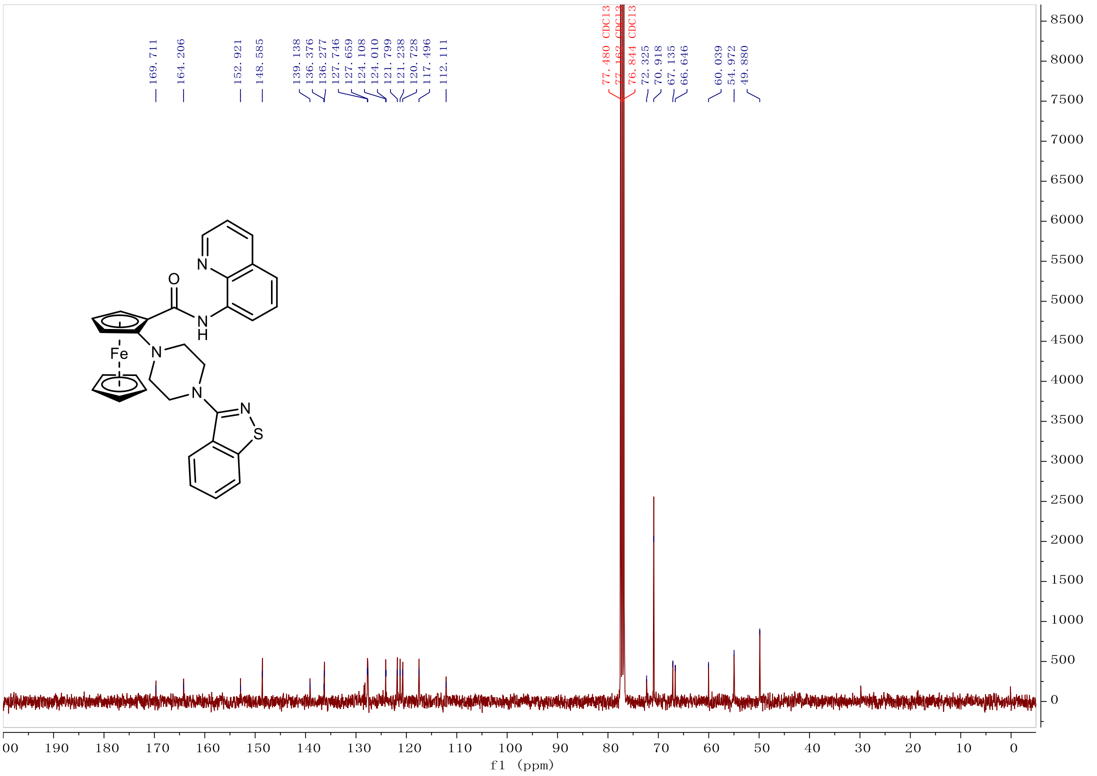

## 5-$^1$H NMR

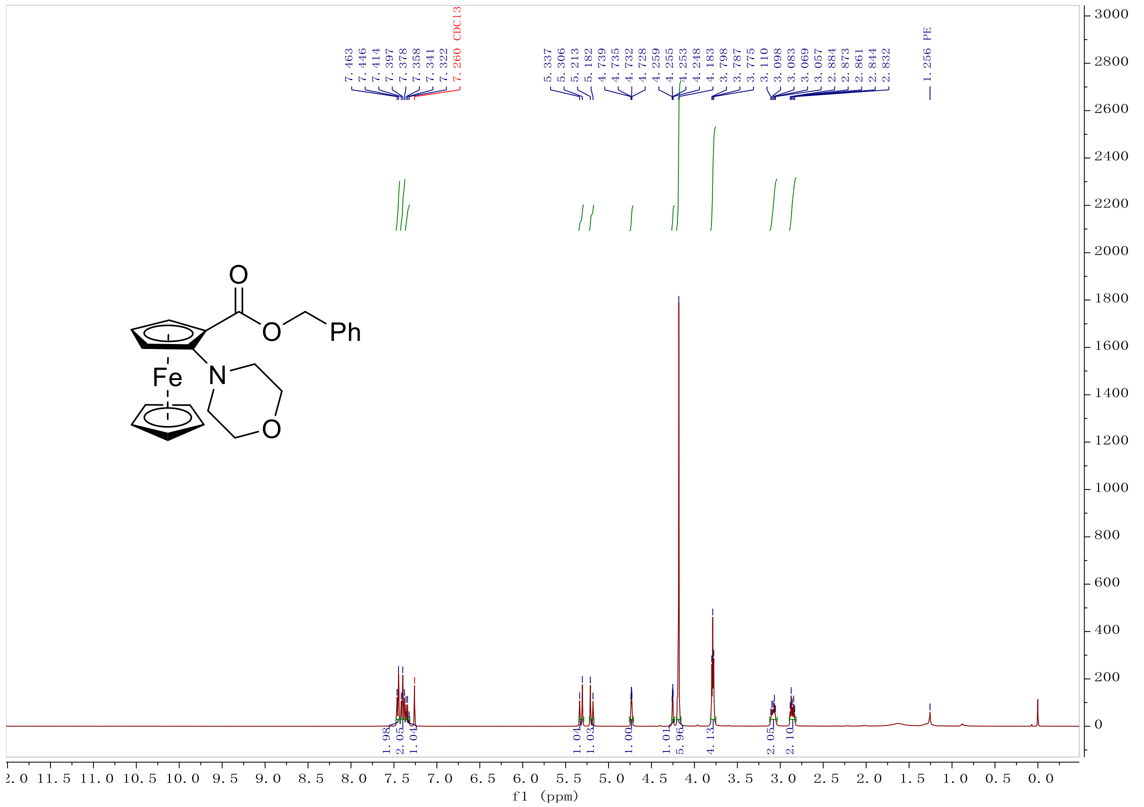

## 5-$^{13}$C NMR

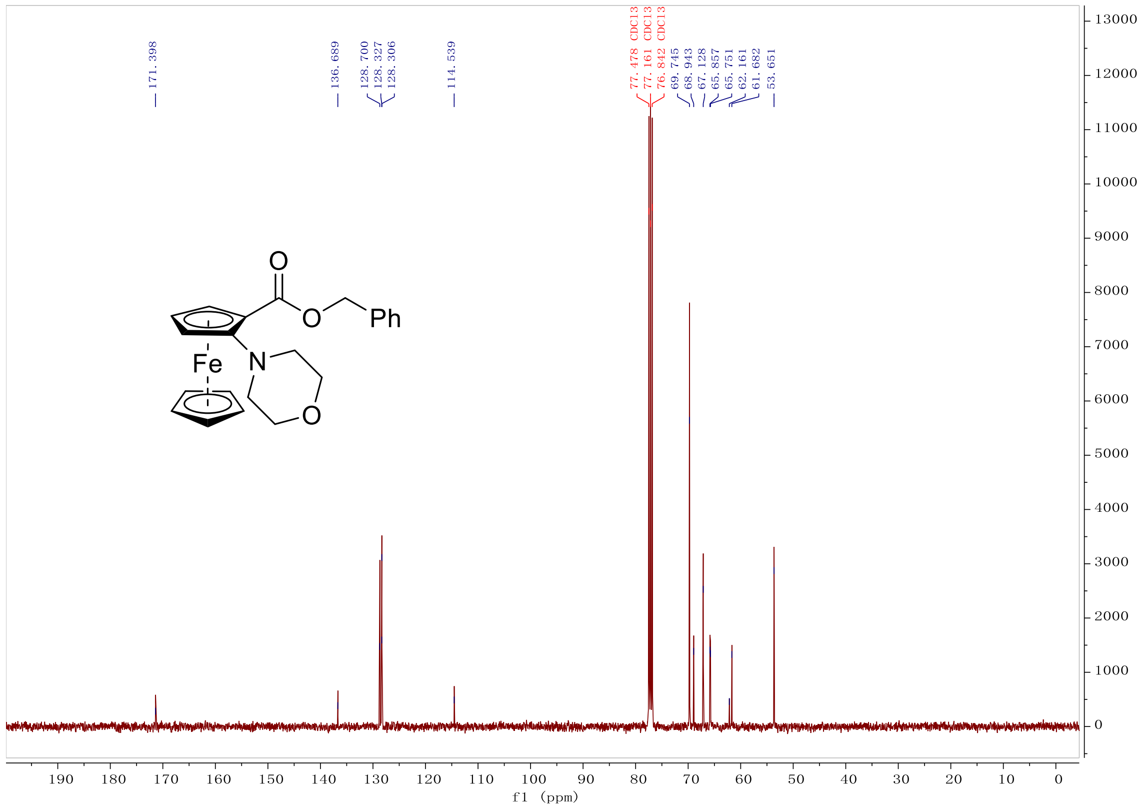

Supplement: File 1 — Full experimental details, compound characterization, and copies of NMR spectra. [file Beilstein_J_Org_Chem-17-2488-s001.pdf]
